# Bidirectional Molecular Motors by Controlling Threading and Dethreading Pathways of a Linked Rotaxane

**DOI:** 10.1002/anie.202414307

**Published:** 2024-10-30

**Authors:** Hiromichi V. Miyagishi, Hiroshi Masai, Jun Terao

**Affiliations:** ^1^ Department of Basic Science Graduate School of Arts and Sciences The University of Tokyo 3-8-1, Komaba Meguro-ku Tokyo 153-8902 Japan; ^2^ Department of Chemistry Faculty of Science Hokkaido University Kita-10 Nishi-8 Kita-ku Sapporo 060-0810 Japan; ^3^ PRESTO Japan Science and Technology Agency 4-1-8, Honcho Kawaguchi Saitama 332-0012 Japan

**Keywords:** Molecular motors, Molecular machines, Linked rotaxanes, cyclodextrins

## Abstract

Artificial molecular motors have been presented as models for biological molecular motors. In contrast to the conventional artificial molecular motors that rely on covalent bond rotation, molecular motors with mechanically interlocked molecules (MIMs) have attracted considerable attention owing to their ability to generate significant rotational motion by dynamically shuttling macrocyclic components. The topology of MIM‐type rotational molecular motors is currently limited to catenane structures, which require intricate synthetic procedures that typically produce a low synthetic yield. In this study, we develop a novel class of MIM‐type molecular motors with a rotaxane‐type topology. The switching of the threading/dethreading pathways of the linked rotaxane by protecting/deprotecting the bulky stopper group and changing the solvent polarity enables a net unidirectional rotation of the molecular motor. The threading/dethreading reaction rates were quantitatively evaluated through detailed spectroscopic investigations. Repeated net unidirectional rotation and switching of the direction of rotation were also achieved. Our findings demonstrate that linked rotaxanes can serve as MIM‐type molecular motors with reversible rotational direction controlled by threading/dethreading reactions. These motors hold potential as components of molecular machinery.

## Graphical abstract

A novel class of mechanically interlocked molecule (MIM)‐type molecular motors with a rotaxane‐type topology has been developed. The switching of the threading/dethreading pathways of the linked rotaxane by protecting/deprotecting with the bulky stopper group and changing the solvent polarity enables a net unidirectional rotation. Repeated net unidirectional rotation and switching of the direction were also achieved.

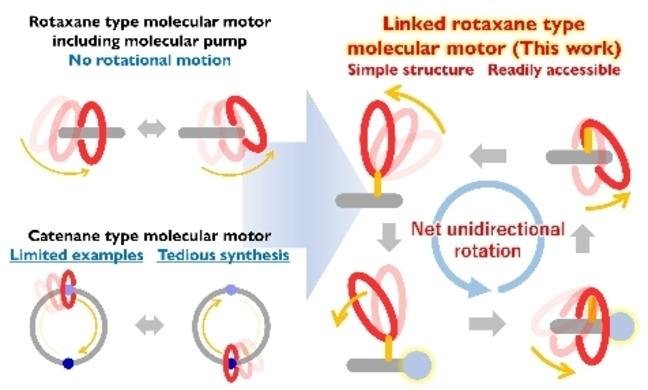

Molecular motors, which are nanomachines that convert chemical energy to rotational energy, are ubiquitous in biological systems.[^1^, ^2^] Artificial molecular motors provide insights into the dynamics of biological systems at the molecular level and have attracted significant attention owing to their potential application in dynamic functional materials.[^3^, ^4^, ^5^, ^6^, ^7^] Molecular motor systems can be constructed using several scaffolds. The earliest artificial molecular motors relied on light‐driven unidirectional rotation around a double bond[^8^, ^9^, ^10^, ^11^, ^12^] or chemical fuel‐induced unidirectional rotation around a single bond[^13^, ^14^, ^15^, ^16^, ^17^] (Figure 1a). Several dynamic materials have been developed, including actuators, that incorporate these motors.[^18^, ^19^, ^20^]

**Figure 1:**
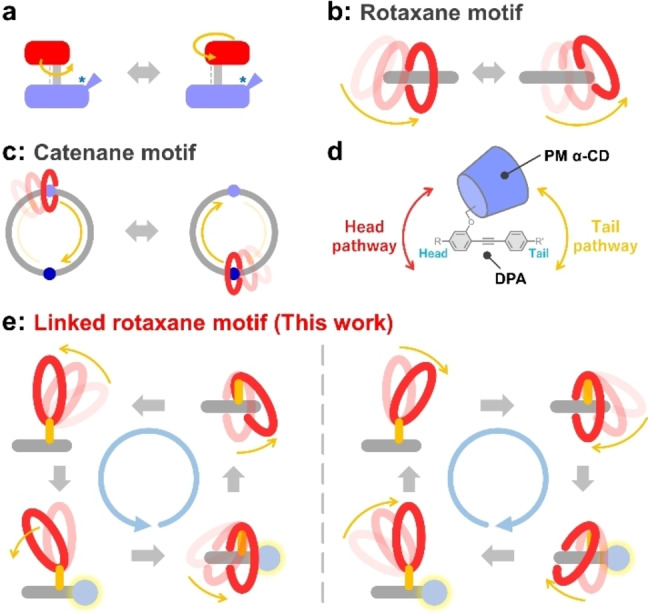
Schematic illustration of (a) single/double bond‐type molecular motors, (b) unidirectional linear motion of pseudorotaxane macrocycles, and (c) catenane‐type molecular motors. (d) Structure of a linked rotaxane with PM α‐CD and DPA. (e) Schematic illustration of a linked rotaxane‐type molecular motor.

Furthermore, a newer class of artificial molecular motors comprising mechanically interlocked molecules (MIMs) has attracted significant attention, as these motors undergo large rotational motion driven by the dynamic shuttling of macrocyclic components.[^21^, ^22^, ^23^, ^24^, ^25^, ^26^] The unidirectional rotational motion of MIM‐type molecular motors is driven by the ratchet mechanism.[^3^, ^27^] The fundamental unidirectional motion of macrocycles has been thoroughly exploited in molecular pumps, and several examples of unidirectional motion along the axles of rotaxanes and pseudorotaxanes have been reported.[^7^, ^24^, ^28^, ^29^, ^30^, ^31^, ^32^, ^33^, ^34^, ^35^, ^36^, ^37^, ^38^] However, the rotational cycle of motion in rotaxanes can only be achieved by the dethreading of the macrocycle from the axle, owing to the limited axle length (Figure 1b); thus, rotaxanes are unsuitable for generating rotational motion. This problem can be overcome by connecting the ends of the axle to form a catenane structure (Figure 1c).^[3]^ The cyclic topology of the “rail” component enables the macrocycle to undergo infinite unidirectional rotational motion. However, this strategy limits the topology of MIM‐type molecular motors to catenane‐based motifs, which, in turn, limits the scope of application of these motors as catenane‐type molecular motors typically require an intricate synthesis owing to the size of their interlocked complex structures. The development of MIM‐type molecular motors with simple structures is therefore greatly desired.

Our group has developed numerous linked rotaxanes, a subclass of rotaxanes also known as [1]rotaxanes,[^39^, ^40^, ^41^] using permethylated α‐cyclodextrin (PM α‐CD) and diphenylacetylene (DPA).[^42^, ^43^, ^44^, ^45^, ^46^, ^47^, ^48^, ^49^, ^50^] The linked rotaxane can be switched between the inclusion and uninclusion structures by adjusting the solvent polarity (Figure 1d).[^43^, ^51^, ^52^, ^53^, ^54^] The threading and dethreading processes occur via two pathways: the head and tail pathways, respectively. The threading/dethreading rates of these pathways are influenced by the size of the substituent on the DPA.[^52^, ^54^] We therefore expected that alternating between threading via the head pathway and dethreading via the tail pathway in PM α‐CD would generate rotational motion. Thus, we have proposed a novel MIM‐type molecular motor paradigm based on the linked rotaxane structure (Figure 1e). The dethreading process does not decompose the linked rotaxane structure into its constituent ring and axle components; thus, net unidirectional rotation can be achieved by conducting successive selective threading/dethreading reactions. Protecting and deprotecting the bulky Fmoc groups and changing the solvent polarity instigates threading through the head pathway and dethreading through the tail pathway, thereby inducing rotational motion.

The structure and rotational mechanisms of linked rotaxane‐type molecular motors are shown in Figure 2a. The introduction of a bulky Fmoc group at one end of DPA inhibits threading/dethreading via the tail pathway; thus, the reaction proceeds via the head pathway on the other side of DPA, which contains a small, inert methyl group. Threading via the head pathway involves the sterically unfavorable rotation of the glucose moiety, resulting in a high activation barrier (>100 kJ mol^−1^) and a slower reaction (Figure 2b).^[52]^ In contrast, removing the Fmoc protecting group to generate the amino group increases the rate of threading/dethreading via the tail pathway such that this mechanism is favored over the head pathway, resulting in threading/dethreading reactions via the tail pathway. The pathway for threading/dethreading depends on the steric bulk of the tail end of the DPA; hence, net unidirectional rotation of the PM α‐CD ring around the DPA axle can be achieved by inserting and removing the Fmoc protecting groups to switch the threading/dethreading pathways and by alternating the solvent polarity to control the equilibrium between the inclusion and uninclusion structures (Figures 1e, 2)[^52^, ^54^] Net unidirectional rotation proceeds via a mechanism that follows these four steps, (i) self‐inclusion reaction of **uninc‐Fmoc** via the head pathway yields **inc‐Fmoc**; (ii) the Fmoc group of **inc‐Fmoc** is deprotected to yield **inc**; (iii) **inc** is dethreaded via the tail pathway to yield **uninc**; (iv) **uninc** is protected with Fmoc to yield **uninc‐Fmoc**. The molecular motor rotates either clockwise or counterclockwise, depending on the orientation of the DPA moiety. Therefore, the rotation is classified as either forward or reverse rotation. The aforementioned rotation corresponds to the forward rotation.

**Figure 2:**
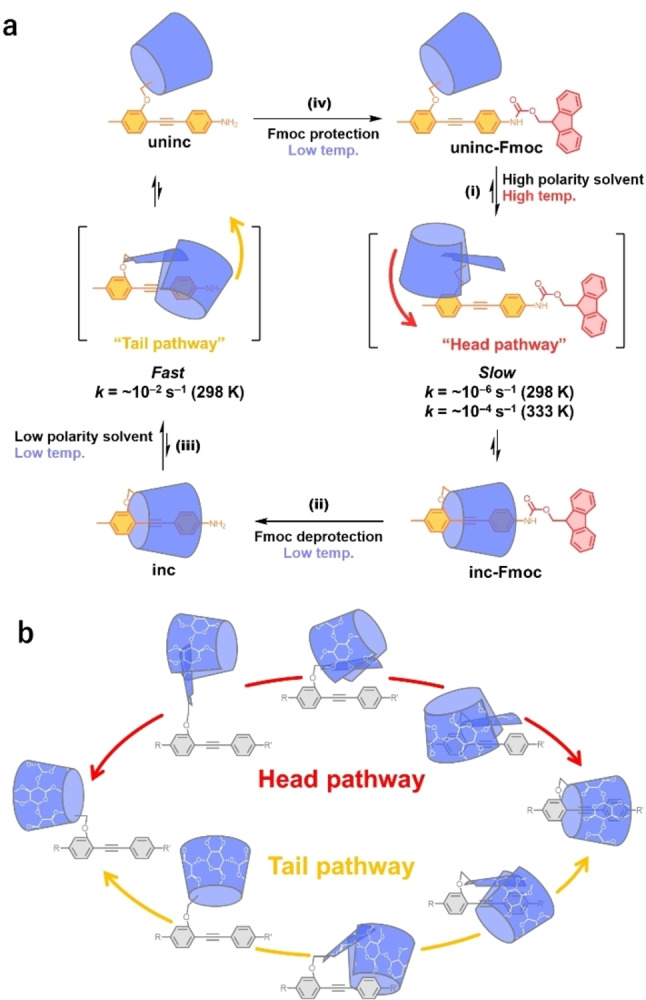
(a) Mechanism of net unidirectional rotation in the linked rotaxane‐type molecular motor. (i) **Uninc‐Fmoc** undergoes a self‐inclusion reaction via the head pathway to yield **inc‐Fmoc**, followed by (ii) Fmoc deprotection to yield **inc**, (iii) dethreading of **inc** via the tail pathway to yield **uninc**, and (iv) protection of **uninc** with Fmoc to yield **uninc‐Fmoc**. (b) Schematic illustration of the ‘head’ and ‘tail’ threading/dethreading pathways in a linked rotaxane. The head pathway involves the sterically unfavorable rotation of the glucose moiety.

The synthesis of **uninc** and **uninc‐Fmoc** is depicted in Figure 3a. Williamson ether synthesis, using **1** and 6‐*O*‐monotosyl PM α‐CD, followed by Sonogashira coupling, yielded **3**. The subsequent reduction of the nitro group and the addition of the Fmoc protection group produced, in four steps, **uninc‐Fmoc** in 68 % overall yield. Relative to previous findings, this synthesis of molecular motors is notably simple and efficient.[^21^, ^22^, ^23^, ^24^, ^25^, ^26^] In addition, a control molecule containing a ^*t*^Bu group instead of a Me group, which is referred to as **uninc/inc‐** ^***t***^ **Bu‐Fmoc**, was synthesized using a procedure similar to that of **uninc/inc‐Fmoc**.

The first step in achieving net unidirectional rotation involves using the self‐inclusion reaction via the head pathway by heating **uninc‐Fmoc** in a mixed solvent of H_2_O and methanol (Figure 3). The proton signals from the inner cavity of PM α‐CD shifted downfield during the self‐inclusion reaction, which suggests that a linked rotaxane structure was formed (Figure 3b).^[48]^ In addition, the mechanostereochemistry of **uninc/inc‐Fmoc** was determined from their ROESY spectra (Figure S1). The ROESY spectrum of **inc‐Fmoc** showed strong nuclear Overhauser effects (NOE) between the DPA protons and the H_5_ protons located in the cavity of PM α‐CD. In contrast, the **uninc‐Fmoc** ROESY spectrum showed NOE between the protons of DPA and the H_4_ protons located outside the cavity of PM α‐CD. These results suggest that **inc‐Fmoc** forms a linked rotaxane structure. To investigate the mechanism of the self‐inclusion reaction, the ^1^H NMR spectra of **uninc/inc‐Fmoc** and **uninc/inc‐** ^***t***^ **Bu‐Fmoc** in CD_3_OD were measured at 333 K (Figures 3c, S2, S3). The kinetic parameters were evaluated using nonlinear least‐squares fitting, assuming a first‐order reaction. The threading rate *k*_in_ (2.8×10^−4^ s^−1^ at 333 K) was similar to the expected rate of threading/dethreading via the head pathway.^[52]^ Conversely, **uninc‐** ^***t***^ **Bu‐Fmoc**, which has a bulky ^*t*^Bu group at the header position, did not undergo a self‐inclusion reaction, which demonstrated that the tail pathway over the Fmoc group did not proceed under the condition. These results therefore indicate that the self‐inclusion reactions of **uninc/inc‐Fmoc** proceed through the head pathway.

**Figure 3:**
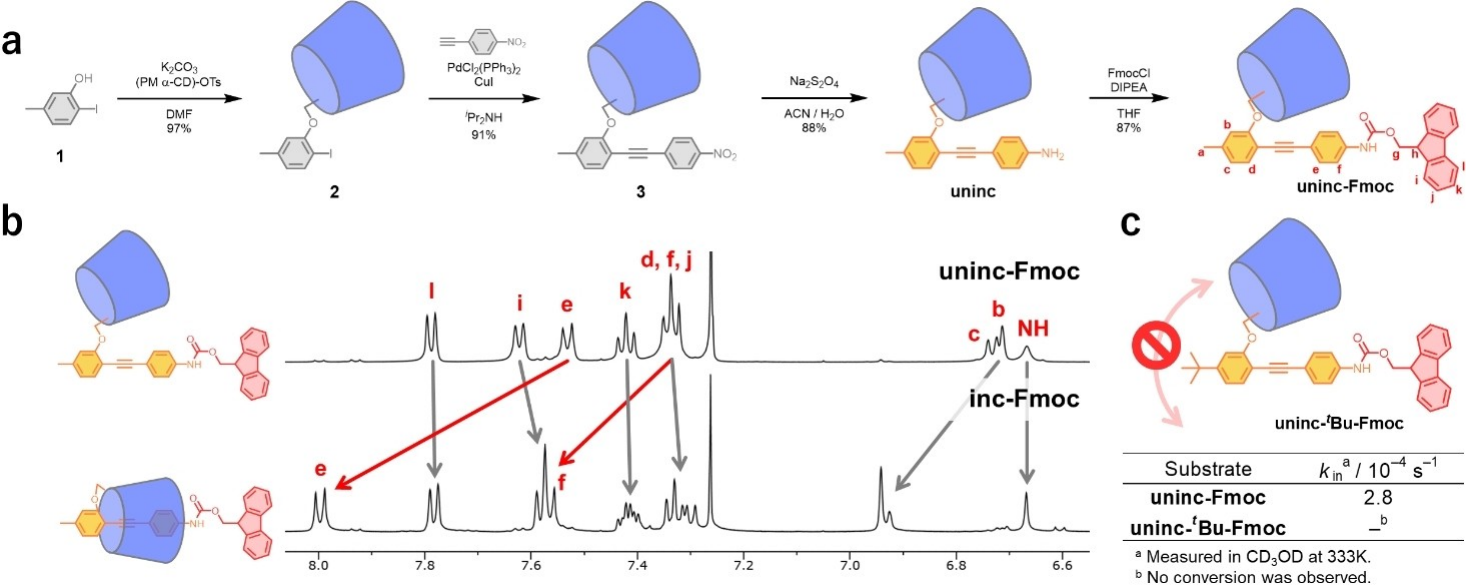
(a) Synthesis of **uninc** and **uninc‐Fmoc**. (b) ^1^H NMR spectra (500 MHz, CDCl_3_, 301–302 K) before and after the self‐inclusion reaction in H_2_O/MeOH (2 : 1) at 373 K. (c) Rate constants for the self‐inclusion reaction between **uninc/inc‐Fmoc** and between **uninc/inc‐** ^***t***^ **Bu‐Fmoc** in CD_3_OD at 333 K.

To facilitate dethreading via the tail pathway, the Fmoc protecting group was removed from **inc‐Fmoc** in a solution of piperidine in THF (Figure 4a). **Inc‐Fmoc** was completely converted to **uninc** within 10 min using a solution of 10 % (v/v) piperidine in THF (Figure 4b). The *k*_obs_ for this solution was estimated to be greater than 7.7×10^−3^ s^−1^, assuming that the reaction proceeded to 99 % completion within 10 min, whereas *t*_1/2_ was less than 1.5 min. To confirm that the conversion of **inc‐Fmoc** to **uninc** proceeded through the tail pathway, the reaction rates of each step were evaluated (Figures 4, S4, and S5). The dethreading of **inc** in THF‐*d*_8_ proceeded rapidly, as indicated by the high rate constant (*k*_out_>1.3×10^−2^ s^−1^, *t*_1/2_<1 min). The rapid dethreading of similar linked rotaxanes has been shown to proceed via the tail pathway.^[54]^ In contrast, the dethreading of **inc‐Fmoc** was very slow (*k*_out_=1.4×10^−6^ s^−1^ and *t*_1/2_=5.8 d), which indicated that dethreading via the head pathway was associated with a high activation barrier. The conversion from **inc‐Fmoc** to **uninc** via the tail pathway was more than 1000 times faster than that through the head pathway (7.7×10^−3^ s^−1^ vs. 1.4×10^−6^ s^−1^, Figure 4a), which demonstrated that dethreading via the tail pathway dominates under these conditions. This kinetic asymmetry defines the direction of rotational motion.

**Figure 4:**
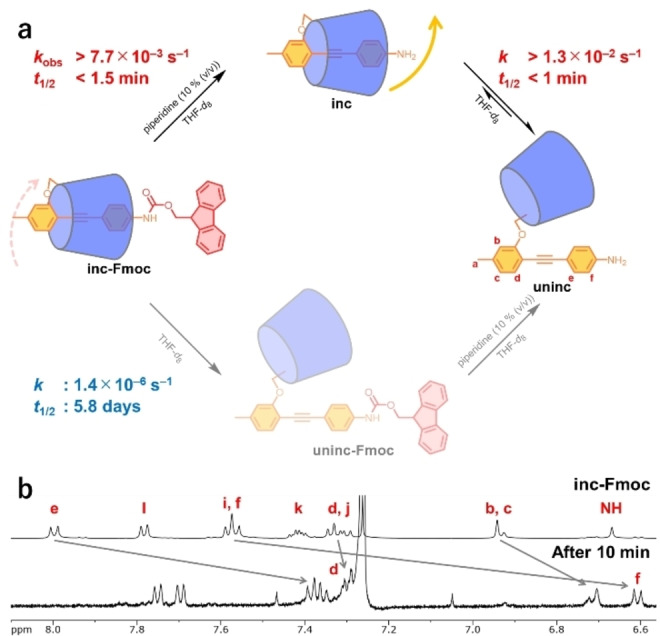
(a) Deprotection and subsequent dethreading of **inc‐Fmoc**. **inc‐Fmoc** can be converted into **uninc** via two possible routes. (b) ^1^H NMR spectra before and after the deprotection and dethreading reactions in a solution of 10 % (v/v) piperidine in THF (500 MHz, CDCl_3_, 303 K).

In the final step, the initial state of the molecular motor was realized by introducing the bulky Fmoc protecting group into the tail end of **uninc** to yield **uninc‐Fmoc** (Figure S6). An ^1^H NMR spectroscopic analysis confirmed the formation of **uninc‐Fmoc** and revealed that **uninc** was fully protected within 60 min. These results demonstrate that the PM α‐CD moiety of the linked rotaxane underwent net unidirectional rotation around DPA driven by successive Fmoc protection/deprotection and threading/dethreading reactions.

Additionally, the net unidirectional rotation of the linked‐rotaxane‐type motors was achieved in a one‐pot system (Figure 5a, b). First, **uninc** was protected by Fmoc in THF to yield **uninc‐Fmoc** (step a), to which MeOH/H_2_O was added; the mixture was then heated at 100 °C to afford **inc‐Fmoc** (step b). The solvents were then removed before a solution of 10 % (v/v) piperidine in THF was added to remove the Fmoc protecting group, which enabled the dethreading reaction of **inc** to proceed, yielding **uninc**, the initial state of the molecular motor (step c). Several washing procedures were required in the final step to remove trace amounts of piperidine, resulting in undesirable Fmoc deprotection. The one‐pot reactions were observed using ^1^H NMR spectroscopy, which clearly indicated the alternate formation of **uninc**, **uninc‐Fmoc**, and **inc‐Fmoc** at each step. Four full rotations of the α‐CD moiety were performed sustainably (Figure 5b). These results demonstrate that linked‐rotaxane‐type molecular motors unambiguously undergo net unidirectional rotation in a one‐pot system.

**Figure 5:**
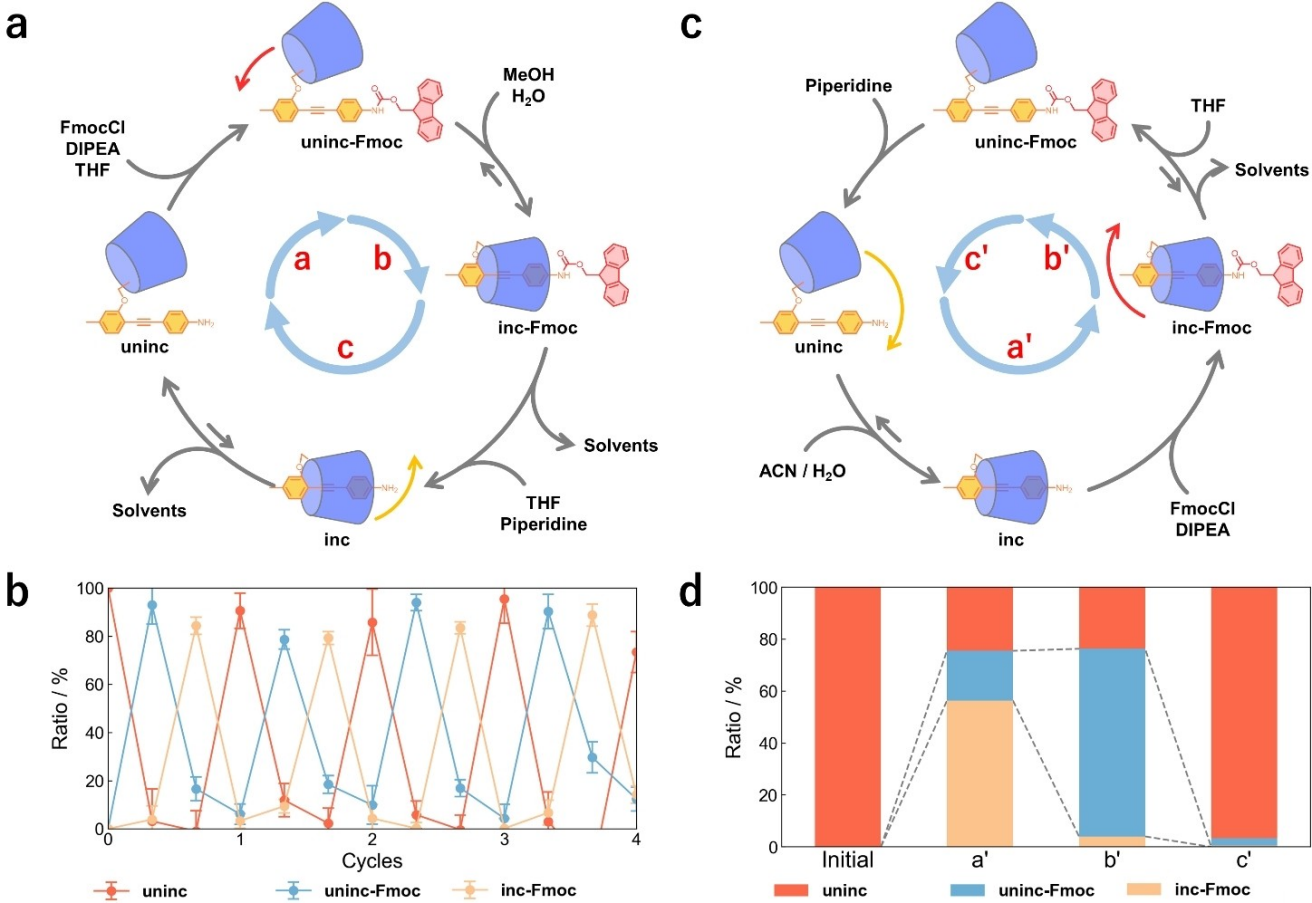
(a) Net unidirectional rotation of the PM α‐CD of **uninc** in a one‐pot system and (b) abundance ratio of **uninc**, **uninc‐Fmoc** and **inc‐Fmoc** in each step of the net unidirectional rotation process. The error bars represent *σ*. (c) Net unidirectional rotation of the PM α‐CD of **uninc** in the reverse direction and (d) abundance ratio of **uninc**, **uninc‐Fmoc**, and **inc‐Fmoc** in each step. a: FmocCl, DIPEA in THF, 60 °C, 1 h; b: THF/MeOH/H_2_O (1 : 6 : 9), 100 °C, 15 min, followed by solvent evaporation; c: piperidine in THF, 25 °C, 1 h, followed by solvent evaporation, extraction by toluene/sat. NH_4_Cl aq., and further solvent evaporation; a′: FmocCl, DIPEA in ACN/H_2_O (1 : 1), 60 °C, 1 h, followed by solvent evaporation, b′: THF, 100 °C, 15 min, c′: piperidine in THF, 25 °C, 30 min.

Finally, we demonstrated net unidirectional rotation in the reverse direction (Figure 5c, d). The molecular motor allowed two rotational modes; the “normal” direction, which comprised the threading process via the head pathway and the dethreading process via the tail pathway, and the “reversed” direction, which comprised the dethreading process via the head pathway and the threading process via the tail pathway. In the reversed directional rotation, the rotation of linked‐rotaxane‐type motors occurred via the threading of **uninc** in ACN/H_2_O followed by the protection of **inc** with Fmoc (step a’), the dethreading of **inc‐Fmoc** in THF (step b’), and the deprotection of **uninc‐Fmoc** (step c’). Similar to the discussion of normal direction, **inc‐** ^***t***^ **Bu‐Fmoc** did not undergo dethreading in THF, which demonstrated that dethreading via the tail pathway over the Fmoc group did not proceed under the condition (Figure S9). Although there remained a non‐negligible amount of **uninc** and **uninc‐Fmoc** that did not undergo net unidirectional rotation, the linked‐rotaxane‐type motors mostly underwent net unidirectional rotation in the reverse direction (Figure 5d). The presence of residual **uninc** and **uninc‐Fmoc** was attributed to the low polarity of the ACN/H_2_O (1 : 1) solvent system; however, this solvent system is considered optimal because further increasing the solvent polarity induces phase separation, which reduces the yield of **inc‐Fmoc**. The switching of the net unidirectional rotation direction of MIM‐type molecular motors, which is a rare phenomenon,[^53^, ^54^, ^55^, ^56^, ^57^, ^58^] demonstrates the advantage of molecular motors driven by sequential chemical reactions.

In this work, a rotaxane‐type molecular motor was developed using PM α‐CD and DPA. The protection/deprotection of the rotaxane structure using Fmoc enabled selective threading/dethreading reactions, which resulted in a net unidirectional rotation of the PM α‐CD moiety around DPA. The reaction rates at each step were indicative of the net unidirectional motion of the motor. In addition, the net unidirectional rotation of the motor in both directions was induced using a one‐pot process. The structure of the linked‐rotaxane‐type molecular motor is less complex than those of conventional catenane‐type molecular motor, and this facilitates the application of MIM‐type molecular motors in dynamic functional materials.

## Supporting Information

The authors have cited additional references within the Supporting Information.[^55^, ^56^, ^57^, ^58^, ^59^, ^60^]

## Conflict of Interests

The authors declare no conflict of interest.

## Supporting information

As a service to our authors and readers, this journal provides supporting information supplied by the authors. Such materials are peer reviewed and may be re‐organized for online delivery, but are not copy‐edited or typeset. Technical support issues arising from supporting information (other than missing files) should be addressed to the authors.

Supporting Information

## Data Availability

The data that support the findings of this study are available in the supplementary material of this article.
