# Supplementary material for: Bidirectional Molecular Motors by Controlling Threading and Dethreading Pathways of a Linked Rotaxane
Source: Angew Chem Int Ed Engl. 2024 Oct 30;64(2):e202414307. doi: 10.1002/anie.202414307 (PMC11720386; doi:10.1002/anie.202414307)
Supplement: Supplementary file 1 — Supporting Information [file ANIE-64-e202414307-s001.pdf]

Angewandte Chemie
Eine Zeitschrift der Gesellschaft Deutscher Chemiker

Supporting Information

Bidirectional Molecular Motors by Controlling Threading and Dethreading Pathways of a Linked Rotaxane

*H. V. Miyagishi, H. Masai, J. Terao**

Supporting Information

Bidirectional Molecular Motors by Controlling Threading and Dethreading Pathways of a Linked Rotaxane

Hiromichi V. Miyagishi[a,b], Hiroshi Masai[a,c], and Jun Terao[a,*]

[a] Department of Basic Science, Graduate School of Arts and Sciences,
The University of Tokyo.
[b] Current affiliation: Department of Chemistry, Faculty of Science, Hokkaido University.
[c] PRESTO, Japan Science and Technology Agency.
*Correspondence: cterao@mail.ecc.u-tokyo.ac.jp

Table of Contents

1. General Remarks 4
   1.1 Materials 4
   1.2 Experimental equipment 4
      1.2.1 NMR spectroscopy 4
      1.2.2 High-Resolution Mass Spectroscopy (HR-MS) 4
      1.2.3 Preparative recycling gel permeation chromatography 4
      1.2.4 Flash column chromatography 5
2. Experimental procedures 5
   2.1 Synthesis of **uninc** 5
   2.2 Synthesis of **uninc-Fmoc** 7
   2.3 Synthesis of **inc-Fmoc** 8
   2.4 Synthesis of **inc** 9
   2.5 Synthesis of **uninc-*t*Bu** 10
   2.6 Synthesis of **uninc-*t*Bu-Fmoc** 12
   2.7 Synthesis of **inc-*t*Bu-Fmoc** 13
   2.8 Net unidirectional rotation in a one-pot manner 13
   2.9 Net unidirectional rotation in the reverse direction 14
3. NMR measurements 15
   3.1 ROESY spectra of **uninc-Fmoc** and **inc-Fmoc** 15
   3.2 Threading reaction of **uninc-Fmoc** 15
   3.3 Heating experiment of **uninc-*t*Bu-Fmoc** in CD_3OD 17
   3.4 Dethreading reaction of **inc** 17
   3.5 Fmoc deprotection of **inc-Fmoc** 18
   3.6 Dethreading reaction of **inc-Fmoc** 18
   3.7 Fmoc protection of **uninc** 20
   3.8 One-pot net unidirectional rotation 21
   3.9 Net unidirectional rotation in the reverse direction 23
   3.10 Heating experiment of **inc-*t*Bu-Fmoc** in THF 25
4. Compound Data 26
   4.1 NMR spectra of compound **2** 26
   4.2 NMR spectra of **3** 27
   4.3 NMR spectra of **uninc** 28
   4.4 NMR spectra of **uninc-Fmoc** 32
   4.5 NMR spectra of **inc-Fmoc** 35
   4.6 NMR spectra of **inc** 38
   4.7 NMR spectra of **S2** 42

4.8 NMR spectra of **S3** 43
4.9 NMR spectra of **uninc-^{t}Bu** 44
4.10 NMR spectra of **uninc-^{t}Bu-Fmoc** 45
4.11 NMR spectra of **inc-^{t}Bu-Fmoc** 46
4.12 HR-MS spectra 48
5. References 53

1. General Remarks

1.1 Materials

Unless otherwise noted, manipulations were performed under nitrogen atmosphere using standard Schlenk-type glassware in a dual-manifold Schlenk line. Unless otherwise stated, commercially available chemicals were used as received. Dry tetrahydrofuran were purchased from Kanto Chemical and further purified by passage through activated alumina under positive nitrogen pressure as described by Grubbs et al.[1] $^{i}Pr_2NH$ were degassed by nitrogen bubbling, before using. Compound **1**,[2] 1-ethynyl-4-nitrobenzene,[3] 6-*O*-monotosyl PM α-CD[4], compound **S1**[5] was prepared according to the previously reported procedure.

1.2 Experimental equipment

1.2.1 NMR spectroscopy

^{1}H NMR (500 MHz) and ^{13}C {^{1}H} NMR (126 MHz) spectra were measured with a Bruker AVANCE III HD 500. The ^{1}H NMR chemical shifts are reported relative to tetramethylsilane (0.00 ppm) or residual protonated solvents (7.26 ppm for $CHCl_3$, 1.72 ppm for THF-d_8). The ^{13}C NMR chemical shifts are reported relative to tetramethylsilane (0.00 ppm) or deuterated solvents (77.16 ppm for $CDCl_3$, 25.3 ppm for THF-d_8).

1.2.2 High-Resolution Mass Spectroscopy (HR-MS)

Electrospray ionization time-of-flight (ESI-ToF) mass spectra were obtained using a Bruker micrOTOF II-KE02. The HR-MS spectra were internally calibrated using NaTFA clusters.

1.2.3 Preparative recycling gel permeation chromatography

Preparative recycling GPC was performed using the following equipment.

System 1
System: JAI LaboACE LC-5060
Detector: JAI UV-VIS 4ch 400HM and JAI RI-700HM
Column: JAIGEL-2HH
Eluent: $CHCl_3$
Flow rate: 7.5 mL min^{-1}

System 2
System: SHIMADZU LC-20AP
Detector: SHIMADZU SPD-20A and SHIMADZU RID-10A
Column: Shodex K-4002L
Eluent: $CHCl_3$
Flow rate: 14 mL min^{-1}

1.2.4 Flash column chromatography

Flash column chromatography was performed using Isolera One.

2. Experimental procedures

2.1 Synthesis of uninc

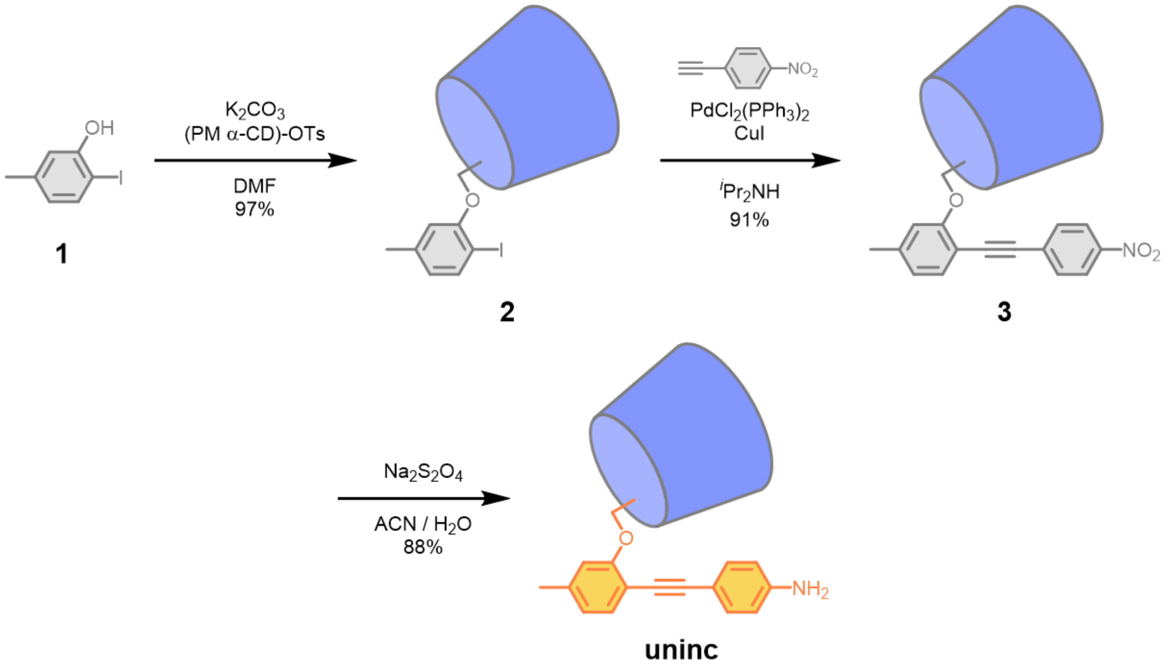

Scheme S1. Synthesis of **uninc**.

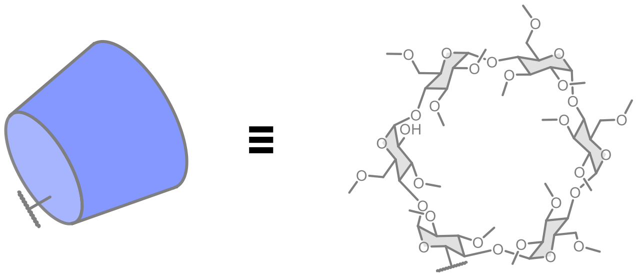

PM α-CD

Scheme S2. Chemical structure of PM α-CD.

2. A mixture of **1** (222 mg, 950 μmol, 1.27 eq.), 6-*O*-monotosyl PM α-CD (1.02 g, 749 μmol), and dry K_2CO_3 (208 mg, 1.50 mmol, 2 eq.) in dehydrated DMF (30 mL) was stirred for 16 h at 85 °C. After the mixture cooled to room temperature, brine (150 mL) and distilled water (50 mL) was added. The mixture was extracted with ethyl acetate (3 × 100 mL). The organic layers were combined, washed with brine (5 × 150 mL), dried over $MgSO_4$, filtered, and concentrated under reduced pressure. The residue was purified by flash column chromatography (hexane/50%→100% EtOAc, then EtOAc/0%→14% MeOH) to give **S2** as a white foam (1.03 g, 724 μmol, 97%).

^{1}**H NMR** (500 MHz, $CDCl_3$) δ 7.63 (d, *J* = 7.9 Hz, 1H, ArH), 6.69 (s, 1H, ArH), 6.60 (d, *J* = 8.1 Hz, 1H, ArH), 5.10 (d, *J* = 3.2 Hz, 1H, H_{CD-1}), 5.09 (d, *J* = 3.3 Hz, 1H, H_{CD-1}), 5.07 (d, *J* = 3.0 Hz, 3H, H_{CD-1}), 5.00 (d, *J* = 3.1 Hz, 1H, H_{CD-1}), 4.41-4.33 (m, 2H, H_{CD-6}), 4.22 (dd, *J* = 9.4, 5.0 Hz, 1H, H_{CD-5}), 4.15 (dd, *J* = 11.1, 2.9 Hz, 1H, H_{CD}), 3.94-3.09 (m, 82H, H_{CD}), 2.94 (d, *J* = 10.1 Hz, 1H, H_{CD}), 2.31 (s, 3H, $ArCH_3$).
^{13}**C NMR** (126 MHz, $CDCl_3$) δ 157.6, 140.1, 138.8, 124.1, 114.3, 100.56, 100.44, 100.35, 100.22 (peaks overlapped), 99.9, 83.4, 82.94, 82.81, 82.58, 82.42, 82.37 (peaks overlapped), 82.33, 82.28, 82.22 (peaks overlapped), 82.14, 81.53, 81.44, 81.40, 81.29 (peaks overlapped), 71.87, 71.80, 71.77, 71.59, 71.54, 71.49, 71.35, 71.31, 71.20 (peaks overlapped), 70.5 (peaks overlapped), 62.12, 62.01, 61.98 (peaks overlapped), 61.94, 59.27, 59.21, 59.18, 59.15, 58.8, 58.03, 57.95 (peaks overlapped), 57.90 (peaks overlapped), 57.88, 21.5.
HR-MS (ESI): *m/z* = 1449.51386 $[M+Na]^+$ (calcd. 1449.51581 for $C_{60}H_{99}IO_{30}Na$).

3. A mixture of **2** (296 mg, 207 μmol), 1-ethynyl-4-nitrobenzene (46.7 mg, 317 μmol, 1.5 eq.), $PdCl_2(PPh_3)_2$ (14.8 mg, 21.1 μmol, 10 mol%), and CuI (1.7 mg, 8.9 μmol, 4 mol%) in degassed $^iPr_2NH$ (3 mL) was stirred overnight at 40 °C, and then the solvent was removed in vacuum. The residue was purified by preparative GPC with chloroform as eluent to yield **S3** as a yellow foam (274 mg, 189 μmol, 91%).
^{1}**H NMR** (500 MHz, $CDCl_3$) δ 8.18 (d, *J* = 8.9 Hz, 2H, ArH), 7.75 (d, *J* = 8.9 Hz, 2H, ArH), 7.37 (d, *J* = 7.7 Hz, 1H, ArH), 6.77 (d, *J* = 7.8 Hz, 1H, ArH), 6.74 (s, 1H, ArH), 5.13 (d, *J* = 3.3 Hz, 1H, H_{CD-1}), 5.08 (d, *J* = 3.2 Hz, 1H, H_{CD-1}), 5.07-5.03 (m, 3H, H_{CD-1}), 4.99 (d, *J* = 3.2 Hz, 1H, H_{CD-1}), 4.69 (dd, *J* = 10.2, 2.2 Hz, 1H, H_{CD-6}), 4.32 (t, *J* = 9.2 Hz, 1H, H_{CD-4}), 4.24 (d, *J* = 10.3 Hz, 1H, H_{CD-6}), 4.03 (d, *J* = 9.3 Hz, 1H, H_{CD-5}), 3.85-3.01 (m, 83H, H_{CD}), 2.37 (s, 3H, $ArCH_3$).
^{13}**C NMR** (126 MHz, $CDCl_3$) δ 159.7, 146.8, 141.4, 133.4, 132.3, 131.0, 123.7, 121.9, 113.1, 109.2, 100.7, 100.38, 100.34, 100.31, 100.25, 99.8, 92.5, 91.5, 82.78, 82.75, 82.73 (peaks overlapped), 82.66 (peaks overlapped), 82.44, 82.38 (peaks overlapped), 82.34, 82.25, 81.9, 81.46, 81.40 (peaks overlapped), 81.37 (peaks overlapped), 81.30, 71.94, 71.92, 71.79, 71.71 (peaks overlapped), 71.50 (peaks overlapped), 71.45, 71.41, 71.31, 70.8, 67.7, 62.04, 61.98, 61.94 (peaks overlapped), 61.87, 59.32, 59.21, 59.19 (peaks overlapped), 59.04, 58.5, 58.08 (peaks overlapped), 58.02, 57.98, 57.6, 22.2.
HR-MS (ESI): *m/z* = 1468.63264 $[M+Na]^+$ (calcd. 1468.63554 for $C_{68}H_{103}NO_{32}Na$).

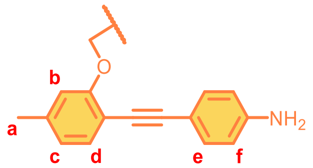

uninc. To a solution of **3** (59.8 mg, 41.3 μmol) in degassed MeCN (10 mL) and H_2O (10 mL), $Na_2S_2O_4$ (76.0 mg, 437 μmol, 11 eq.) was added and stirred for 10 min at room temperature. After the reaction ended, brine (40 mL) was added and the mixture was extracted with DCM/Et_2O (2:1, 3 × 40 mL). The organic layers were combined, dried over $MgSO_4$, filtered, and concentrated under reduced pressure. The residue was purified by preparative GPC with chloroform as eluent to yield **uninc** as a pale yellow foam

(51.6 mg, 36.4 μmol, 88%).

^{1}H NMR (500 MHz, THF-d_8) δ 7.30 (d, J = 8.4 Hz, 2H, H_e), 7.18 (d, J = 7.7 Hz, 1H, H_d), 6.77 (s, 1H, H_b), 6.65 (d, J = 7.7 Hz, 1H, H_c), 6.52 (d, J = 8.4 Hz, 2H, H_f), 5.12 (d, J = 3.3 Hz, 1H, H_{CD-1}), 5.02 (d, J = 3.3 Hz, 1H, H_{CD-1}), 4.99-4.95 (m, 3H, H_{CD-1}), 4.90 (d, J = 3.4 Hz, 1H, H_{CD-1}), 4.86 (dd, J = 10.5, 2.5 Hz, 1H, H_{CD-6}), 4.73 (s, 2H, NH_2), 4.31 (t, J = 9.2 Hz, 1H, H_{CD-4}), 4.12 (d, J = 10.4 Hz, 1H, H_{CD-6}), 3.98 (d, J = 9.5 Hz, 1H, H_{CD-5}), 3.86-2.98 (m, 82H, H_{CD}), 2.93 (dd, J = 9.7, 3.4 Hz, 1H, H_{CD-2}), 2.30 (s, 3H, H_a).

^{13}C NMR (126 MHz, THF-d_8) δ 160.1, 149.5, 139.2, 133.5, 132.5, 121.5, 114.5, 113.9, 112.2, 101.2, 100.7, 100.38, 100.37, 100.28, 99.7, 95.0, 84.1, 83.87, 83.77, 83.70 (peaks overlapped), 83.60, 83.58, 83.49, 83.45, 83.42, 83.29, 83.25, 82.57, 82.54, 82.52 (peaks overlapped), 82.48 (peaks overlapped), 82.42, 73.19, 73.07, 72.70, 72.67 (peaks overlapped), 72.22, 72.10 (peaks overlapped), 71.98 (peaks overlapped), 71.85, 68.2, 62.00, 61.86 (peaks overlapped), 61.78, 61.76, 61.73, 58.87, 58.79 (peaks overlapped), 58.75, 58.5, 58.12, 57.95, 57.90, 57.86, 57.5, 21.9.

HR-MS (ESI)**:** m/z = 1438.66017 [M+Na]$^+$ (calcd. 1438.66136 for $C_{68}H_{105}NO_{30}Na$).

2.2 Synthesis of uninc-Fmoc

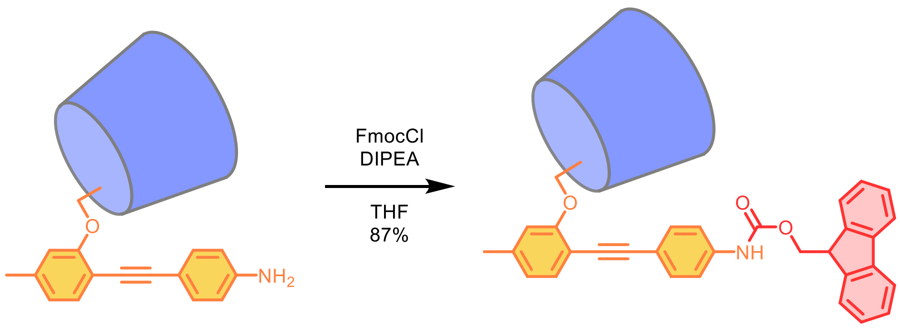

Scheme S3. Synthesis of **uninc-Fmoc**.

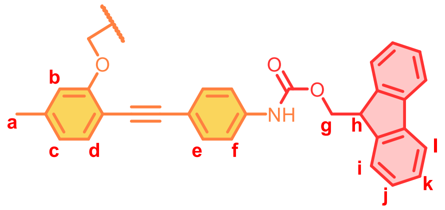

uninc-Fmoc. A mixture of **uninc** (199.8 mg, 141 μmol), FmocCl (40.7 mg, 157 μmol, 1.1 eq.), and DIPEA (26.4 μL, 155 μmol, 1.1 eq.) in dry THF (14 mL) was stirred for 1 h at 60 °C, and then the solvent was removed in vacuum. The residue was purified by preparative GPC with chloroform as eluent to yield **uninc-Fmoc** as a pale yellow foam (200.4 mg, 122 μmol, 87%).

^{1}H NMR (500 MHz, $CDCl_3$) δ 7.79 (d, J = 7.5 Hz, 2H, H_l), 7.62 (d, J = 7.4 Hz, 2H, H_i), 7.53 (d, J = 8.5 Hz, 2H, H_e), 7.42 (t, J = 7.4 Hz, 2H, H_k), 7.38-7.30 (m, 5H, H_d/H_f/H_j), 6.76-6.70 (m, 2H, H_b/H_c), 6.67 (s, 1H, NH), 5.17 (d, J = 3.1 Hz, 1H, H_{CD-1}), 5.07-5.05 (m, 4H, H_{CD-1}), 4.99 (d, J = 3.2 Hz, 1H, H_{CD-1}), 4.65 (dd, J = 10.1, 2.1 Hz, 1H, H_{CD-6}), 4.56 (d, J = 6.5 Hz, 2H, H_g), 4.32-4.27 (m, 2H, H_{CD-4}/H_h), 4.24 (d, J = 10.0 Hz, 1H,

H_{CD-6}), 4.00 (d, J = 9.0 Hz, 1H, H_{CD-5}), 3.89-2.99 (m, 83H, H_{CD}) 2.34 (s, 3H, H_a).

^{13}C NMR (126 MHz, $CDCl_3$) δ 159.3, 153.2, 143.8, 141.5, 139.6, 137.5, 133.0, 132.6, 128.0, 127.3, 125.0, 121.7, 120.2, 119.0, 118.4, 113.2, 110.5, 100.7, 100.34, 100.30 (peaks overlapped), 100.25, 99.6, 92.8, 86.0, 82.80, 82.71 (peaks overlapped), 82.62, 82.46, 82.41, 82.39, 82.38, 82.35, 82.16, 81.8, 81.41 (peaks overlapped), 81.39, 81.37, 81.28, 71.94, 71.91, 71.74, 71.68 (peaks overlapped), 71.50, 71.48, 71.45, 71.39, 71.38, 71.0, 67.6, 67.1, 61.99, 61.94 (peaks overlapped), 61.90, 61.85, 59.30, 59.19 (peaks overlapped), 59.08, 58.4, 58.06, 58.02, 58.00, 57.98, 57.4, 47.3, 22.1.

HR-MS (ESI)**:** m/z = 841.86002 $[M+2Na]^{2+}$ (calcd. 841.85933 for $C_{83}H_{115}NO_{32}Na_2$).

2.3 Synthesis of inc-Fmoc

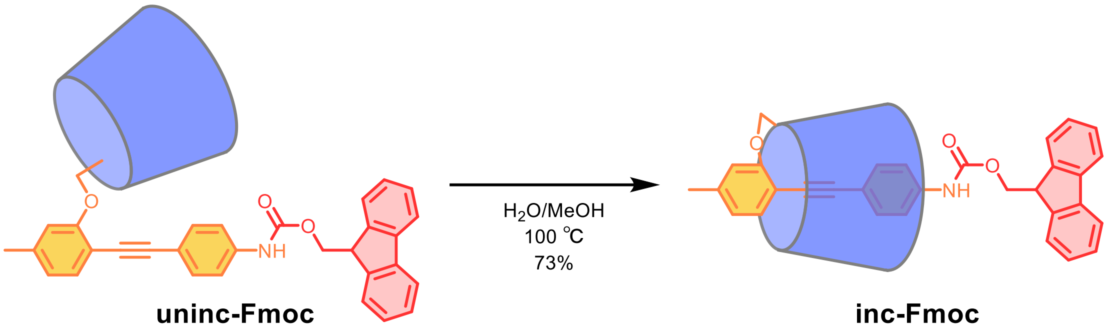

Scheme S4. Synthesis of **inc-Fmoc**.

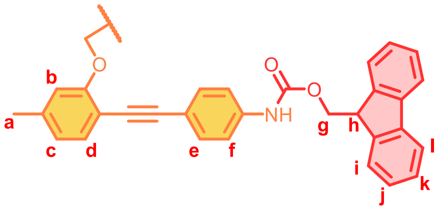

inc-Fmoc. A mixture of **uninc-Fmoc** (49.2 mg, 30.0 μmol) in MeOH (9 mL) and H_2O (9 mL) was stirred for 40 min at 100 °C using a pressure vessel. After the mixture cooled to room temperature, H_2O (4.5 mL) was added and the solution was stirred for 20 min at 100 °C. After the mixture cooled to room temperature, the solution was evaporated in vacuum. The residue was purified by preparative GPC with chloroform as eluent to yield **inc-Fmoc** as a white foam (35.8 mg, 21.8 μmol, 73%).

^{1}H NMR (500 MHz, $CDCl_3$) δ 8.00 (d, J = 8.6 Hz, 2H, H_e), 7.78 (d, J = 7.5 Hz, 2H, H_l), 7.57 (t, J = 8.2 Hz, 4H, H_f/H_i), 7.44-7.40 (m, 2H, H_k), 7.35-7.29 (m, 3H, H_d/H_j), 6.94-6.92 (m, 2H, H_b/H_c), 6.67 (s, 1H, NH), 5.08 (d, J = 3.0 Hz, 1H, H_{CD-1}), 5.05 (d, J = 3.2 Hz, 1H, H_{CD-1}), 5.00 (s, 1H, H_{CD-1}), 4.98-4.93 (m, 3H, H_{CD-1}), 4.86-4.82 (m, 1H, H_{CD-5}), 4.54 (d, J = 6.4 Hz, 2H, H_g), 4.37-4.35 (m, 1H, H_{CD-6}), 4.29 (dd, J = 11.3, 2.3 Hz, 1H, H_{CD-6}), 4.25-4.17 (m, 3H, H_o/H_{CD-5}/ H_{CD-5} or H_{CD-6}), 4.09-4.06 (m, 2H, H_{CD-5} and/or H_{CD-6}), 4.02-4.00 (m, 1H, H_{CD-5} or H_{CD-6}), 3.97-3.89 (m, 2H, H_{CD-5} and/or H_{CD-6}), 3.83-3.01 (m, 76H, H_{CD}), 2.75 (d, J = 10.3 Hz, 1H, H_{CD-6}), 2.38 (s, 3H, H_a).

^{13}C NMR (126 MHz, $CDCl_3$) δ 162.0, 152.3, 143.7, 141.5, 140.7, 138.6, 133.5, 132.9, 127.96, 127.94, 127.24, 127.22, 124.9, 124.2, 122.9, 120.2, 117.48, 117.38, 114.0, 100.9, 100.5, 100.26, 100.22, 100.08,

98.2, 93.4, 86.4, 84.1, 82.79, 82.68 (peaks overlapped), 82.61, 82.51, 82.33, 82.28 (peaks overlapped), 82.14 (peaks overlapped), 81.79, 81.62, 81.38, 81.32, 81.26 (peaks overlapped), 81.19, 76.4, 72.4, 71.97, 71.80, 71.63 (peaks overlapped), 71.36 (peaks overlapped), 71.26, 71.17, 70.8, 70.2, 66.6, 62.13 (peaks overlapped), 62.10, 61.94, 61.89 (peaks overlapped), 61.77, 59.19 (peaks overlapped), 59.01, 58.8 (peaks overlapped), 58.4, 58.12, 57.93, 57.83, 57.78, 57.63, 47.2, 21.5.
HR-MS (ESI)**:** *m/z* = 841.86033 $[M+2Na]^{2+}$ (calcd. 841.85933 for $C_{83}H_{115}NO_{32}Na_2$).

2.4 Synthesis of inc

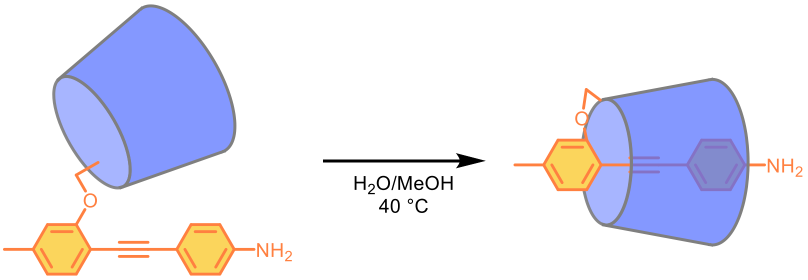

uninc **inc**

Scheme S5. Synthesis of **inc**.

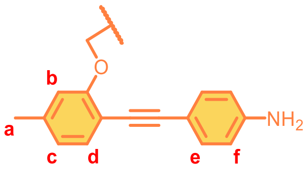

inc. To a solution of **uninc** (20.0 mg, 14.1 μmol) in methanol (2 mL), H_2O (2 mL) was added dropwise at 40 °C. The solution was stirred for 1.5 h at 40 °C. After the mixture cooled to room temperature, the solution was evaporated in vacuum. This compound was used in the dethreading experiments without further purification.
^{1}H NMR (500 MHz, D_2O/CD_3OD (1:1)) δ 7.75 (d, *J* = 8.2 Hz, 2H, H_e), 7.25 (d, *J* = 7.7 Hz, 1H, H_d), 7.13-7.04 (m, 2H, H_b/H_c), 6.82 (d, *J* = 8.1 Hz, 2H, H_f), 5.15 (d, *J* = 2.1 Hz, 1H, H_{CD-1}), 5.12 (d, *J* = 2.8 Hz, 1H, H_{CD-1}), 5.10-5.05 (m, 2H, H_{CD-1}), 5.02-4.99 (m, 2H, H_{CD-1}), 4.84 (t, *J* = 9.1 Hz, 1H, H_{CD-5}), 4.36 (d, *J* = 11.0 Hz, 1H, H_{CD-6}), 4.28-4.20 (m, 3H, H_{CD-5} and/or H_{CD-6}), 4.16 (d, *J* = 9.9 Hz, 1H, H_{CD-5} or H_{CD-6}), 4.08 (d, *J* = 9.6 Hz, 1H, H_{CD-5} or H_{CD-6}), 4.03 (d, *J* = 9.2 Hz, 2H, H_{CD-5} and/or H_{CD-6}), 3.96 (d, *J* = 9.8 Hz, 2H, H_{CD-5} and/or H_{CD-6}), 3.85 (d, *J* = 10.0 Hz, 2H, H_{CD-5} and/or H_{CD-6}), 3.79-3.00 (m, 75H, NH, H_{CD}), 2.78 (d, *J* = 10.7 Hz, 1H, H_{CD-6}), 2.39 (s, 3H, H_a).
^{13}C NMR (126 MHz, D_2O/CD_3OD (1:1)) δ 162.3, 149.7, 142.2, 134.4, 133.5, 125.4, 123.4, 115.8, 114.9, 112.0, 100.86, 100.73, 100.63, 100.45, 100.39, 98.8, 95.6, 85.4, 84.0, 83.4, 82.99, 82.84, 82.79, 82.59, 82.43 (peaks overlapped), 82.31, 82.24, 82.15, 82.12, 82.08 (peaks overlapped), 82.03 (peaks overlapped), 81.8, 81.6, 76.9, 73.5, 72.8, 72.44, 72.39 (peaks overlapped), 72.35, 72.09, 72.00, 71.91 (peaks overlapped), 71.6, 71.2, 62.35, 62.32 (peaks overlapped), 62.22, 62.0, 61.8, 59.46, 59.37, 59.14, 59.00, 58.93, 58.7, 58.44, 58.28, 58.24, 58.19, 58.01, 21.3.

2.5 Synthesis of uninc-^{t}Bu

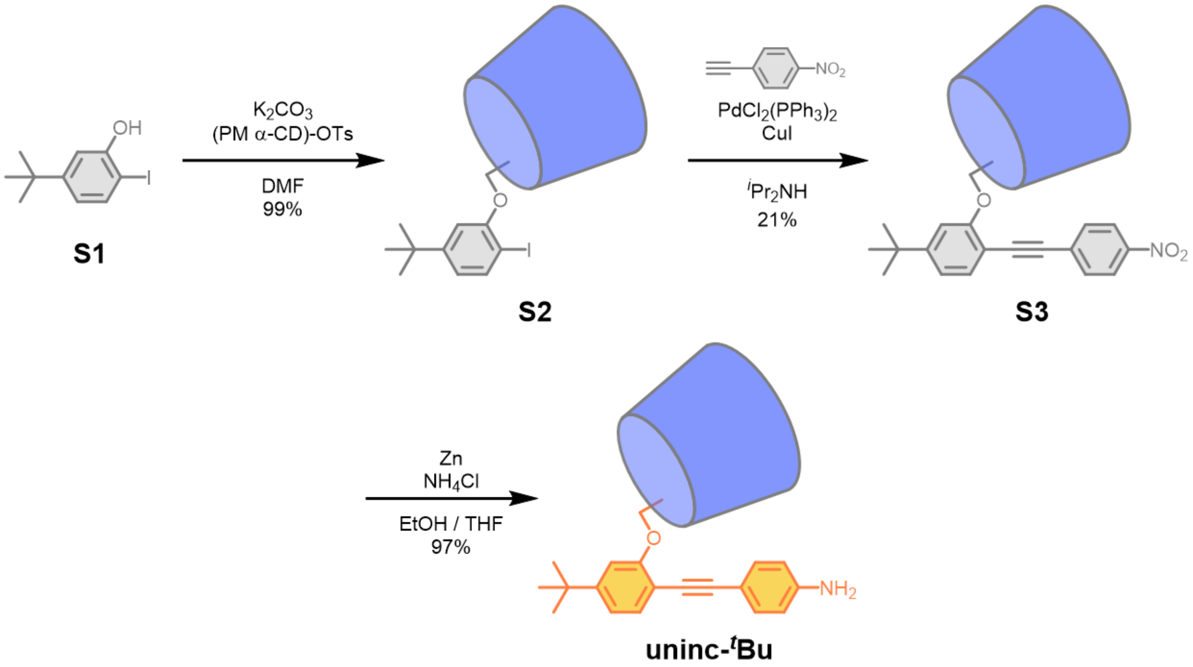

Scheme S6. Synthesis of **uninc-^{t}Bu**.

S2. A mixture of **S1** (242 mg, 876 μmol, 1.20 eq.), dry 6-*O*-monotosyl PM α-CD (1.00 g, 732 μmol), and dry K_2CO_3 (328 mg, 2.37 mmol, 3.24 eq.) in dehydrated DMF (10 mL) was stirred for 6 h at 70 °C. After the mixture cooled to room temperature, the mixture was extracted with ethyl acetate. The organic layers were combined, washed with brine, dried over $MgSO_4$, filtered, and concentrated under reduced pressure. The residue was purified by column chromatography (toluene/50%→100% EtOAc, then EtOAc/0%→15% MeOH) to give **S2** as a white solid (1.07 g, 728 μmol, 99%).

^{1}H NMR (500 MHz, $CDCl_3$) δ 7.66 (d, *J* = 8.3 Hz, 1H, H_{Ar}), 6.87 (d, *J* = 2.1 Hz, 1H, H_{Ar}), 6.81 (dd, *J* = 8.3, 2.1 Hz, 1H, H_{Ar}), 5.11-5.00 (m, 6H, $H_{CD\text{-}1}$), 4.42-2.94 (m, 87H, H_{CD}), 1.31 (s, 9H, H_{tBu}).

^{13}C NMR (126 MHz, $CDCl_3$) δ 157.5, 153.9, 138.5, 120.7, 110.9, 100.54, 100.41, 100.35, 100.28, 100.11, 99.94, 83.5, 83.2, 82.9, 82.61, 82.44 (peaks overlapped), 82.37 (peaks overlapped), 82.32, 82.29, 82.25, 82.14, 82.11, 81.52, 81.40, 81.38, 81.29 (peaks overlapped), 71.88, 71.78, 71.75, 71.56 (peaks overlapped), 71.49, 71.47, 71.37, 71.22, 71.20, 70.51, 70.37, 62.10, 61.99, 61.98 (peaks overlapped), 61.94, 59.25, 59.22, 59.19 (peaks overlapped), 58.8, 57.96 (peaks overlapped), 57.90 (peaks overlapped), 35.0, 31.4.

HR-MS (ESI)**:** *m/z* = 1491.56233 $[M+Na]^{+}$ (calcd. 1491.56276 for $C_{63}H_{105}IO_{30}Na$).

S3. A mixture of **S2** (1.06 g, 721 μmol), 1-ethynyl-4-nitrobenzene (159 mg, 1.08 mmol, 1.50 eq.), $PdCl_2(PPh_3)_2$ (50.6 mg, 72.1 μmol, 10 mol%), and CuI (5.5 mg, 28.9 μmol, 4 mol%) in degassed $^{i}Pr_2NH$ (10 mL) was stirred overnight at 40 °C, and then the solvent was removed in vacuum. The residue was purified by preparative GPC with chloroform as eluent to yield **S3** as a yellow solid (229mg, 154 μmol, 21%).

^{1}H NMR (500 MHz, $CDCl_3$) δ 8.20-8.17 (m, 2H, H_{Ar}), 7.78-7.75 (m, 2H, H_{Ar}), 7.41 (d, *J* = 8.1 Hz, 1H, H_{Ar}), 6.98 (dd, *J* = 8.1, 1.6 Hz, 1H, H_{Ar}), 6.92 (d, *J* = 1.5 Hz, 1H, H_{Ar}), 5.16 (d, *J* = 3.3 Hz, 1H, $H_{CD\text{-}1}$), 5.08-5.04 (m, 4H, $H_{CD\text{-}1}$), 5.01 (d, *J* = 3.3 Hz, 1H, $H_{CD\text{-}1}$), 4.68 (dd, *J* = 10.1, 2.2 Hz, 1H, H_{CD}), 4.36 (t, *J* = 9.2 Hz, 1H, H_{CD}), 4.28 (d, *J* = 9.9 Hz, 1H, H_{CD}), 4.03 (d, *J* = 9.3 Hz, 1H, H_{CD}), 3.92-3.02 (m, 83H, H_{CD}), 1.33 (s, 9H, H_{tBu}).

^{13}C NMR (126 MHz, $CDCl_3$) δ 159.6, 154.8, 146.8, 133.4, 133.2, 132.3, 131.0, 123.7, 123.4, 118.2, 109.3, 100.7, 100.34 (peaks overlapped), 100.28, 100.25, 99.8, 92.5, 91.5, 82.86, 82.77, 82.74 (peaks overlapped), 82.60, 82.42, 82.37, 82.34 (peaks overlapped), 82.31, 82.21, 81.7, 81.41, 81.32 (peaks overlapped), 72.1, 71.88, 71.70 (peaks overlapped), 71.56, 71.49, 71.47, 71.40 (peaks overlapped), 71.30, 70.9, 67.4, 62.06, 61.99, 61.96, 61.94, 61.86 (peaks overlapped), 59.31, 59.24, 59.20, 59.17, 59.01, 58.5, 58.06 (peaks overlapped), 57.99, 57.95, 57.6, 35.4, 31.3.

HR-MS (ESI)**:** *m/z* = 1510.68259 $[M+Na]^+$ (calcd. 1510.68249 for $C_{71}H_{109}NO_{32}Na$).

uninc-^{t}Bu. To a solution of **S3** (200 mg, 134 μmol) in degassed EtOH (10 mL) and THF (10 mL), Zn powder (436 mg, 6.67 mmol, 49.6 eq.) and NH_4Cl (488 mg, 9.12 mmol, 67.9 eq.) was added and stirred for 2 h at room temperature. After the reaction ended, the mixture was filtered and extracted with $CHCl_3$. The organic layers were combined, washed with brine, dried over $MgSO_4$, filtered, and concentrated under reduced pressure. The residue was purified by preparative GPC with chloroform as eluent to yield **uninc-^{t}Bu** as a pale yellow solid (190 mg, 130 μmol, 97%).

^{1}H NMR (500 MHz, THF-d_8) δ 7.32-7.29 (m, 2H, H_{Ar}), 7.23 (d, *J* = 8.0 Hz, 1H, H_{Ar}), 6.98 (d, *J* = 1.7 Hz, 1H, H_{Ar}), 6.89 (dd, *J* = 8.0, 1.7 Hz, 1H, H_{Ar}), 6.54-6.51 (m, 2H, H_{Ar}), 5.12 (d, *J* = 3.4 Hz, 1H, $H_{CD\text{-}1}$), 5.02 (d, *J* = 3.4 Hz, 1H, $H_{CD\text{-}1}$), 4.98-4.96 (m, 3H, H_{CD}), 4.93-4.90 (m, 2H, H_{CD}), 4.75 (s, 2H, NH_2), 4.32 (t, *J* = 9.2 Hz, 1H, H_{CD}), 4.16 (d, *J* = 10.2 Hz, 1H, H_{CD}), 3.98 (d, *J* = 9.6 Hz, 1H, H_{CD}), 3.87-2.90 (m, 83H, H_{CD}), 1.31 (s, 9H, H_{tBu}).

^{13}C NMR (126 MHz, THF-d_8) δ 159.9, 152.6, 149.6, 133.6, 132.3, 117.9, 114.5, 112.35, 112.16, 110.4, 101.2, 100.7, 100.39, 100.37, 100.29, 99.6, 95.1, 84.08, 83.89, 83.74, 83.70, 83.67, 83.64, 83.55, 83.45, 83.36 (peaks overlapped), 83.30 (peaks overlapped), 82.59, 82.51, 82.47 (peaks overlapped), 82.45, 82.39, 73.4, 72.9, 72.67 (peaks overlapped), 72.60, 72.26, 72.13 (peaks overlapped), 72.09, 71.96, 71.6, 68.1, 62.04, 61.88 (peaks overlapped), 61.79, 61.77, 61.73, 58.87, 58.82, 58.79, 58.73, 58.66, 58.5, 58.2, 57.94, 57.85, 57.82, 57.5, 35.6, 31.6.

HR-MS (ESI)**:** *m/z* = 1480.70817 $[M+Na]^+$ (calcd. 1480.70831 for $C_{71}H_{111}NO_{30}Na$).

2.6 Synthesis of uninc-^{t}Bu-Fmoc

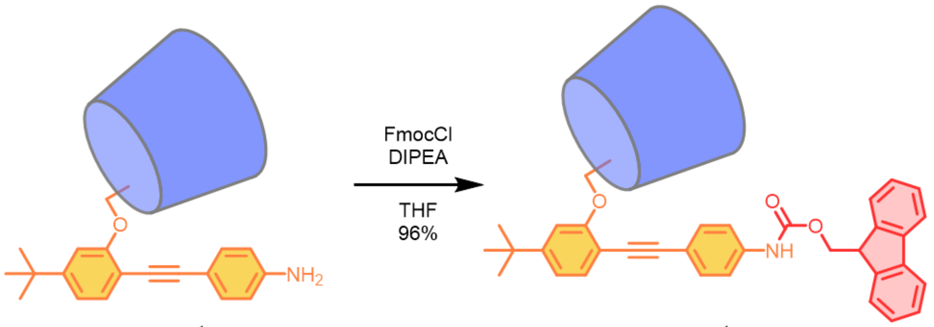

Scheme S7. Synthesis of **uninc-^{t}Bu-Fmoc**.

uninc-^{t}Bu-Fmoc. A solution of **uninc-^{t}Bu** (50 mg, 34 μmol) in dry THF (4 mL) was stirred for 2 h at 60 °C. To the solution, FmocCl (17.7 mg, 68.4 μmol, 2.00 eq.) and DIPEA (13 μL, 76 μmol, 2.2 eq.) were added and the mixture was stirred overnight at 60 °C, and then the solvent was removed in vacuum. The residue was purified by preparative GPC with chloroform as eluent to yield **uninc-^{t}Bu-Fmoc** as a white solid (55.5 mg, 33.0 μmol, 96%).

^{1}H NMR (500 MHz, $CDCl_3$) δ 7.79 (d, J = 7.5 Hz, 2H, H_{Ar}), 7.62 (d, J = 7.4 Hz, 2H, H_{Ar}), 7.54 (d, J = 8.4 Hz, 2H, H_{Ar}), 7.44-7.40 (m, 2H, H_{Ar}), 7.38-7.31 (m, 5H, H_{Ar}), 6.94 (d, J = 8.1 Hz, 1H, H_{Ar}), 6.89 (d, J = 0.8 Hz, 1H, H_{Ar}), 6.70 (s, 1H, NH), 5.20 (d, J = 3.2 Hz, 1H, $H_{CD\text{-}1}$), 5.07-5.03 (m, 4H, $H_{CD\text{-}1}$), 5.01 (d, J = 3.2 Hz, 1H, $H_{CD\text{-}1}$), 4.65 (dd, J = 10.1, 1.8 Hz, 1H, H_{CD}), 4.56 (d, J = 6.5 Hz, 2H, $H_{Fmoc\text{-}CH2}$), 4.34 (t, J = 9.2 Hz, 1H, H_{CD}), 4.31-4.25 (m, 2H, H_{CD}/$H_{Fmoc\text{-}CH}$), 3.99 (d, J = 9.3 Hz, 1H, H_{CD}), 3.85-3.02 (m, 83H), 1.31 (s, 9H, H_{tBu}).

^{13}C NMR (126 MHz, $CDCl_3$) δ 159.1, 153.1, 143.8, 141.5, 137.5, 132.74, 132.63, 128.0, 127.3, 125.0, 120.2, 119.0, 118.4, 118.0, 110.6, 109.4, 100.8, 100.33, 100.27, 100.25, 100.24, 99.6, 92.7, 86.0, 82.80, 82.71 (peaks overlapped), 82.40 (peaks overlapped), 82.38, 82.34 (peaks overlapped), 82.30, 82.11, 81.7, 81.35 (peaks overlapped), 81.32 (peaks overlapped), 81.29, 81.26, 72.1, 71.82, 71.66, 71.64, 71.61, 71.46 (peaks overlapped), 71.37 (peaks overlapped), 71.1, 67.5, 67.0, 62.00, 61.96, 61.95, 61.93, 61.92, 61.83, 59.28, 59.22, 59.18 (peaks overlapped), 59.06, 58.4, 58.03, 58.00, 57.97, 57.94, 57.4, 47.2, 35.2, 31.4.

HR-MS (ESI)**:** m/z = 1702.77623 $[M+Na]^+$ (calcd. 1702.77639 for $C_{86}H_{121}NO_{32}Na$).

2.7 Synthesis of inc-^{t}Bu-Fmoc

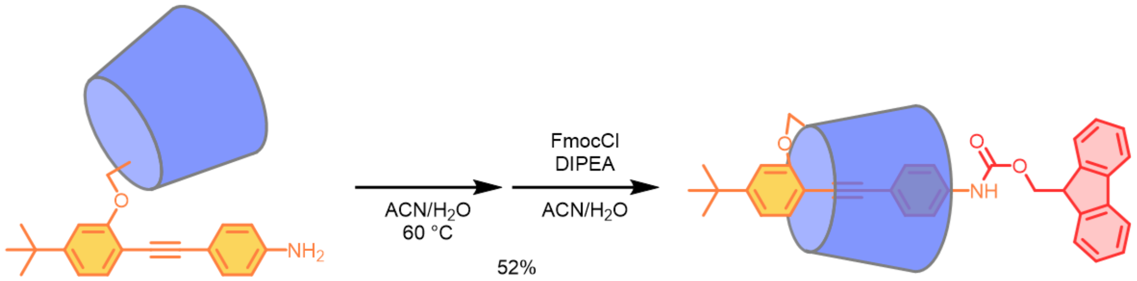

unincv-^{t}Bu **inc-^{t}Bu-Fmoc**

Scheme S8. Synthesis of **inc-^{t}Bu-Fmoc**.

inc-^{t}Bu-Fmoc. A solution of **uninc-^{t}Bu** (50.0 mg, 34.3 μmol) in ACN (5 mL) and H_2O (5 mL) was stirred for 2 h at 60 °C. To the solution, FmocCl (25.7 mg, 99.3 μmol, 2.90 eq.) and DIPEA (19 μL, 0.11 mmol, 3.3 eq.) were added and the mixture was stirred overnight at 60 °C, and then the solvent was removed in vacuum. The residue was purified by preparative GPC with chloroform as eluent to yield **inc-^{t}Bu-Fmoc** as a yellow solid (30.1 mg, 17.9 μmol, 52%).

^{1}H NMR (500 MHz, $CDCl_3$) δ 8.00 (d, J = 8.6 Hz, 2H, H_{Ar}), 7.79 (d, J = 7.5 Hz, 2H, H_{Ar}), 7.61-7.55 (m, 4H, H_{Ar}), 7.45-7.39 (m, 2H, H_{Ar}), 7.35-7.31 (m, 3H, H_{Ar}), 7.16-7.10 (m, 2H, H_{Ar}), 6.75 (s, 1H, NH), 5.08 (d, J = 3.1 Hz, 1H, $H_{CD\text{-}1}$), 5.05 (d, J = 3.4 Hz, 1H, $H_{CD\text{-}1}$), 5.00 (d, J = 3.0 Hz, 1H, $H_{CD\text{-}1}$), 4.96 (d, J = 3.0 Hz, 2H, $H_{CD\text{-}1}$), 4.95 (d, J = 3.3 Hz, 1H, $H_{CD\text{-}1}$), 4.86 (td, J = 9.1, 1.9 Hz, 1H, H_{CD}), 4.54 (d, J = 6.4 Hz, 2H, $H_{Fmoc\text{-}CH2}$), 4.34 (dd, J = 11.4, 2.3 Hz, 2H, H_{CD}), 4.26-4.18 (m, 3H, H_{CD}/$H_{Fmoc\text{-}CH}$), 4.12-4.06 (m, 2H, H_{CD}), 4.01-3.92 (m, 4H, H_{CD}), 3.81-3.03 (m, 75H), 2.69-2.67 (m, 1H, H_{CD}), 1.33 (s, 9H, H_{tBu}).

^{13}C NMR (126 MHz, $CDCl_3$) δ 161.9, 154.3, 152.4, 143.8, 141.5, 138.7, 133.5, 132.8, 127.95, 127.93, 127.24, 127.22, 124.9, 120.4, 120.2, 119.3, 117.5, 117.3, 113.9, 100.9, 100.5, 100.18, 100.14, 100.12, 98.1, 93.4, 86.4, 84.0, 82.68 (peaks overlapped), 82.61 (peaks overlapped), 82.53, 82.36, 82.30, 82.19, 82.12 (peaks overlapped), 81.78, 81.59, 81.39, 81.23 (peaks overlapped), 81.18, 76.4, 72.3, 71.9, 71.73, 71.67 (peaks overlapped), 71.45, 71.34, 71.31, 71.14, 70.98, 70.2, 66.6, 62.06 (peaks overlapped), 61.89, 61.85, 61.67, 59.21, 59.19, 59.10, 58.70, 58.61, 58.36, 58.20, 57.90, 57.85, 57.80, 57.67, 47.2, 35.0, 31.3.

HR-MS (ESI): m/z = 1702.77616 $[M+Na]^+$ (calcd. 1702.77639 for $C_{86}H_{121}NO_{32}Na$).

2.8 Net unidirectional rotation in a one-pot manner

To a solution of **uninc** (14.3 mg, 10.1 μmol) in THF (1 mL), DIPEA (3.74 μL, 22.0 μmol, 2.2 eq.) and FmocCl (5.2 mg, 20.1 μmol, 2.0 eq.) in a pressure vessel were added and stirred for 1 h at 60 °C. After the mixture cooled to room temperature, a 50 μL aliquot was taken, and NMR measurements ($CDCl_3$) were conducted after removing the solvent (**uninc-Fmoc**). Next, MeOH (5.7 mL) and H_2O (8.55 mL) were added to the mixture and stirred for 15 min at 100 °C. After cooling, a 50 μL aliquot was taken, and NMR measurements ($CDCl_3$) were conducted with similar procedures (**inc-Fmoc**). Subsequently, the solvent was removed, followed by the addition of THF (810 μL) and piperidine (90 μL). The mixture was stirred for 1 h at 25 °C, a 50 μL aliquot was taken, and NMR measurements ($CDCl_3$) were conducted with similar procedures

(**uninc**). The solvent was removed in vacuum, toluene (1 mL) and sat. NH_4Cl aq. (1 mL) were added, and the mixture was extracted with toluene (2 × 1 mL) in the pressure vessel to remove residual piperidine. Removal of solvent affords crude mixture of **uninc**, which was reintroduced into the initial step. The amounts of reagents were adjusted based on the amount of molecular motor, which decreases due to the sampling for NMR measurements (Table S1).

Table S1. Reagent amount used in direct sequential net unidirectional rotation experiments.

	**uninc → uninc-Fmoc**			**uninc-Fmoc → inc-Fmoc**		**inc-Fmoc → uninc**	
	DIPEA / µL	FmocCl / mg	THF / mL	MeOH / mL	H_2O / mL	THF / µL	piperidine / µL
1st cycle	3.74	5.2	1	5.7	8.55	810	90
2nd cycle	3.18	4.4	0.85	4.8	7.2	675	75
3rd cycle	2.62	3.6	0.7	3.9	5.85	540	60
4th cycle	2.06	2.9	0.55	3	4.5	405	45

2.9 Net unidirectional rotation in the reverse direction

To a solution of **uninc** (8.7 mg, 6.1 µmol) in ACN (4 mL) and H_2O (4 mL), DIPEA (3.37 µL, 19.8 µmol, 3.2 eq.) and FmocCl (4.6 mg, 17.8 µmol, 2.9 eq.) were added, and the mixture was stirred for 1 h at 60 °C. After the mixture cooled to room temperature, the solvent was evaporated and THF (6 mL) was added. A 50 µL aliquot was taken, and NMR measurements ($CDCl_3$) were conducted after removing the solvent (**inc-Fmoc**). The mixture containing **inc-Fmoc** was stirred for 15 min at 100 °C. After cooling, a 50 µL aliquot was taken, and NMR measurements ($CDCl_3$) were conducted with similar procedures (**uninc-Fmoc**). To the mixture, piperidine (222 µL) was added and the mixture was stirred for 30 min at 25 °C. Again, a 50 µL aliquot was taken, and NMR measurements ($CDCl_3$) were conducted with similar procedures (**uninc**).

3. NMR measurements

3.1 ROESY spectra of uninc-Fmoc and inc-Fmoc

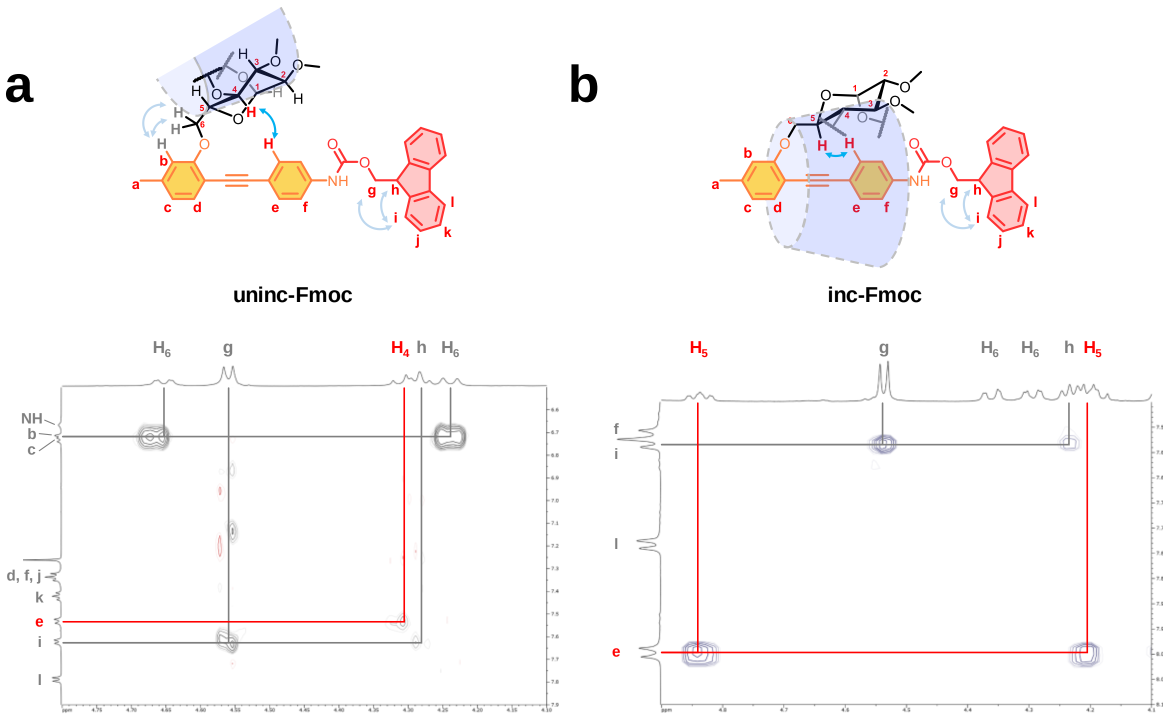

Figure S1. Partial ROESY NMR spectra of (a) **uninc-Fmoc** (500 MHz, $CDCl_3$, 304 K) and (b) **inc-Fmoc** (500 MHz, $CDCl_3$, 302 K).

3.2 Threading reaction of uninc-Fmoc

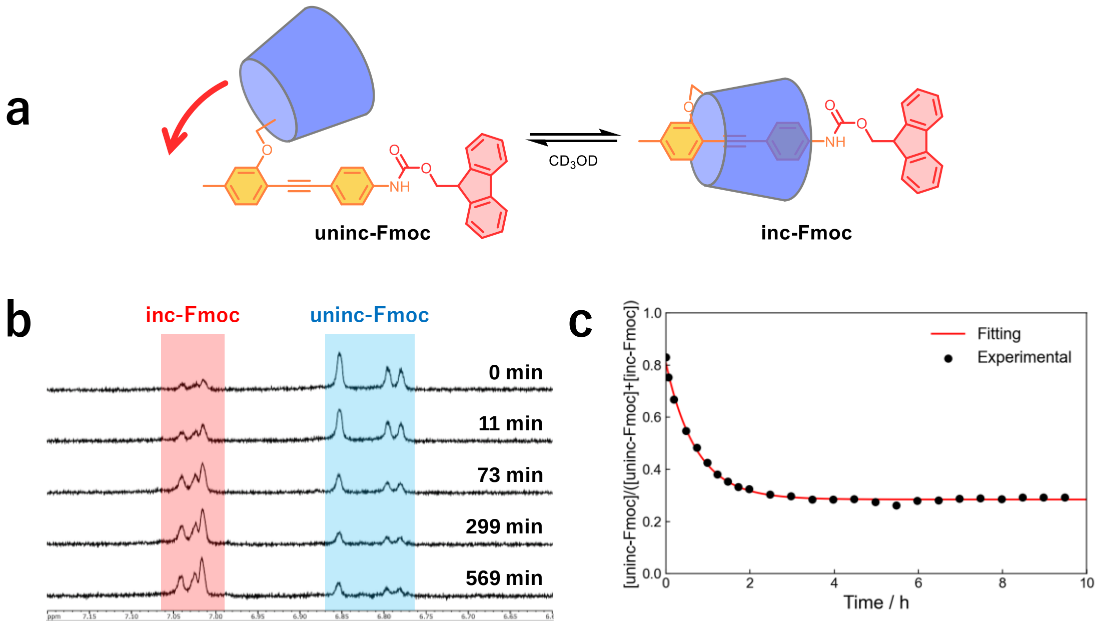

Figure S2. (a) Scheme of threading reaction of **uninc-Fmoc**, (b) change in ^{1}H NMR spectra during the threading reaction of **uninc-Fmoc** in CD_3OD (500 MHz, 333 K), and (c) change in [**uninc-Fmoc**]/([**uninc-Fmoc**]+[**inc-Fmoc**]).

The threading of **uninc-Fmoc** in CD_3OD was monitored using NMR measurements (Figure S2). Table S3 shows the reaction progress calculated from the NMR integration values.

Table S2. Reaction progress of threading reaction of **inc-Fmoc** in CD_3OD at 333 K.

Time / s	[**uninc-Fmoc**]/([**uninc-Fmoc**]+[**inc-Fmoc**])	Time / s	[**uninc-Fmoc**]/([**uninc-Fmoc**]+[**inc-Fmoc**])
0	0.830	14305	0.284
228	0.753	16105	0.286
674	0.668	17910	0.275
1719	0.547	19698	0.261
2638	0.484	21507	0.280
3525	0.426	23313	0.281
4399	0.381	25109	0.288
5299	0.354	26905	0.289
6200	0.332	28706	0.287
7100	0.324	30515	0.293
8906	0.304	32304	0.292
10700	0.297	34133	0.292
12504	0.284		

These data were fitted using lmfit,[6] assuming first-order reaction kinetics for the deprotection based on the model equation below:

$$\frac{[\text{uninc-Fmoc}]}{[\text{uninc-Fmoc}]+[\text{inc-Fmoc}]} = \frac{k_{\text{out}}}{k_{\text{in}}+k_{\text{out}}} + \left(r_0 - \frac{k_{\text{out}}}{k_{\text{in}}+k_{\text{out}}}\right) \times e^{-(k_{\text{in}}+k_{\text{out}})t}$$

The results are presented in Table S3. The threading rate k_{in} and dethreading rate k_{out} are 2.8×10^{-4} s^{-1} and 1.1×10^{-4} s^{-1}, respectively.

Table S3. Fitting results of dethreading process of **inc-Fmoc**.

Fit Statistics

fitting method	leastsq
# function evals	21
# data points	25
# variables	3
chi-square	0.00220376
reduced chi-square	0.00010017
Akaike info crit.	-227.411678
Bayesian info crit.	-223.75505

Variables

name	value	standard error	relative error	initial value	min	max	vary
k_in	0.000275	8.1149E-06	-2.95%	0.0001	-inf	inf	TRUE
k_out	0.00010925	3.9965E-06	-3.66%	0.0001	-inf	inf	TRUE
r0	8.07E-01	6.90E-03	-0.85%	8.00E-01	-inf	inf	TRUE

Correlations

k_in	k_out	0.9443
k_in	r0	0.5289
k_out	r0	0.4661

3.3 Heating experiment of uninc-^{t}Bu-Fmoc in CD_3OD

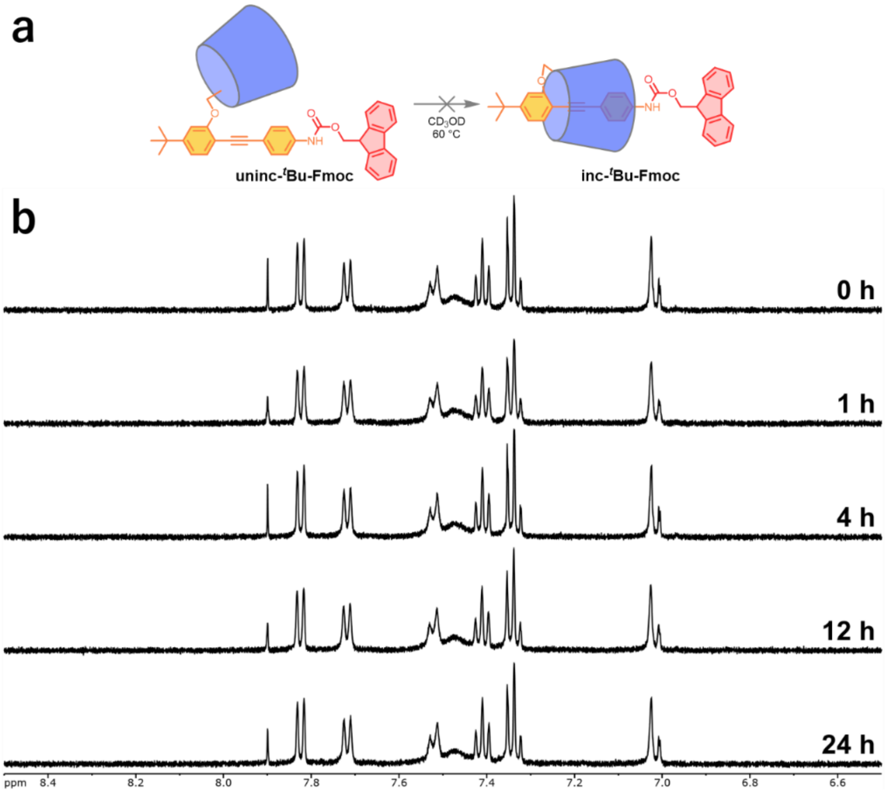

Figure S3. (a) Scheme of dethreading reaction of **uninc-^{t}Bu-Fmoc** and (b) ^{1}H NMR spectra of **uninc-^{t}Bu-Fmoc** under heating at 333 K in CD_3OD (500 MHz, 298 K).

3.4 Dethreading reaction of inc

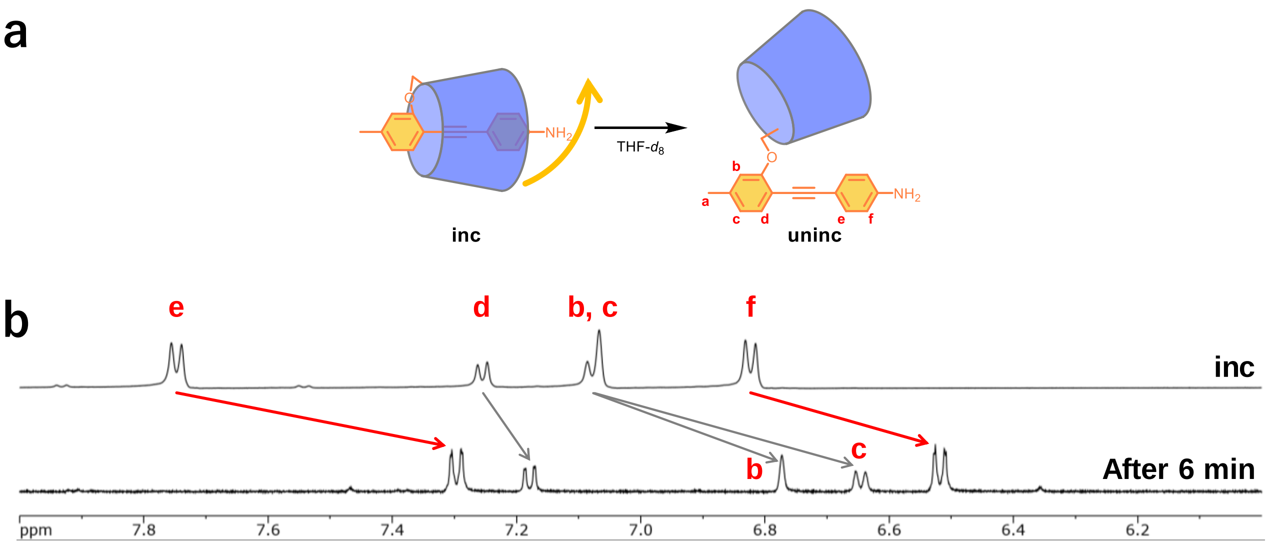

Figure S4. (a) Scheme of dethreading reaction of **inc** and (b) ^{1}H NMR spectra before and after the dethreading of **inc** (500 MHz; solvents: CD_3OD/D_2O (**inc**), THF-d_8 (After 6 min); temperature: 301 K (**inc**), 303 K (After 6 min)).

The NMR measurements reveal that the dethreading reaction of **inc** proceeds within 6 minutes (Figure S4). The reaction rate constant for achieving 99% conversion within 6 minutes is given by the following equation:

$$-\frac{\ln(1-0.99)}{6\times 60\ [\mathrm{s}]} = 1.3\times 10^{-2}[\mathrm{s}^{-1}]$$

It is expected that the actual reaction rate is greater than this calculated value.

3.5 Fmoc deprotection of inc-Fmoc

Similar to the dethreading reaction of **inc**, the NMR measurements reveal that the Fmoc deprotection of **inc-Fmoc** proceeds within 10 minutes (Figure 4B). The reaction rate constant for achieving 99% conversion within 10 minutes is given by the following equation:

$$-\frac{\ln(1-0.99)}{10\times 60\ [\mathrm{s}]} = 7.7\times 10^{-3}[\mathrm{s}^{-1}]$$

It is expected that the actual reaction rate is greater than this calculated value.

3.6 Dethreading reaction of inc-Fmoc

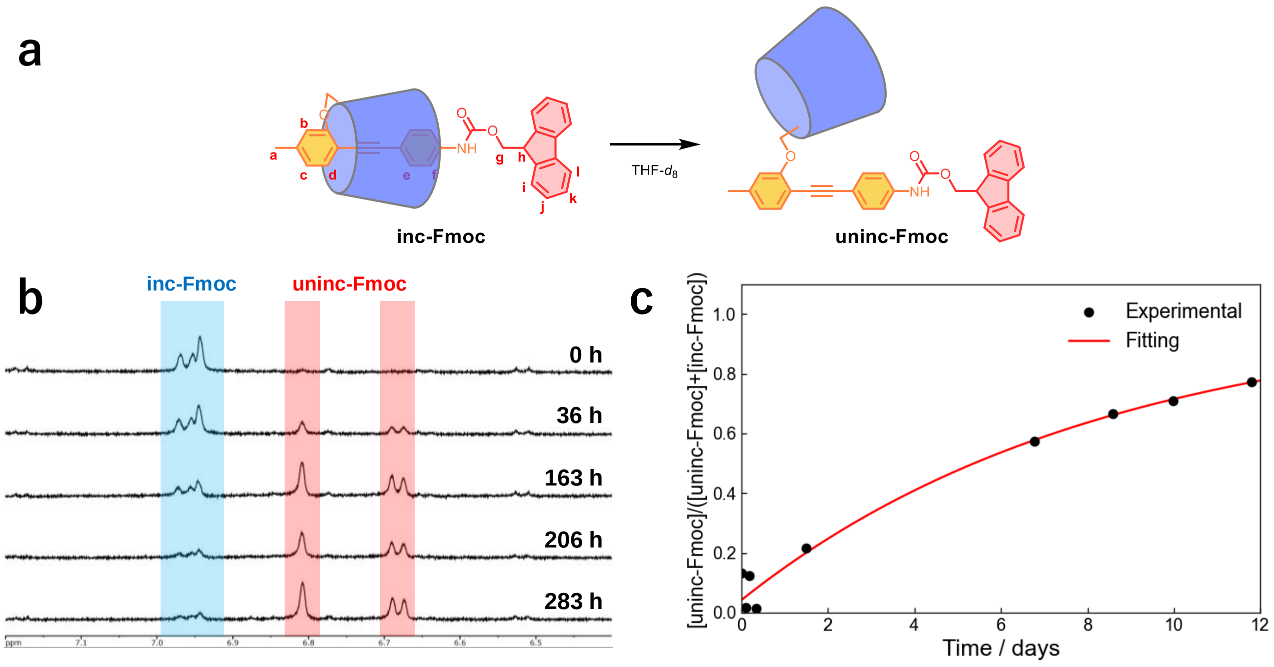

Figure S5. (a) Scheme of dethreading reaction of **inc-Fmoc**, (b) ^{1}H NMR spectral change in the dethreading of **inc-Fmoc** in THF-d_8 (500 MHz, 303 K), and (c) Change in [**uninc-Fmoc**]/([**uninc-Fmoc**]+[**inc-Fmoc**]).

The dethreading of **inc-Fmoc** was successfully monitored using NMR measurements (Figure S5). Table S4 shows the reaction progress calculated from the NMR integration values.

Table S4. Reaction progress of dethreading reaction of **inc-Fmoc**.

Time / s	[**uninc-Fmoc**]/([**uninc-Fmoc**]+[**inc-Fmoc**])
0	0.132
2834	-0.017
8226	0.017
16012	0.123
28624	0.014
128821	0.216
585877	0.574
742801	0.667
861985	0.710
1019045	0.773

These data were fitted using lmfit,[6] assuming first-order reaction kinetics for the deprotection based on the model equation below:

$$\frac{[\text{uninc-Fmoc}]}{[\text{uninc-Fmoc}]+[\text{inc-Fmoc}]} = a \times e^{-kt} + b$$

The results are presented in Table S5. The reaction rate is 1.4×10^{-6} s^{-1} and the calculated half-life of **inc-Fmoc** is 5.8 days.

Table S5. Fitting results of dethreading process of **inc-Fmoc**.

Fit Statistics

fitting method	leastsq
# function evals	49
# data points	10
# variables	3
chi-square	0.02138903
reduced chi-square	0.00305558
Akaike info crit.	-55.4746238
Bayesian info crit.	-54.5668685

Variables

name	value	standard error	relative error	initial value	min	max	vary
a	1.00645299	0.28514766	28.33%	1	-inf	inf	TRUE
b	-0.96430219	0.27773252	28.80%	1	-inf	inf	TRUE
k	1.39E-06	7.66E-07	55.31%	1.00E-05	-inf	inf	TRUE

Correlations

a	b	-0.9962
a	k	-0.9883
b	k	0.9794

3.7 Fmoc protection of uninc

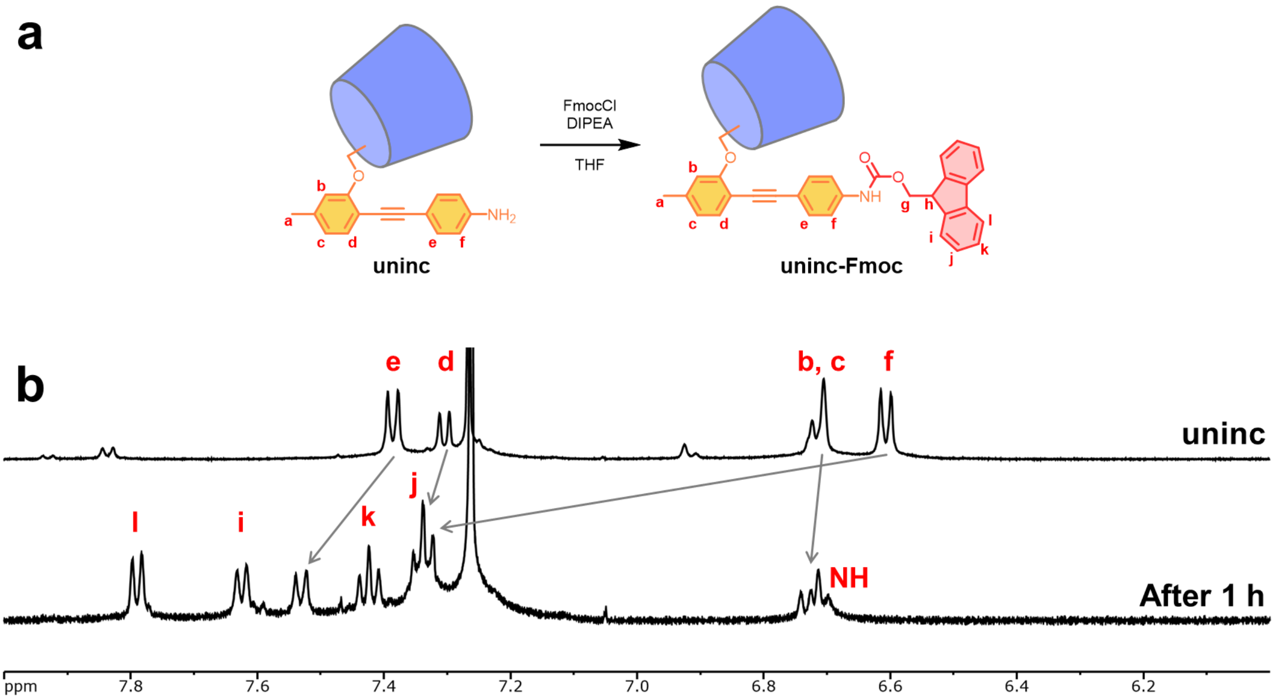

Figure S6. (a) Synthetic scheme of **uninc-Fmoc** and (b) ^{1}H NMR spectra (500 MHz, $CDCl_3$, 300–301 K) before and after Fmoc protection.

3.8 One-pot net unidirectional rotation

The molar ratios of **uninc**/**uninc-Fmoc**/**inc-Fmoc** in the one-pot net unidirectional rotation experiment were determined using ^{1}H NMR measurements. Four signal regions, labeled as Region A through D, were specified, and the normalized integral values of standard samples (Table S6) and the reaction aliquots (Figure S7, Table S7) were measured. It should be noted that, in the case of **uninc**, an equilibrium with **inc** exists in $CDCl_3$. Due to the slow exchange, they are fully separated and observed in the NMR spectrum. For the analysis, the equilibrium mixture of **uninc**/**inc** was used as a standard sample in the calculations.

Table S6. Normalized integral values of standard samples.

	Region			
	A	B	C	D
**uninc**	0.13	1.01		0.86
**uninc-Fmoc**		2	0.71	
**inc-Fmoc**	2			1

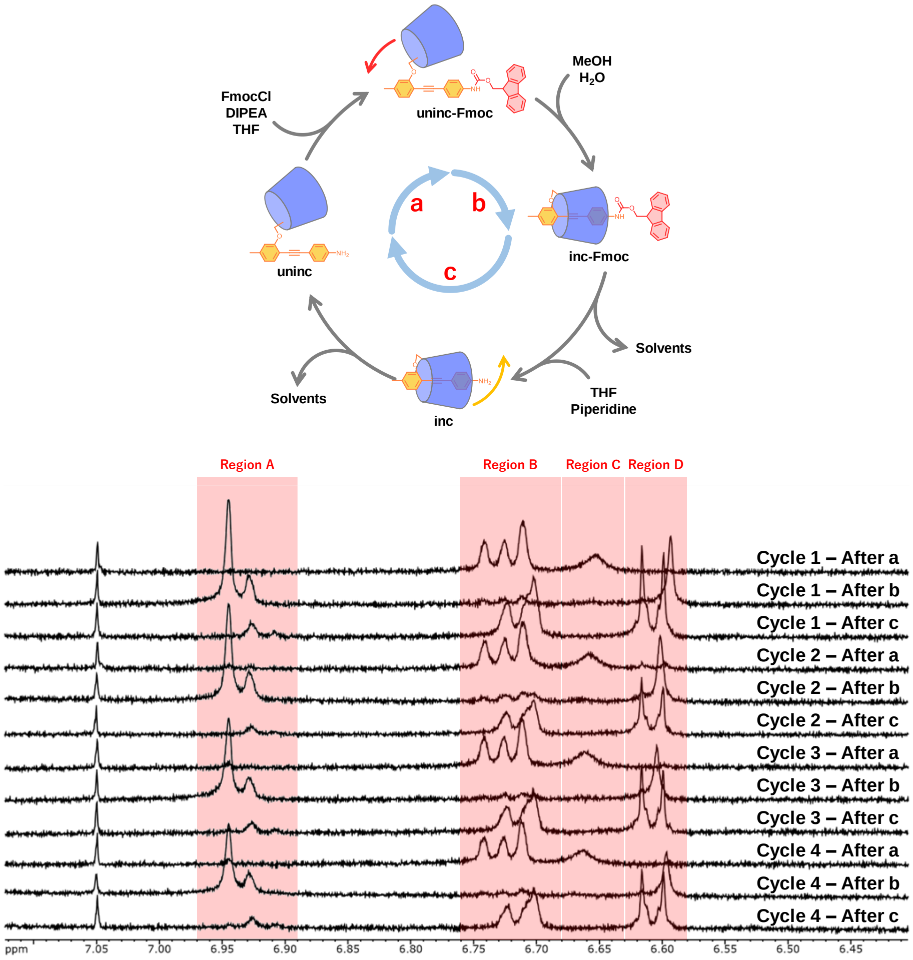

Figure S7. Partial ^{1}H NMR spectra of **uninc**/**uninc-Fmoc**/**inc-Fmoc** under one-pot net unidirectional rotation (500 MHz, $CDCl_3$, 296–297 K)

Table S7. Integral values of ^{1}H NMR spectra of **uninc/uninc-Fmoc/inc-Fmoc** under one-pot net unidirectional rotation.

cycle	state	Region			
		A	B	C	D
1	after a	0.0385	1	0.376	0.0462
1	after b	1	0.186	0.0872	0.505
1	after c	0.174	1	0.0659	0.801
2	after a	0.118	1	0.345	0.123
2	after b	1	0.251	0.0688	0.504
2	after c	0.179	1	0.111	0.765
3	after a	0.0082	1	0.333	0.0223
3	after b	1	0.197	0.0844	0.505
3	after c	0.115	1	0.0624	0.806
4	after a	0.0798	1	0.324	0.0403
4	after b	1	0.239	0.0985	0.405
4	after c	0.381	1	0.119	0.803

By employing lmfit,[6] we calculated [**uninc**], [**uninc-Fmoc**], [**inc-Fmoc**], and their errors to minimize the sum of squared errors as follows.

$$\text{Error} = \begin{bmatrix} \text{Integral of A} \\ \text{Integral of B} \\ \text{Integral of C} \\ \text{Integral of D} \end{bmatrix} - \begin{bmatrix} 0.13 & 0 & 2 \\ 1.01 & 2 & 0 \\ 0 & 0.71 & 0 \\ 0.86 & 0 & 1 \end{bmatrix} \begin{bmatrix} [\textbf{uninc}] \\ [\textbf{uninc-Fmoc}] \\ [\textbf{inc-Fmoc}] \end{bmatrix}$$

Table S8. Fitting results of one-pot net unidirectional rotation process.

Cycle	State	Ratio / %		
		**uninc**	**uninc-Fmoc**	**inc-Fmoc**
1	after a	3.2 ±6.7	**92.9 ±3.9**	3.9 ±2.8
1	after b	-1.0 ±4.3	16.6 ±2.5	**84.4 ±1.8**
1	after c	**90.6 ±3.7**	6.1 ±2.1	3.3 ±1.5
2	after a	11.9 ±3.4	**78.6 ±2**	9.4 ±1.4
2	after b	2.3 ±3.2	18.4 ±1.9	**79.2 ±1.3**
2	after c	**85.8 ±6.9**	9.9 ±4	4.3 ±2.9
3	after a	5.8 ±2.9	**94.0 ±1.7**	0.2 ±1.2
3	after b	-0.4 ±3	16.9 ±1.8	**83.4 ±1.3**
3	after c	**95.5 ±5.1**	4.4 ±2.9	0.2 ±2.1
4	after a	3.0 ±6.2	**90.3 ±3.6**	6.7 ±2.6
4	after b	-18.4 ±5.6	29.6 ±3.2	**88.7 ±2.3**
4	after c	**73.4 ±4.3**	12.4 ±2.5	14.2 ±1.8

3.9 Net unidirectional rotation in the reverse direction

The molar ratios of **uninc/uninc-Fmoc/inc-Fmoc** in the net unidirectional rotation experiment in the reverse direction were also determined using ^{1}H NMR measurements. In this case, the integral values from region A, B, and D were employed, and the normalized integral values of the reaction aliquots (Figure S8, Table S9) were measured.

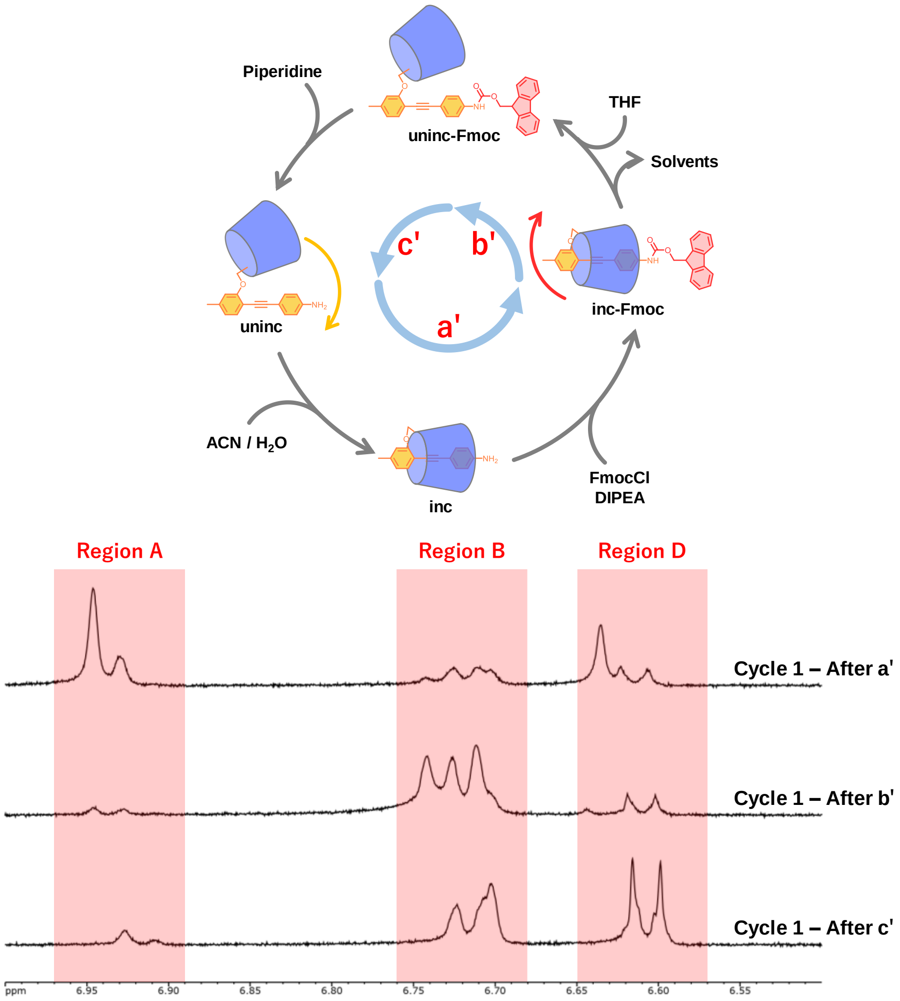

Figure S8. Partial ^{1}H NMR spectra of **uninc/uninc-Fmoc/inc-Fmoc** under net unidirectional rotation in reverse direction (500 MHz, $CDCl_3$, 295–296 K).

Table S9. Integral values of ^{1}H NMR spectra of **uninc/uninc-Fmoc/inc-Fmoc** under one-pot net unidirectional rotation in the reversed direction.

State	Region		
	A	B	D
after a	2.05	1.12	1.37
after b	1	15.1	2.19
after c	1	7.55	6.12

The integral value vector $\boldsymbol{x}$ can be expressed by the following equation:

$$\boldsymbol{x} = \boldsymbol{A}\boldsymbol{c}$$

where:

$$\boldsymbol{x} = \begin{bmatrix} \text{Integral of A} \\ \text{Integral of B} \\ \text{Integral of D} \end{bmatrix} \qquad \text{(integral value)}$$

$$\boldsymbol{A} = \begin{bmatrix} 0.13 & 0 & 2.0 \\ 1.01 & 2.0 & 0 \\ 0.86 & 0 & 1.0 \end{bmatrix} \qquad \text{(Normalized integral values, Table S6)}$$

$$\boldsymbol{c} = \begin{bmatrix} [\textbf{uninc}] \\ [\textbf{uninc-Fmoc}] \\ [\textbf{inc-Fmoc}] \end{bmatrix} \qquad \text{(concentration)}$$

Therefore, the concentration vector c can be calculated using the following equation (Table S10).

$$\boldsymbol{c} = \boldsymbol{A}^{-1}\boldsymbol{x}$$

where:

$$\boldsymbol{A}^{-1} = \begin{bmatrix} 0.63 & 0 & 1.3 \\ 0.32 & 0.50 & -0.64 \\ 0.54 & 0 & -0.082 \end{bmatrix}$$

Table S10. Calculated abundance ratio of **uninc/uninc-Fmoc/inc-Fmoc** under one-pot net unidirectional rotation in the reversed direction.

State	Ratio / %		
	**uninc**	**uninc-Fmoc**	**inc-Fmoc**
after a'	24.5	19.2	**56.3**
after b'	23.7	**72.2**	4.0
after c'	**96.6**	2.8	0.6

3.10 Heating experiment of inc-^{t}Bu-Fmoc in THF

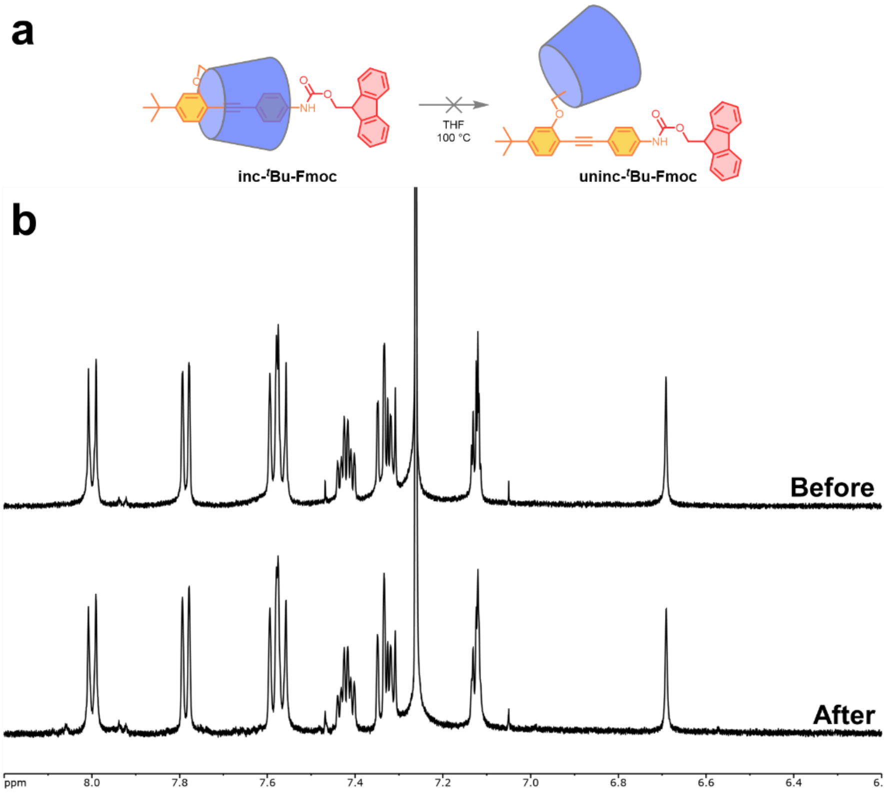

Figure S9. ^{1}H NMR spectra of **inc-^{t}Bu-Fmoc** before and after heating for 15 min at 373 K in THF (500 MHz, $CDCl_3$, 298 K).

4. Compound Data

4.1 NMR spectra of compound 2

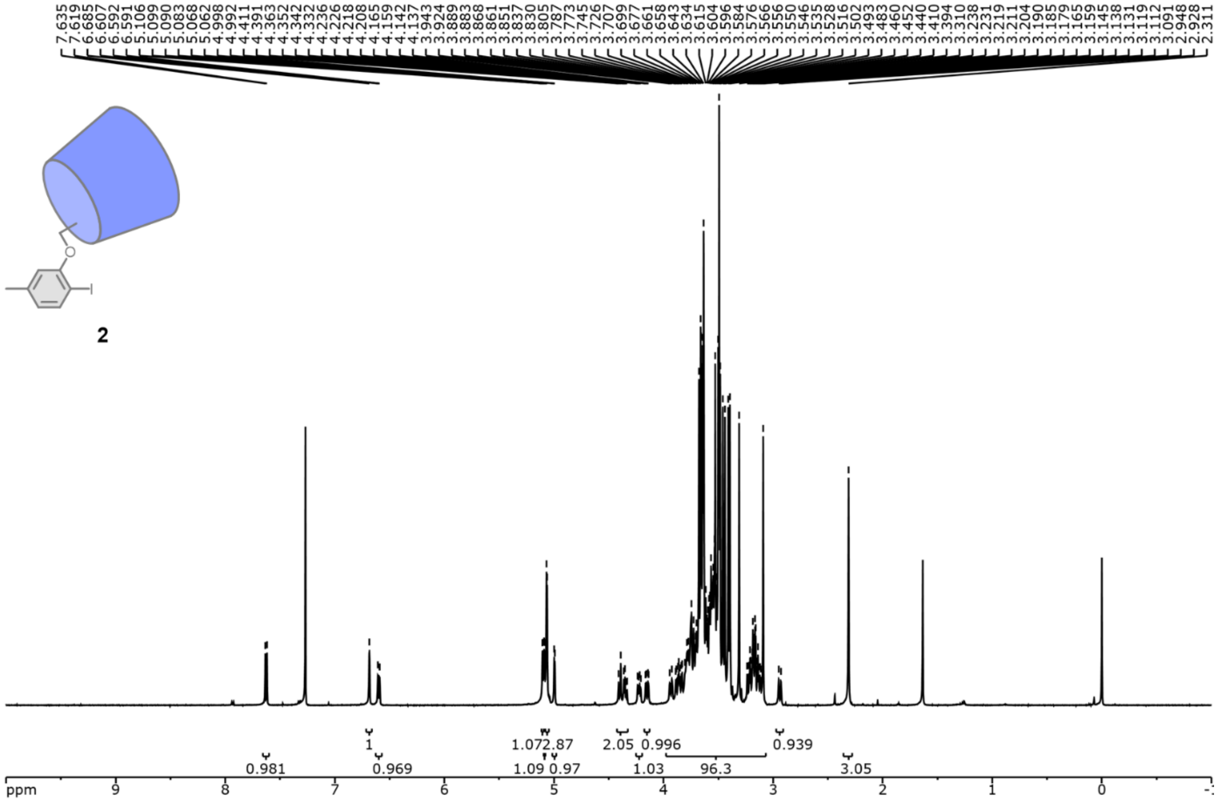

Figure S10. ^{1}H NMR spectrum of **2** (500 MHz, $CDCl_3$, 297 K).

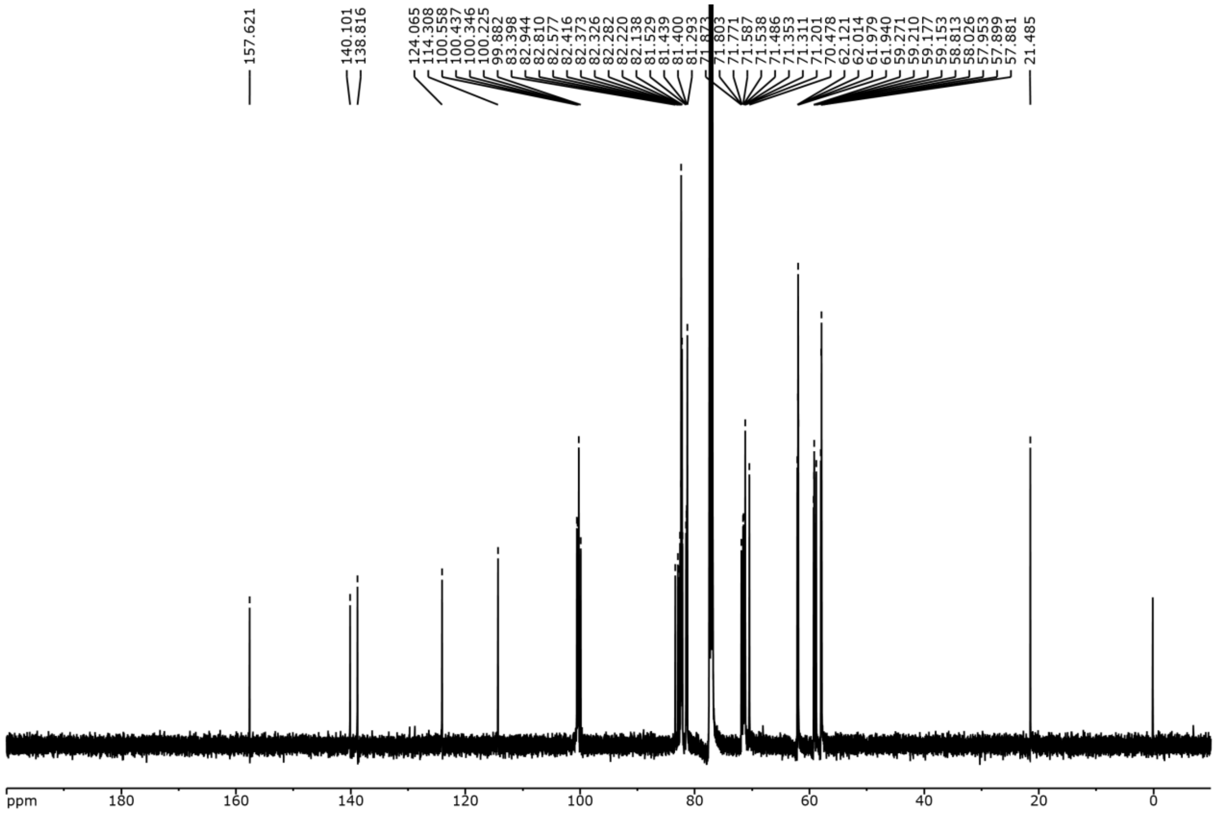

Figure S11. $^{13}C\ \{^{1}H\}$ NMR spectrum of **2** (126 MHz, $CDCl_3$, 298 K).

4.2 NMR spectra of 3

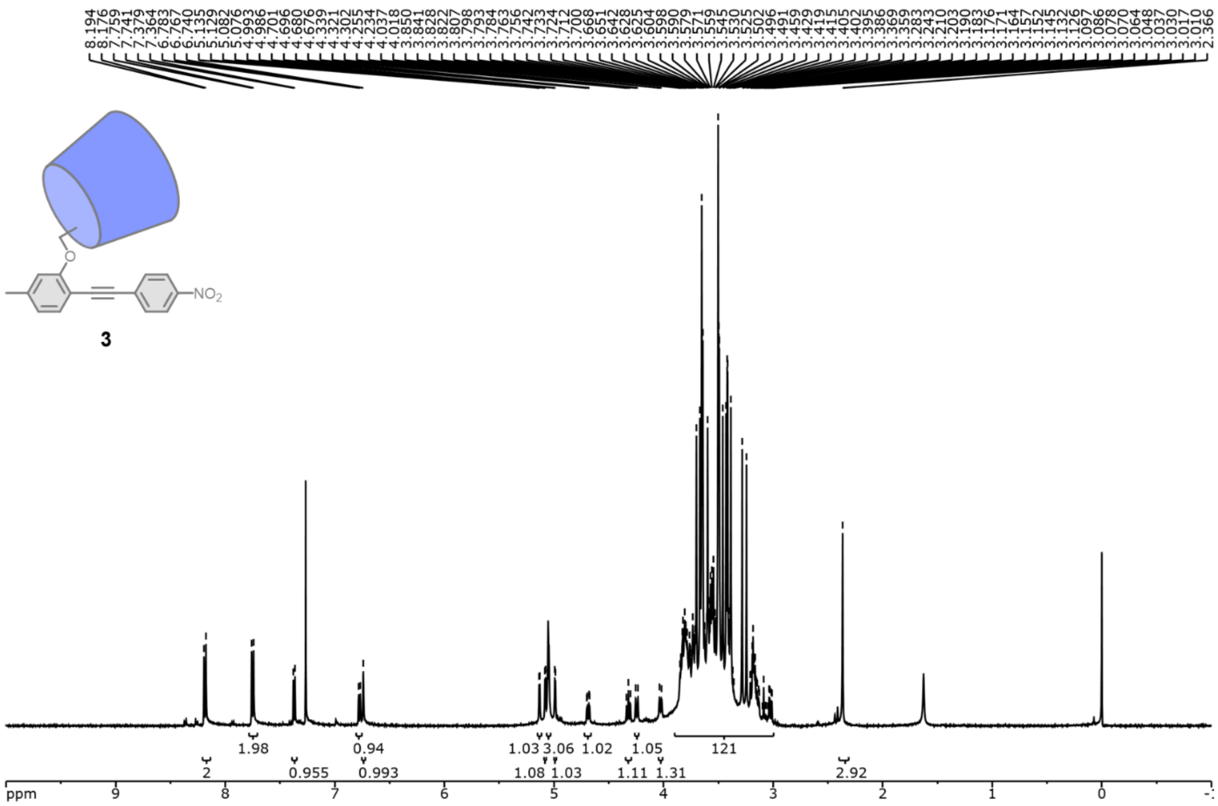

Figure S12. ^{1}H NMR spectrum of **3** (500 MHz, $CDCl_3$, 300 K).

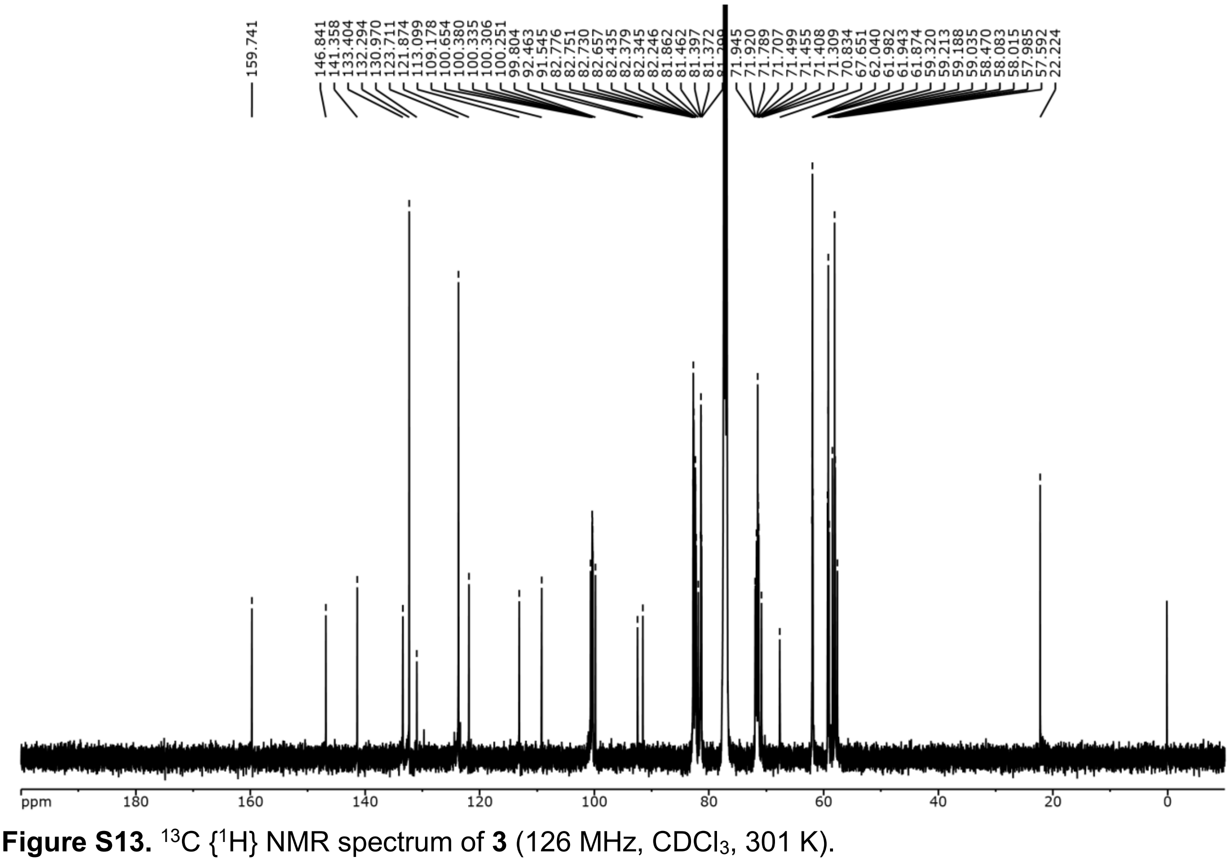

Figure S13. ^{13}C {^{1}H} NMR spectrum of **3** (126 MHz, $CDCl_3$, 301 K).

4.3 NMR spectra of uninc

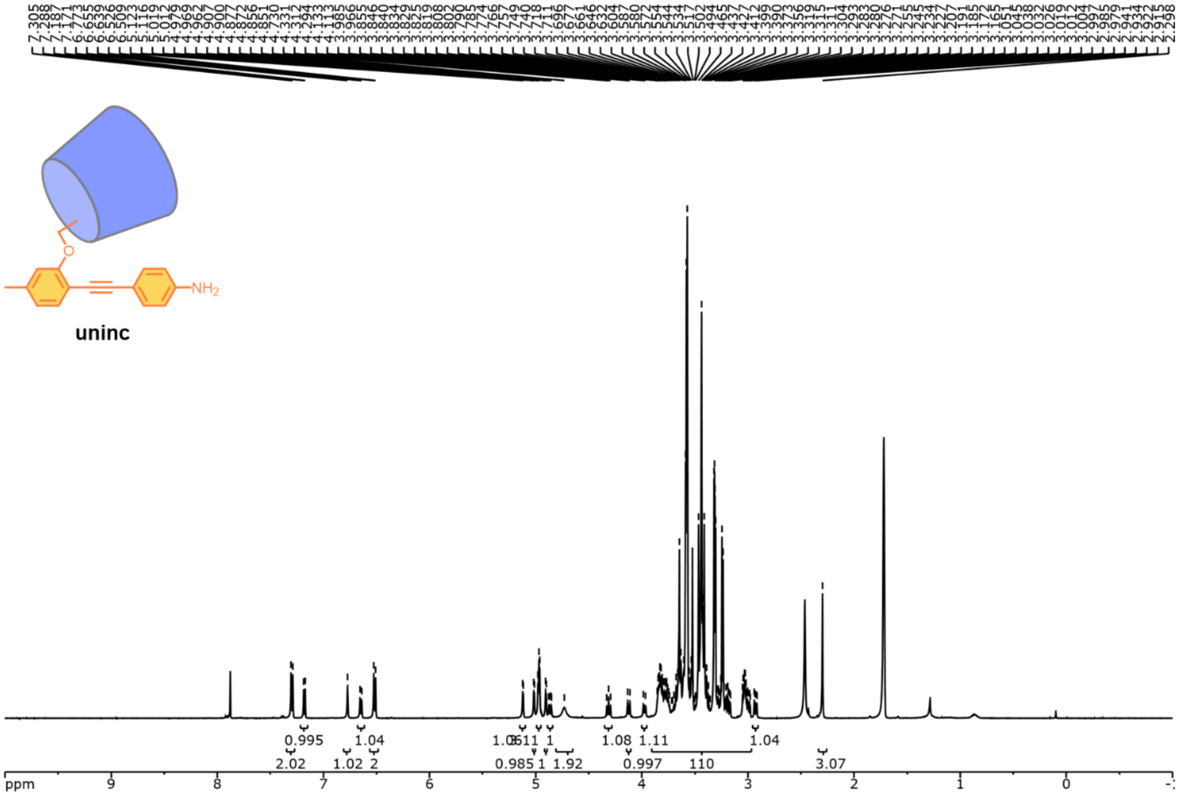

Figure S14. ^{1}H NMR spectrum of **uninc** (500 MHz, THF-d_8, 300 K).

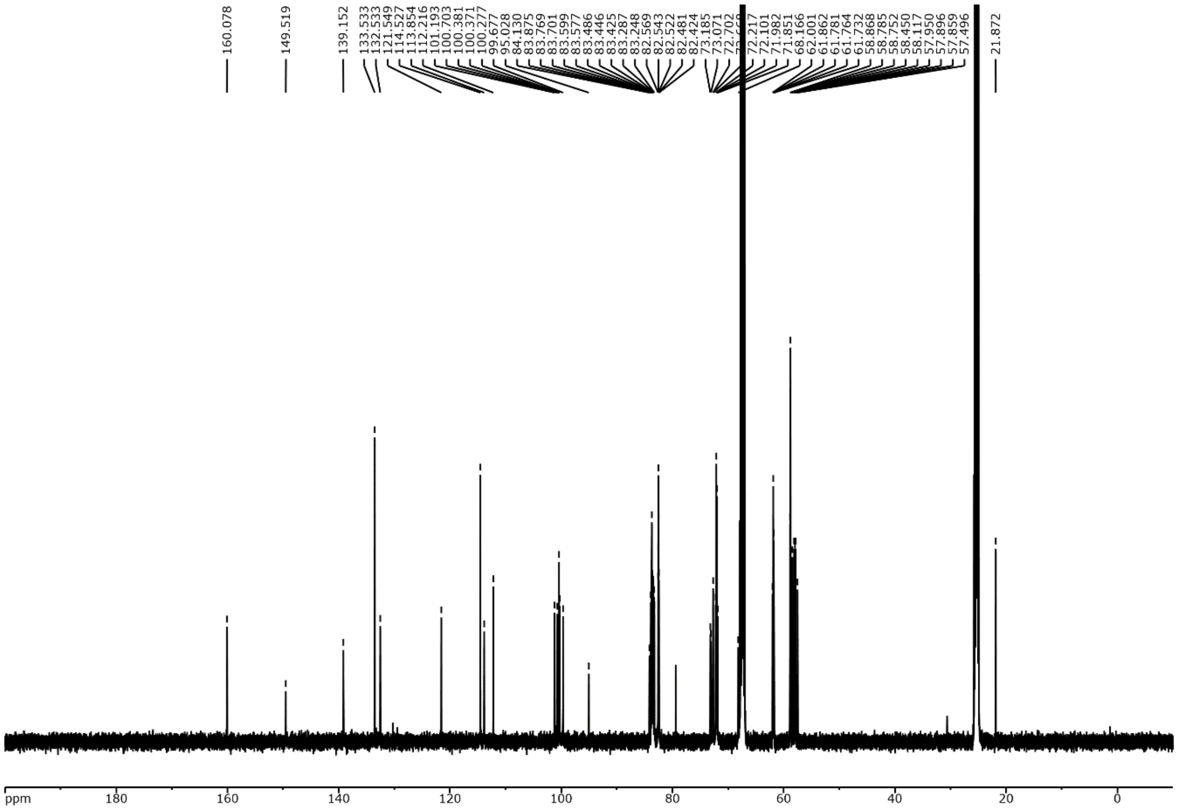

Figure S15. ^{13}C {^{1}H} NMR spectrum of **uninc** (126 MHz, THF-d_8, 301 K).

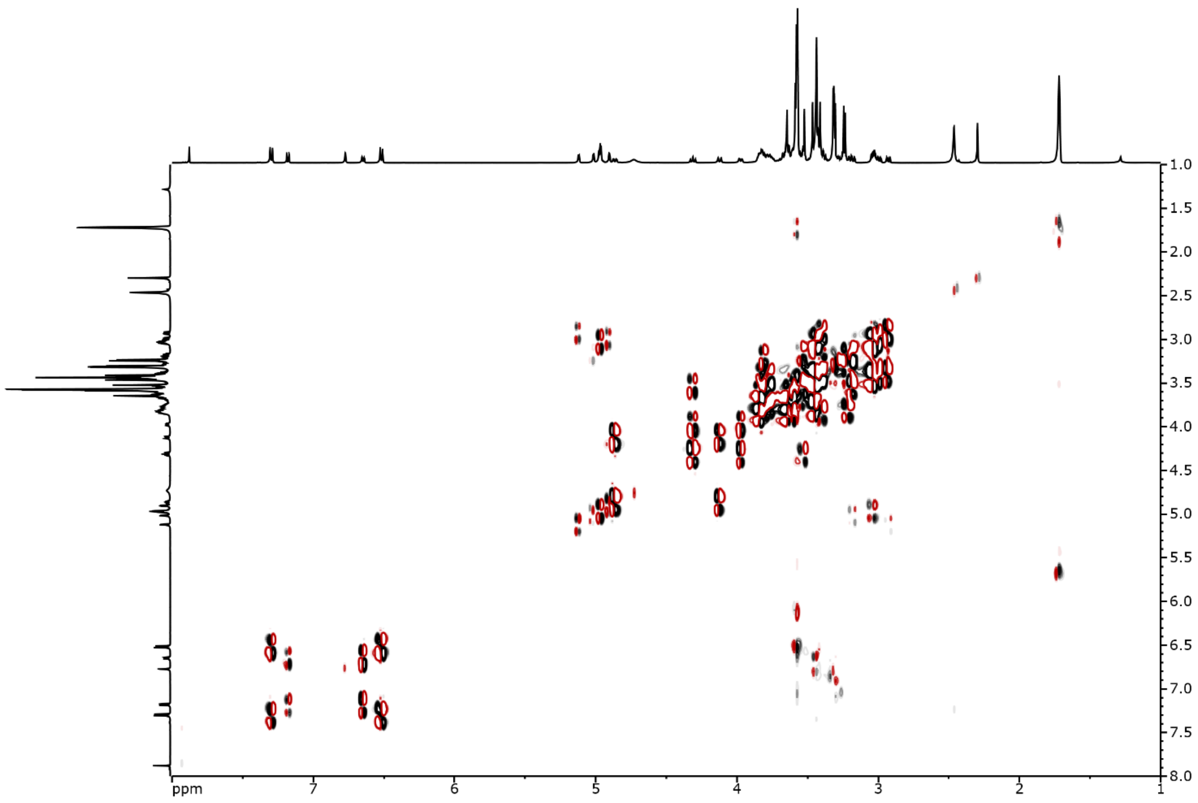

Figure S16. ^{1}H-^{1}H DQF-COSY spectrum of **uninc** (500 MHz, THF-d_8. 300 K).

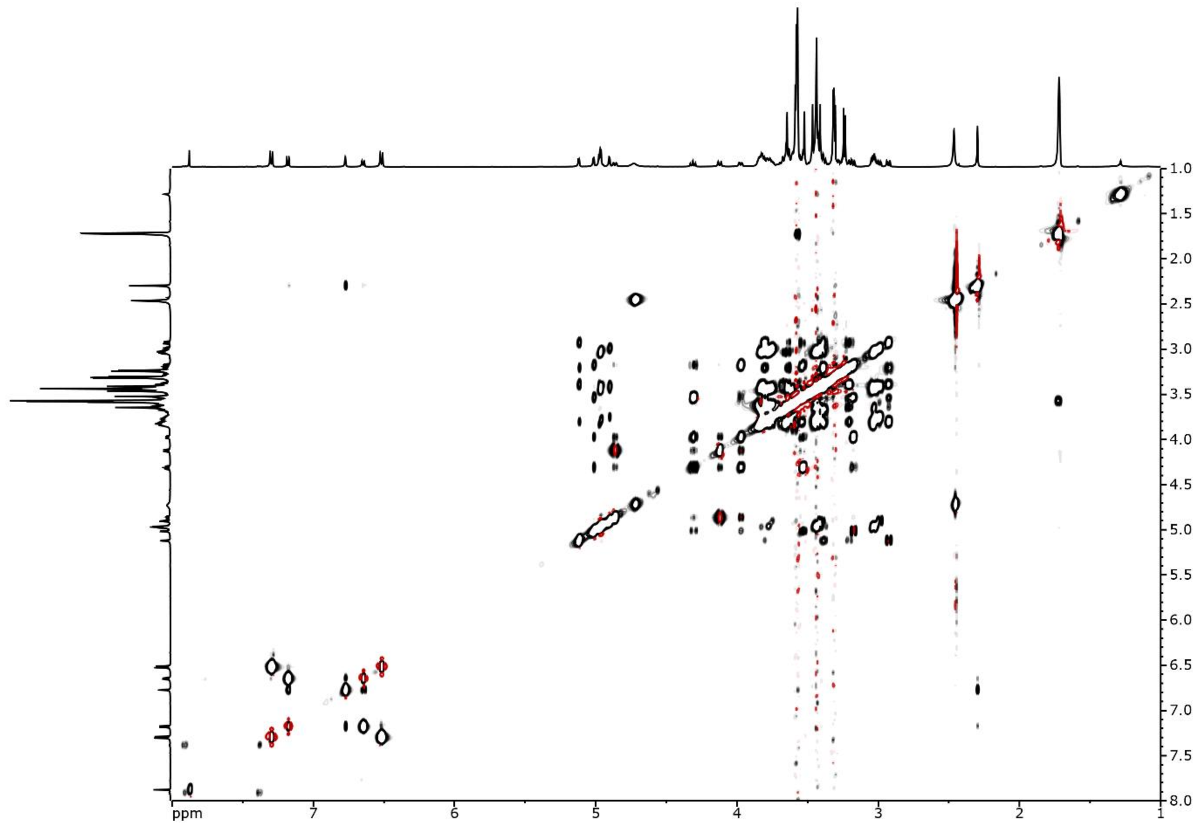

Figure S17. ^{1}H-^{1}H TOCSY spectrum of **uninc** (500 MHz, THF-d_8, 301 K).

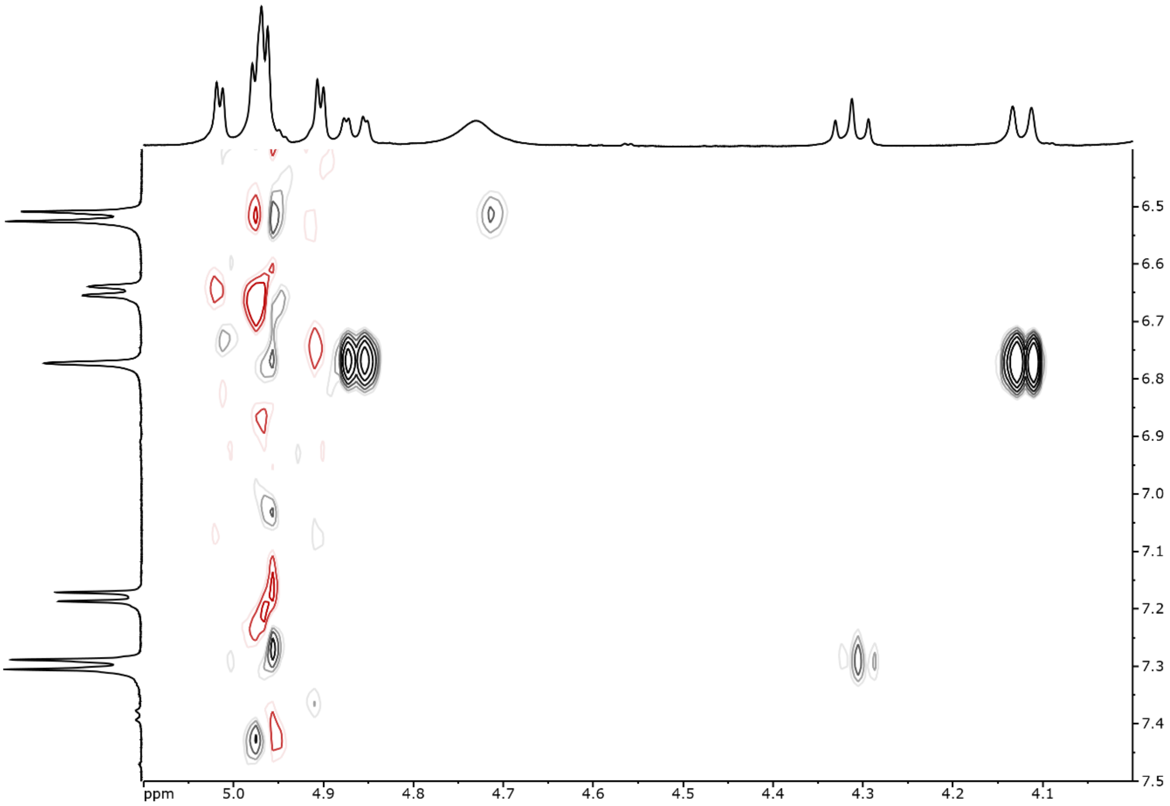

Figure S18. Partial ^{1}H-^{1}H ROESY spectrum of **uninc** (500 MHz, THF-d_8. 302 K).

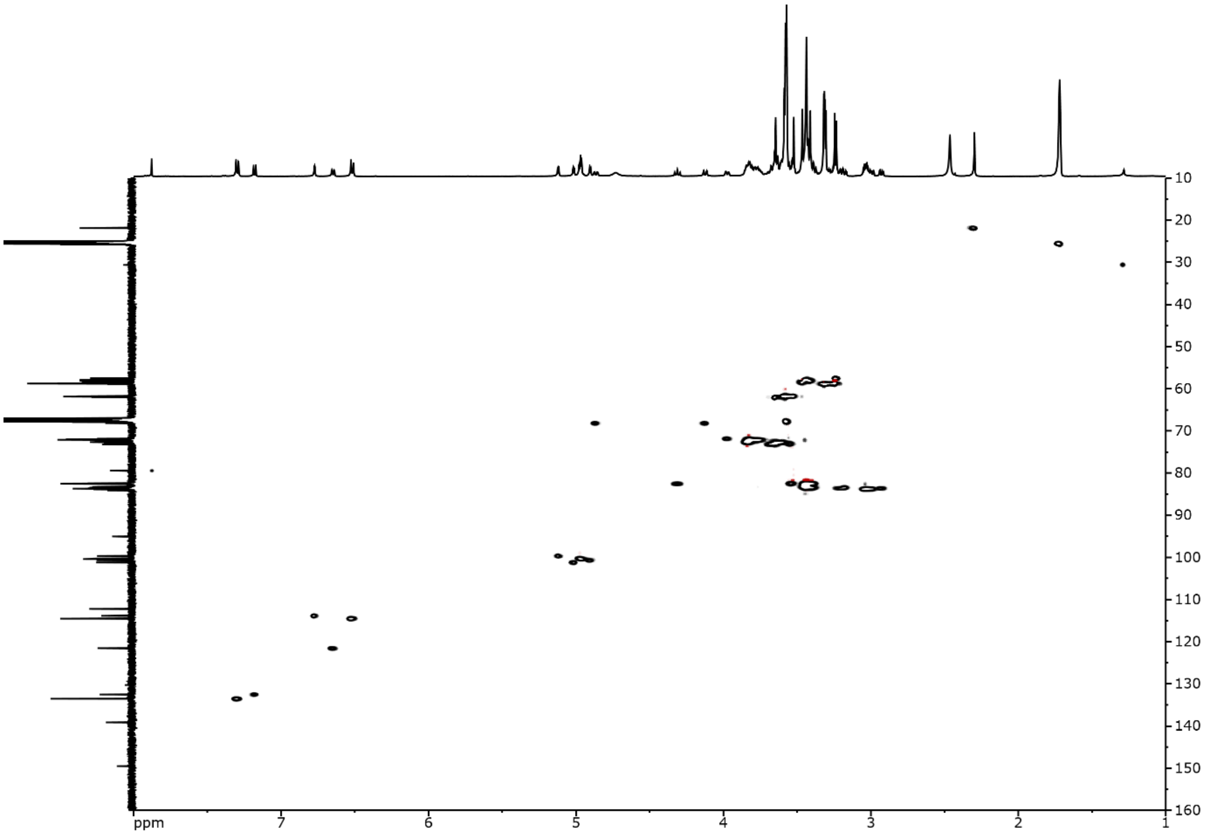

Figure S19. ^{1}H-^{13}C HSQC spectrum of **uninc** (500/126 MHz, THF-d_8, 302 K).

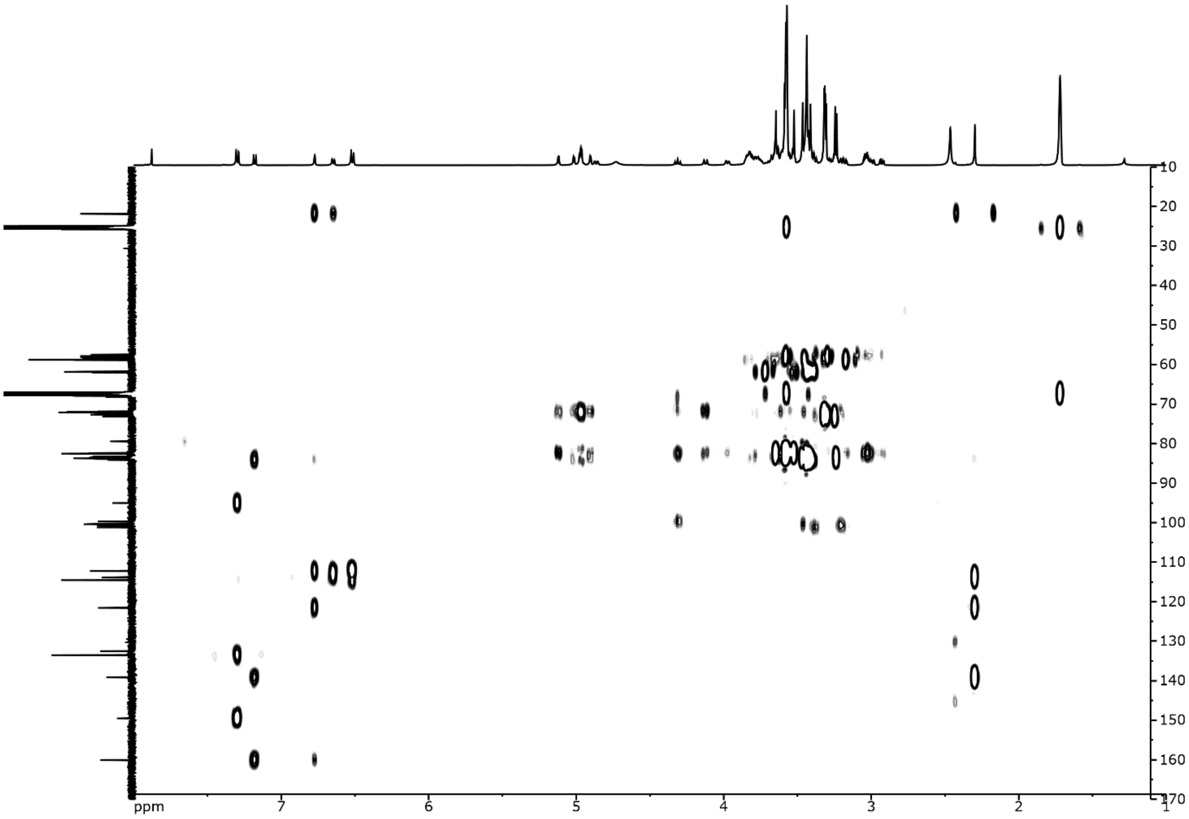

Figure S20. ^{1}H-^{13}C HMBC spectrum of **uninc** (500/126 MHz, THF-d_8, 302 K).

4.4 NMR spectra of uninc-Fmoc

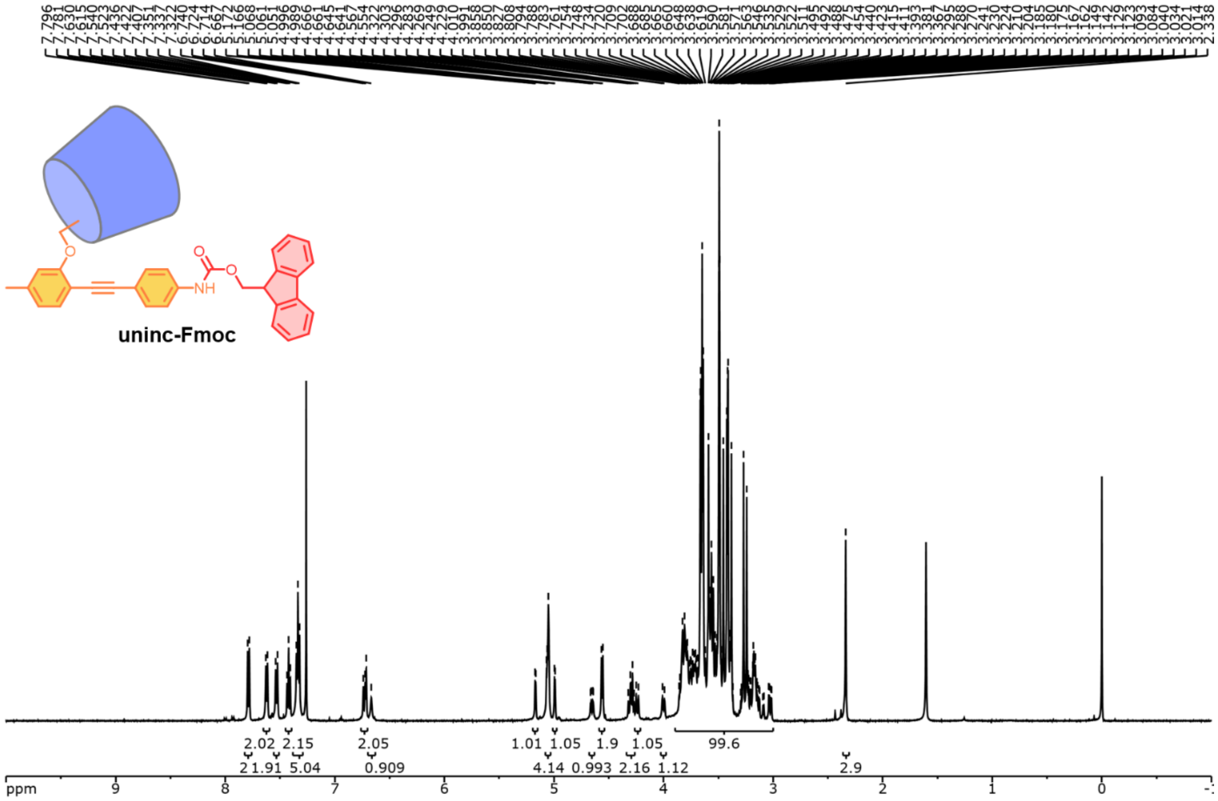

Figure S21. ^{1}H NMR spectrum of **uninc-Fmoc** (500 MHz, $CDCl_3$, 301 K).

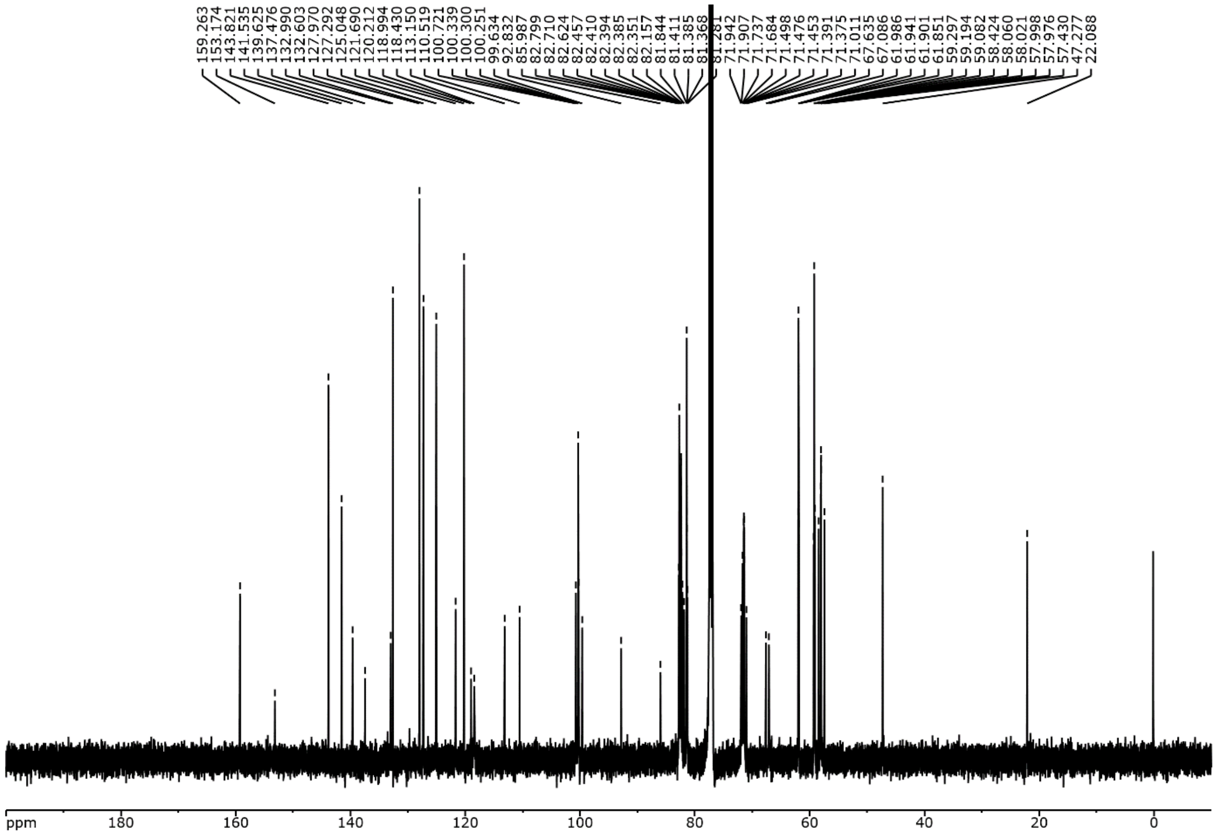

Figure S22. ^{13}C $\{^{1}H\}$ NMR spectrum of **uninc-Fmoc** (126 MHz, $CDCl_3$, 303 K).

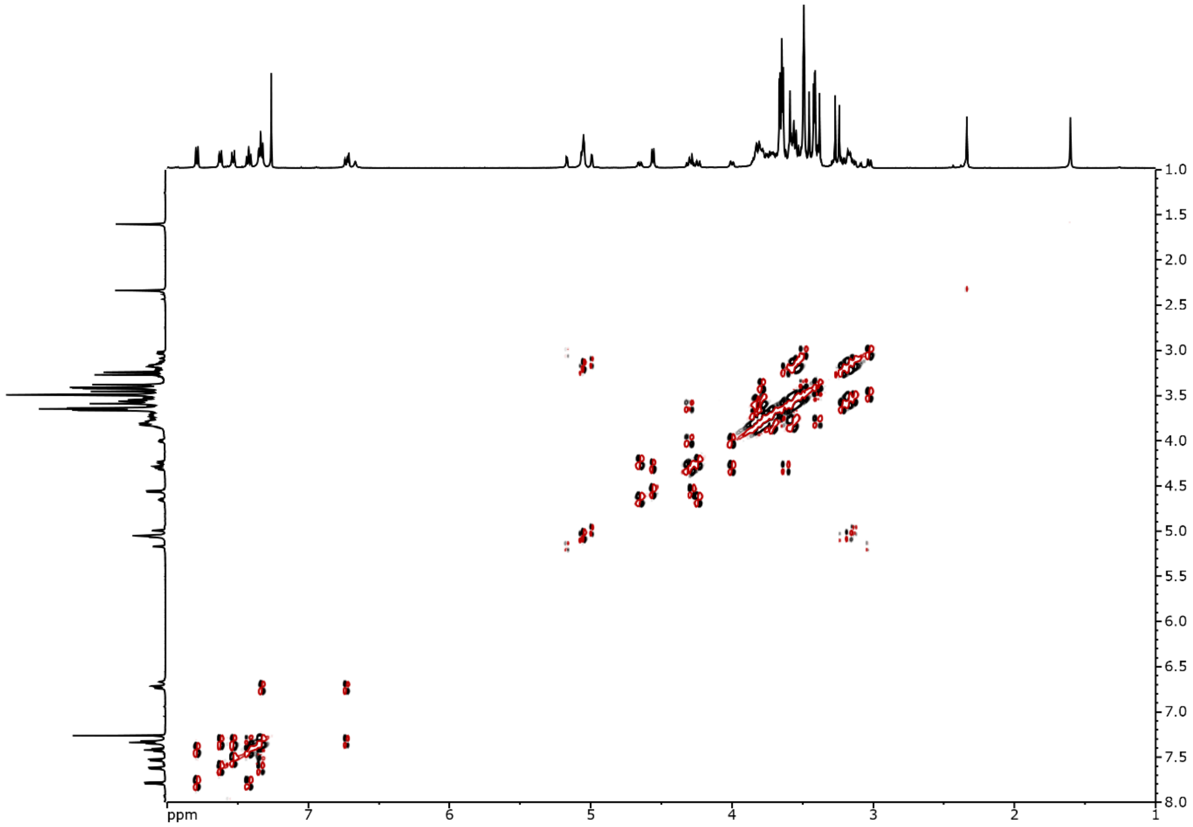

Figure S23. ^1H-^1H DQF-COSY spectrum of **uninc-Fmoc** (500 MHz, $CDCl_3$, 300 K).

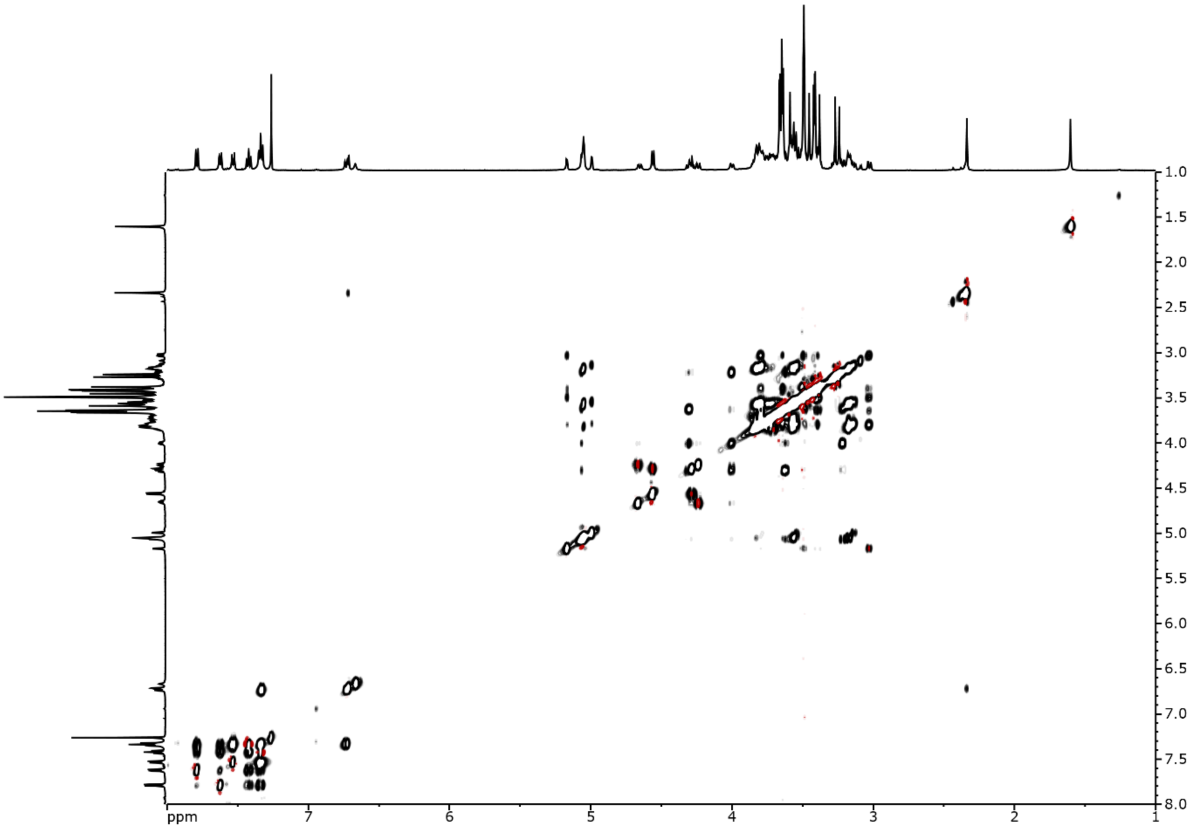

Figure S24. ^1H-^1H TOCSY spectrum of **uninc-Fmoc** (500 MHz, $CDCl_3$, 303 K).

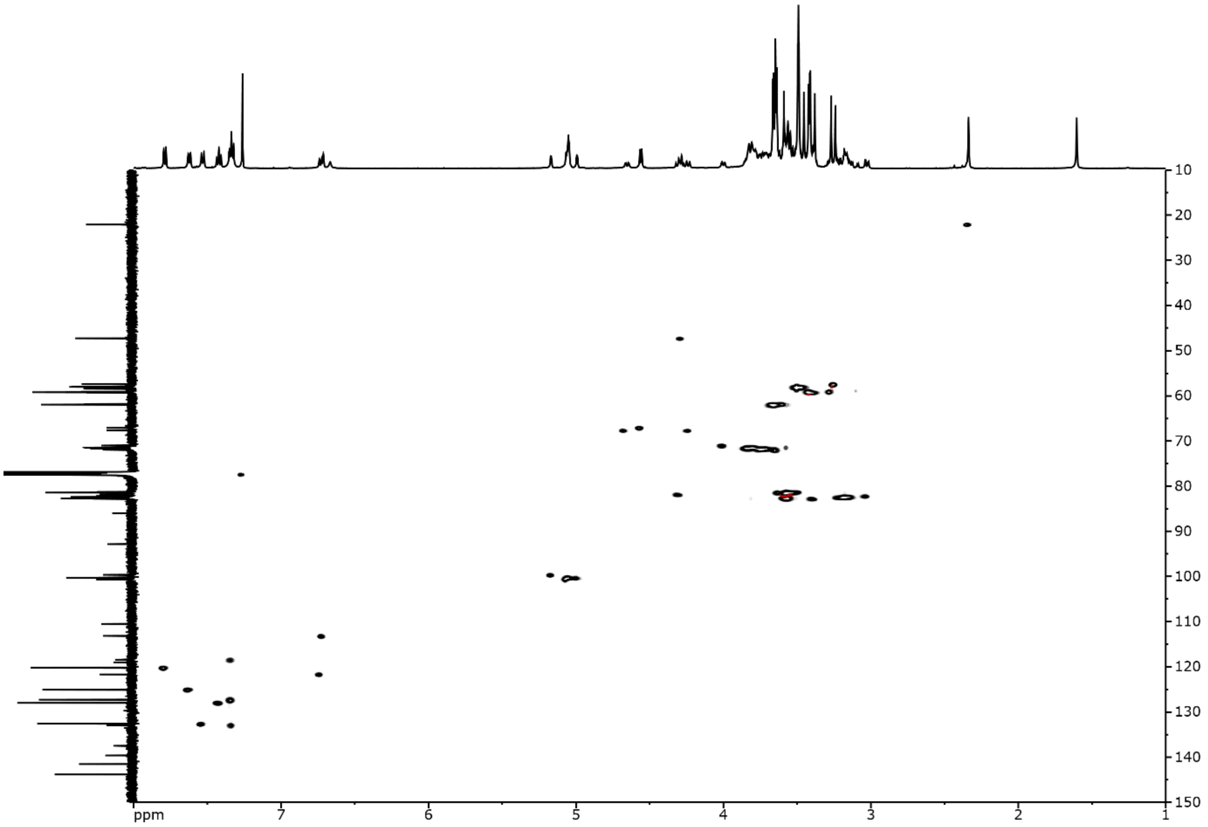

Figure S25. ^{1}H-^{13}C HSQC spectrum of **uninc-Fmoc** (500/126 MHz, $CDCl_3$, 304 K).

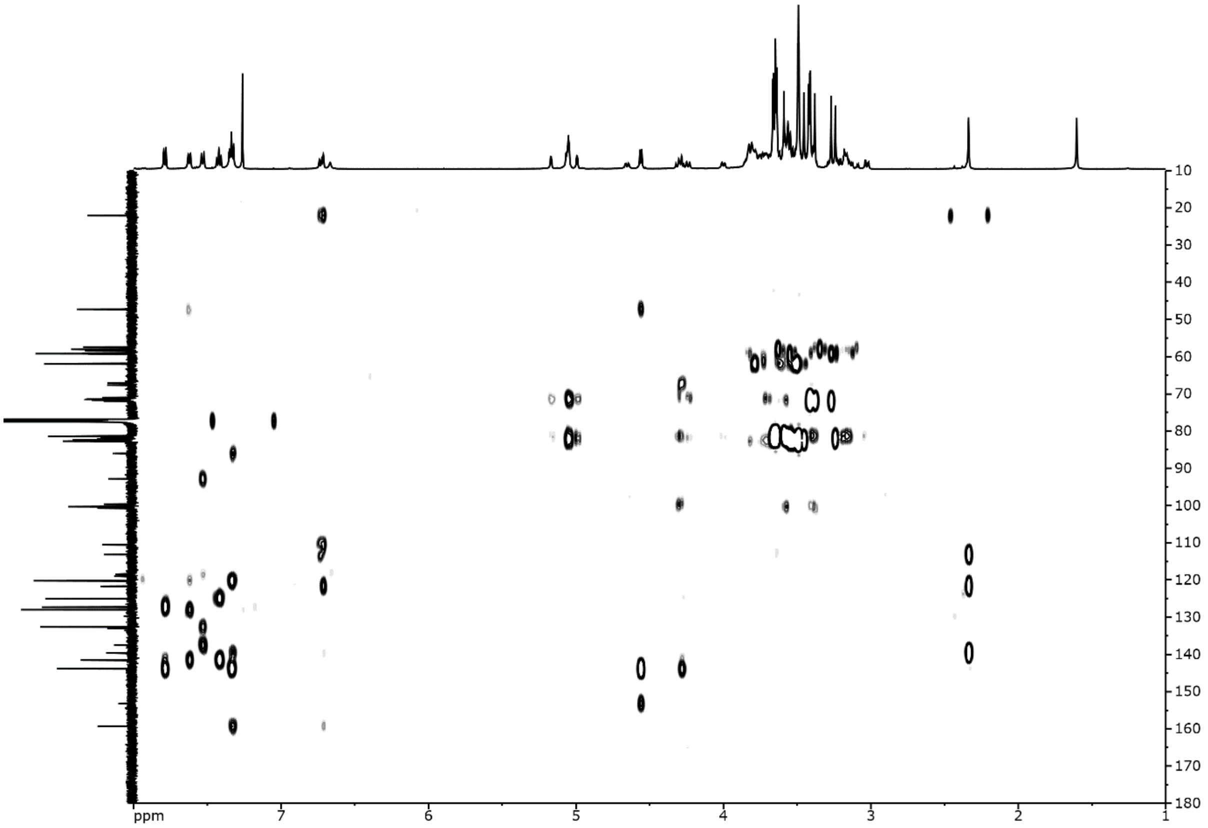

Figure S26. ^{1}H-^{13}C HMBC spectrum of **uninc-Fmoc** (500/126 MHz, $CDCl_3$, 304 K).

4.5 NMR spectra of inc-Fmoc

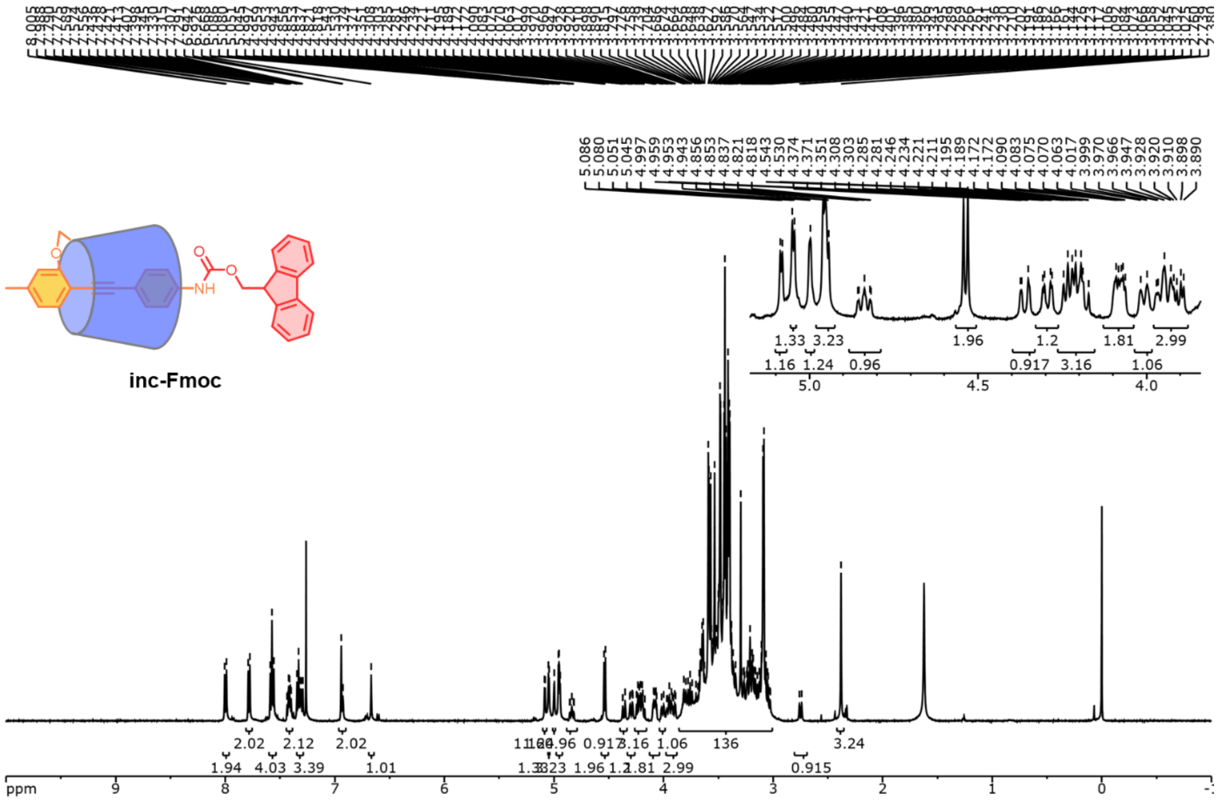

Figure S27. ^{1}H NMR spectrum of **inc-Fmoc** (500 MHz, $CDCl_3$, 302 K).

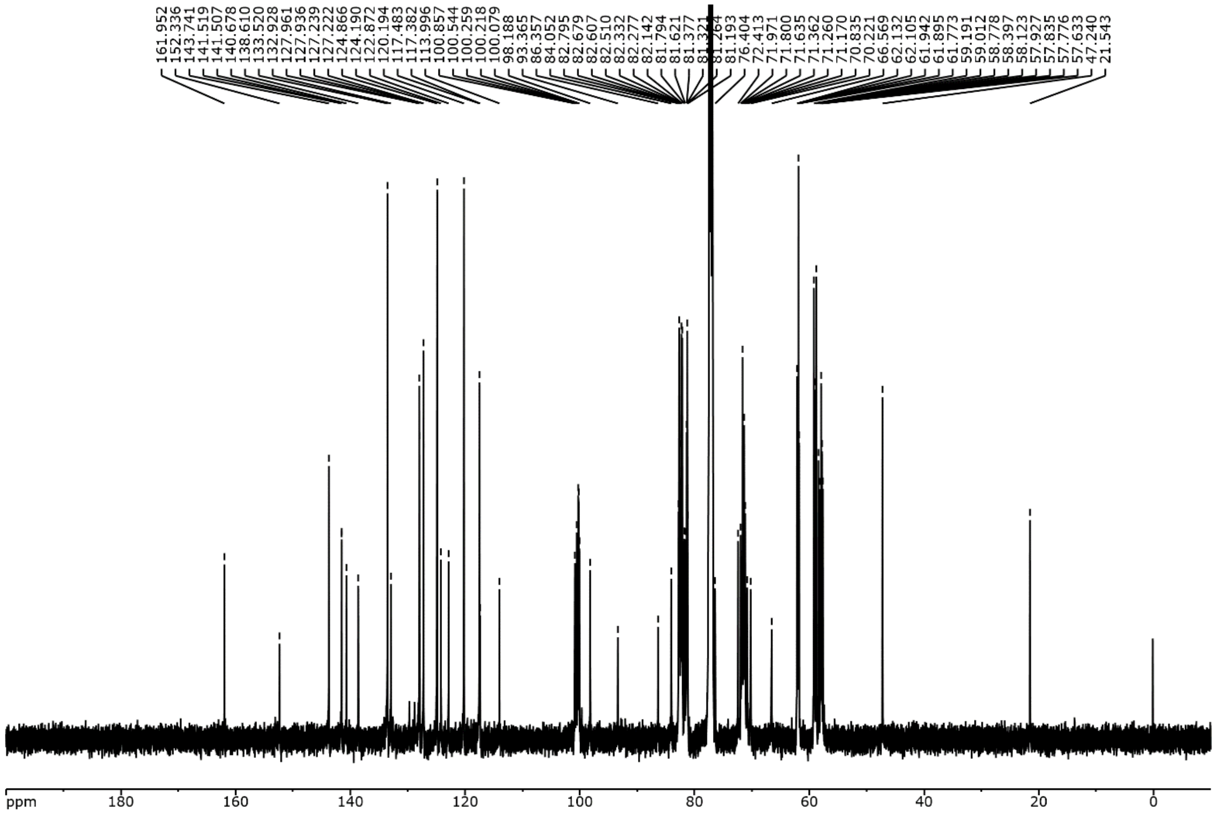

Figure S28. $^{13}C\{^{1}H\}$ NMR spectrum of **inc-Fmoc** (126 MHz, $CDCl_3$, 298 K).

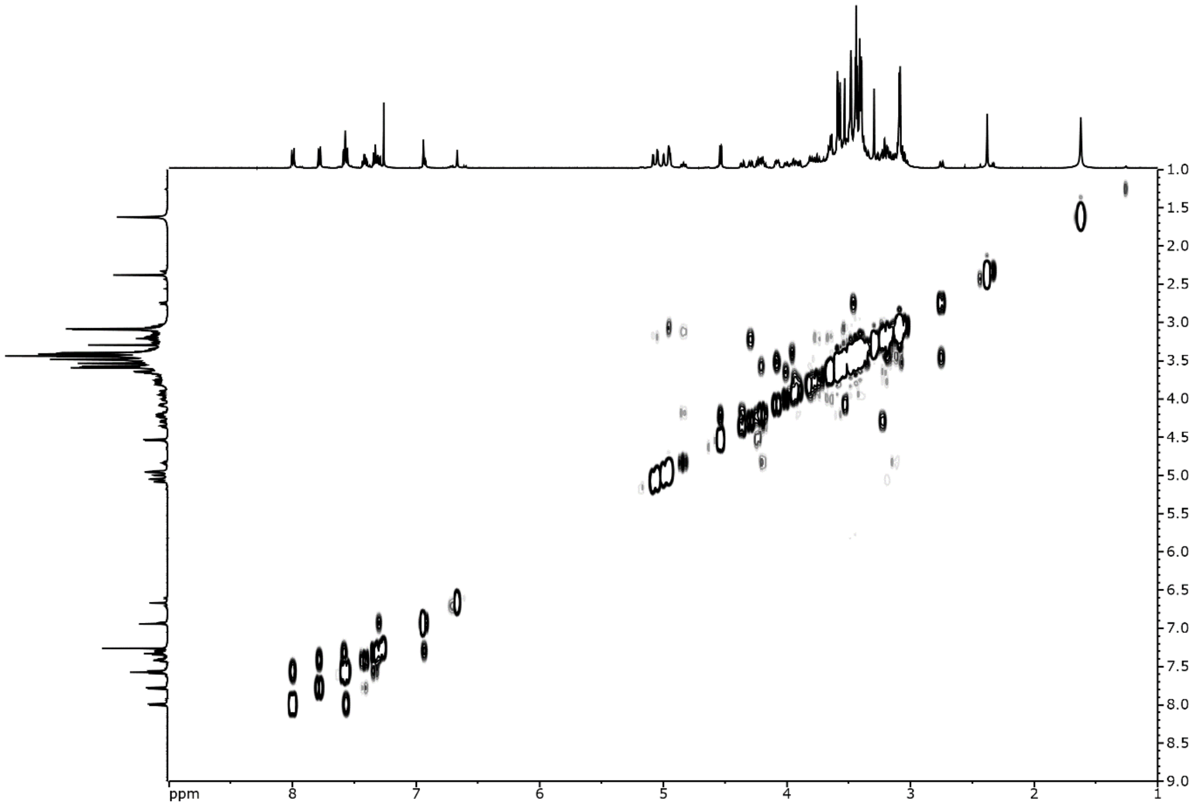

Figure S29. ^{1}H-^{1}H COSY spectrum of **inc-Fmoc** (500 MHz, $CDCl_3$, 302 K).

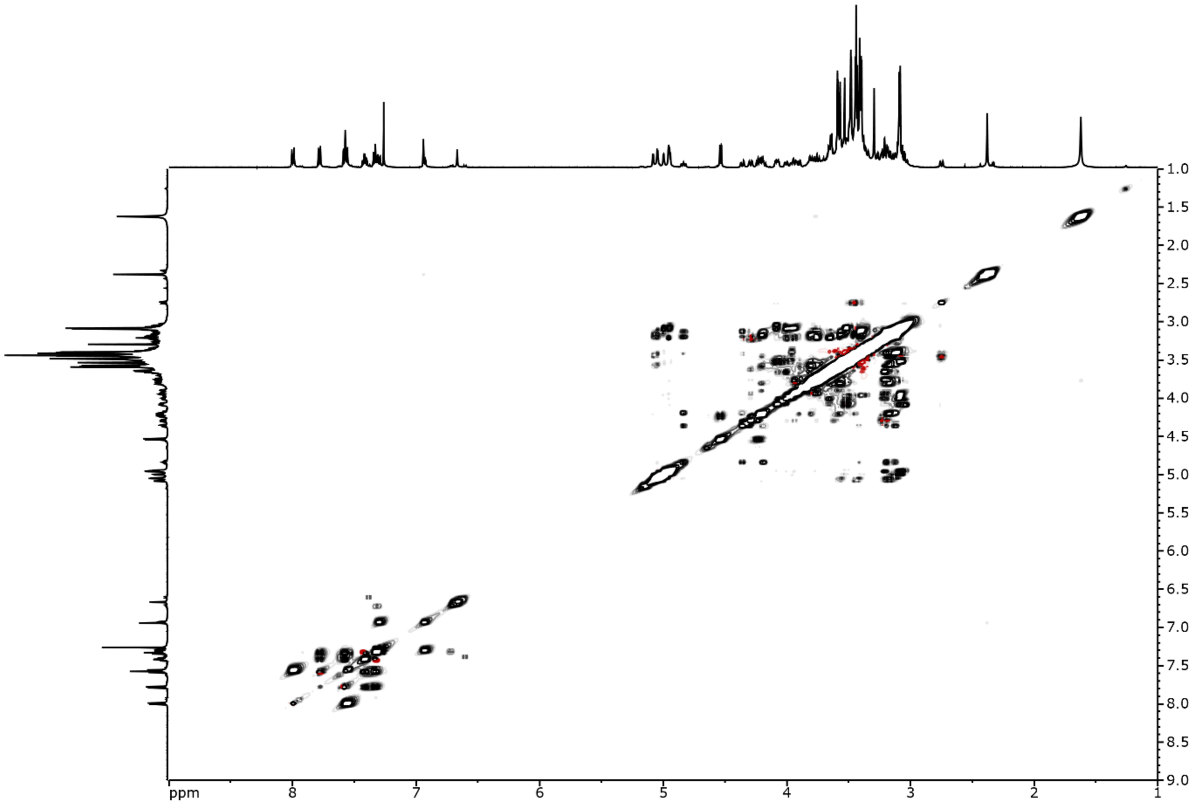

Figure S30. ^{1}H-^{1}H TOCSY spectrum of **inc-Fmoc** (500 MHz, $CDCl_3$, 302 K).

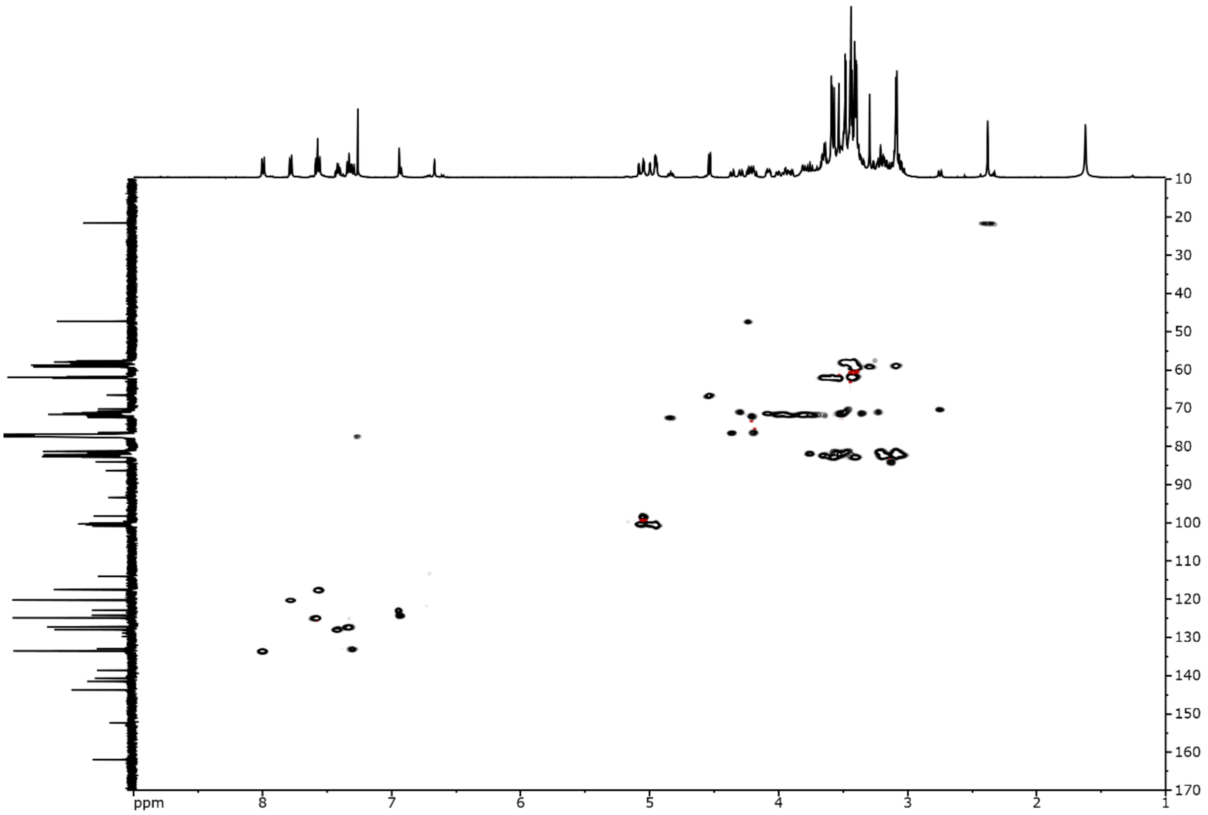

Figure S31. ^{1}H-^{13}C HSQC spectrum of **inc-Fmoc** (500/126 MHz, $CDCl_3$, 302 K).

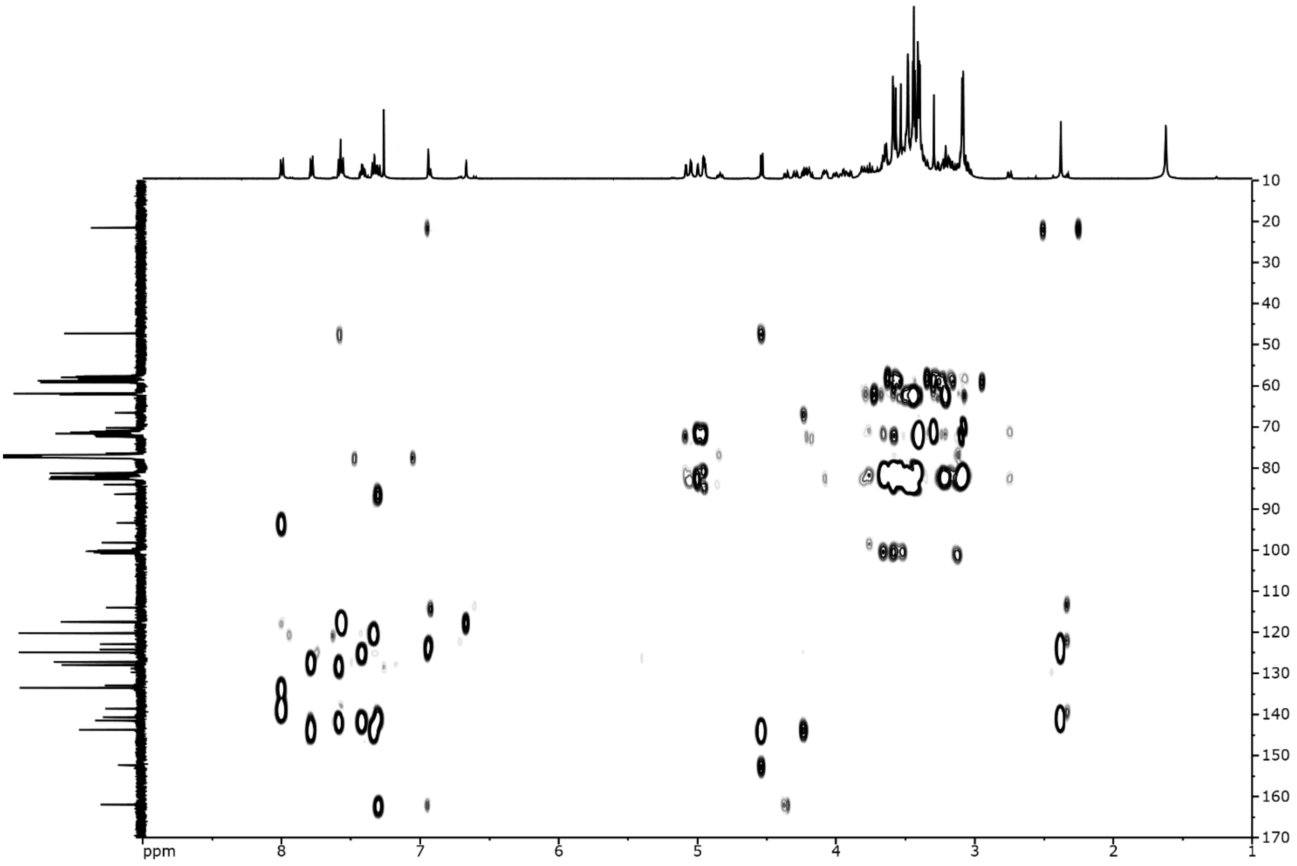

Figure S32. ^{1}H-^{13}C HMBC spectrum of **inc-Fmoc** (500/126 MHz, $CDCl_3$, 302 K).

4.6 NMR spectra of inc

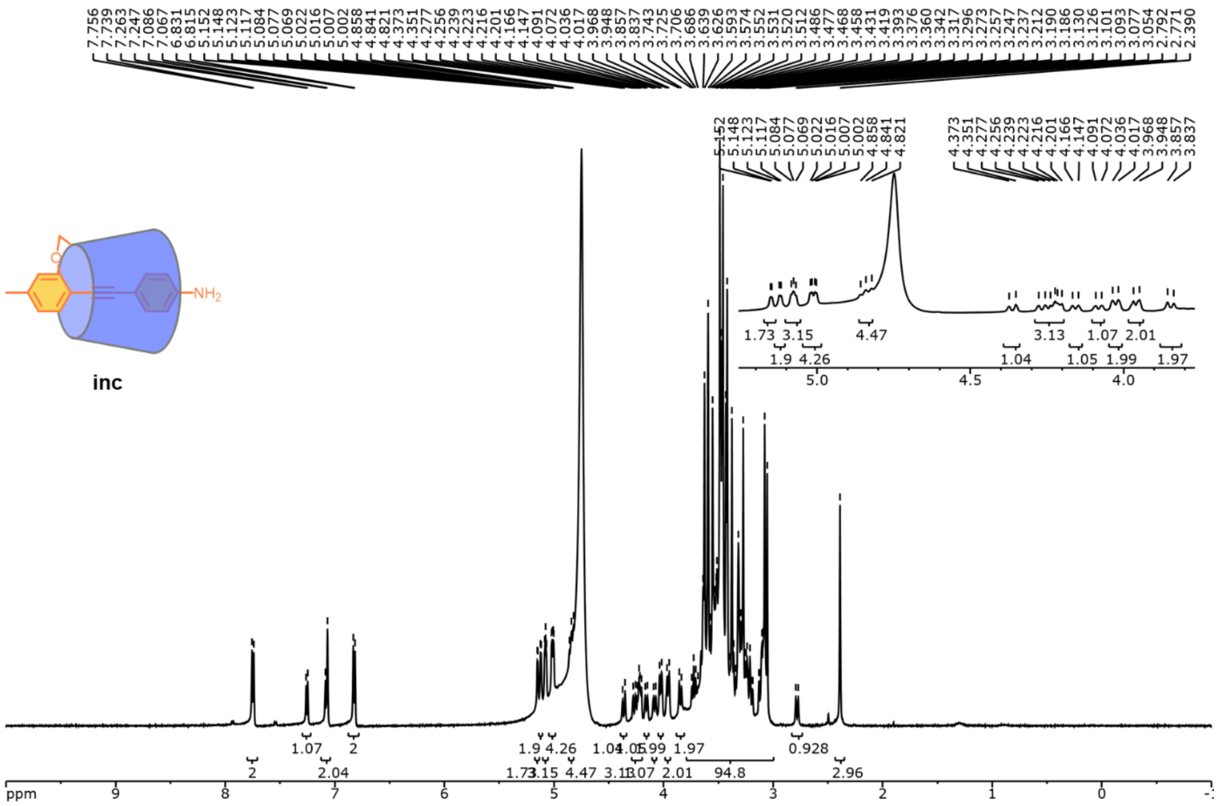

Figure S33. ^{1}H NMR spectrum of **inc** (500 MHz, D_2O/CD_3OD (1:1), 301 K).

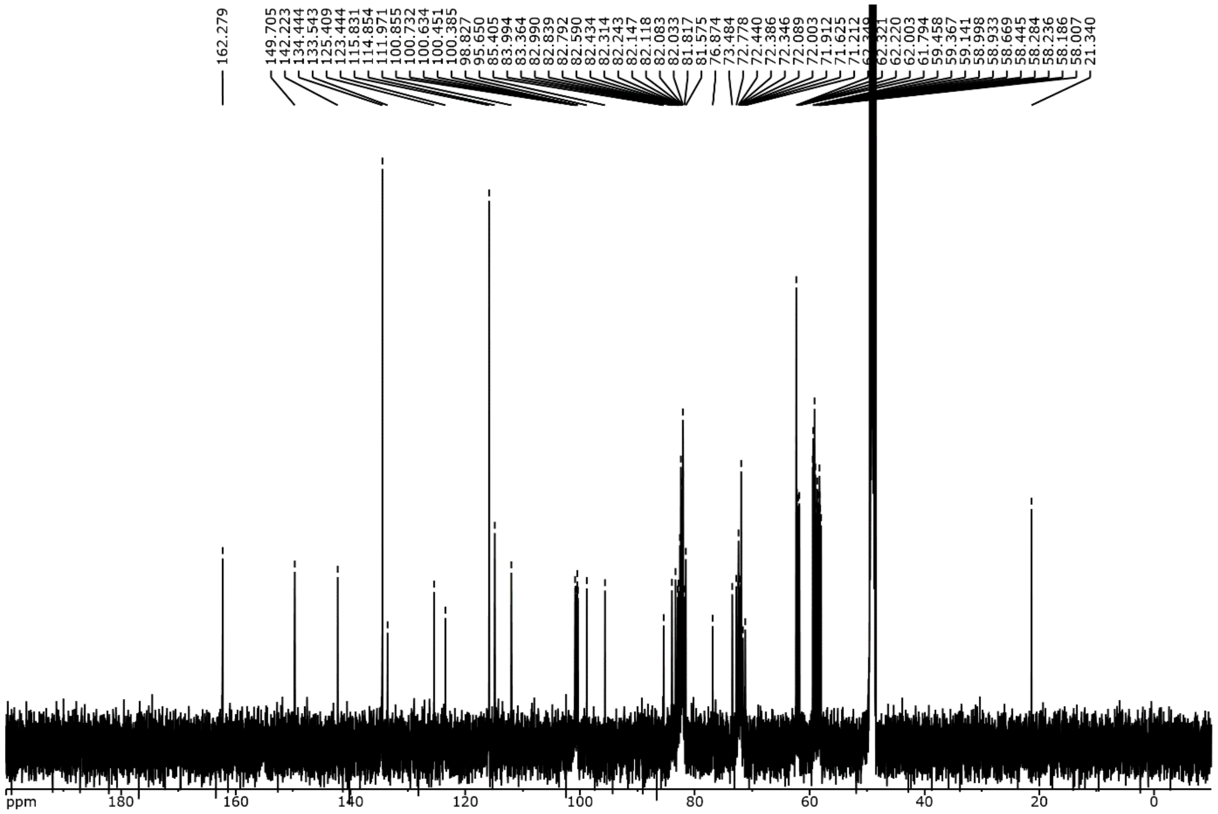

Figure S34. $^{13}C\ \{^{1}H\}$ NMR spectrum of **inc** (126 MHz, D_2O/CD_3OD (1:1), 303 K).

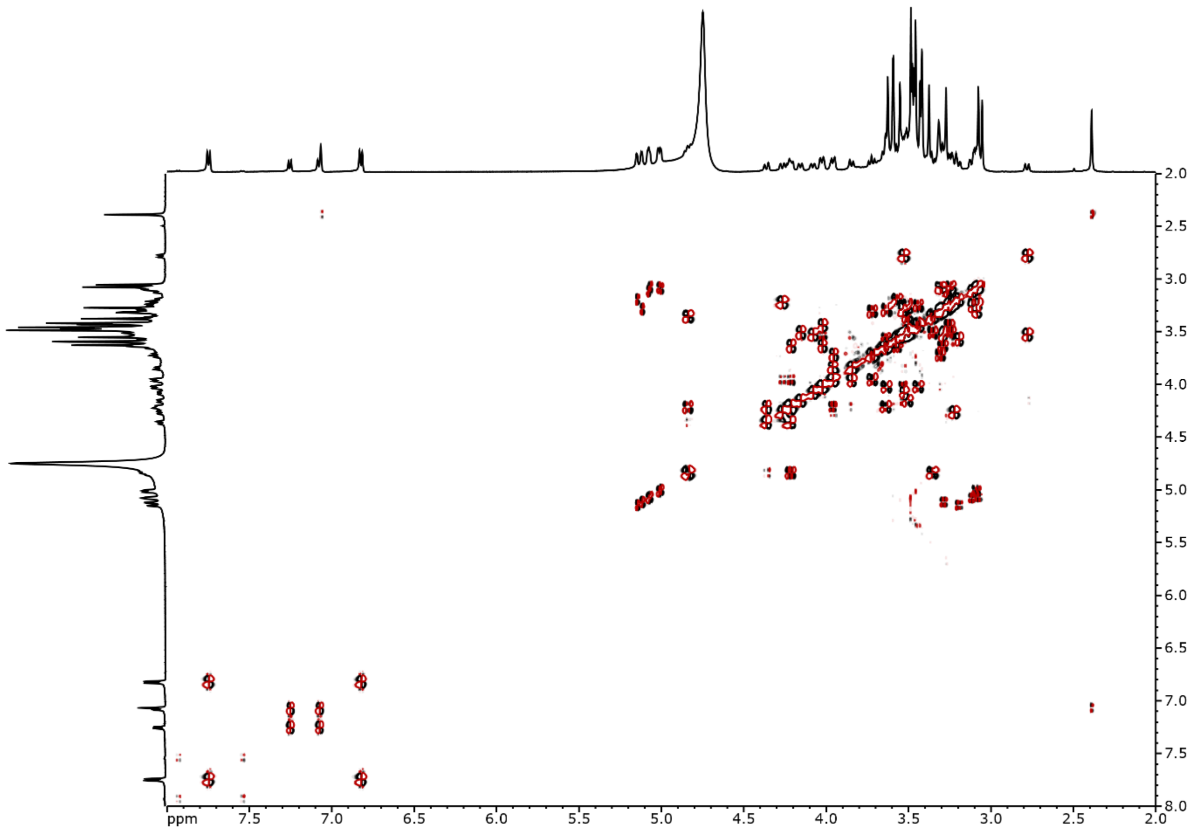

Figure S35. ^{1}H-^{1}H DQF-COSY spectrum of **inc** (500 MHz, D_2O/CD_3OD (1:1), 303 K).

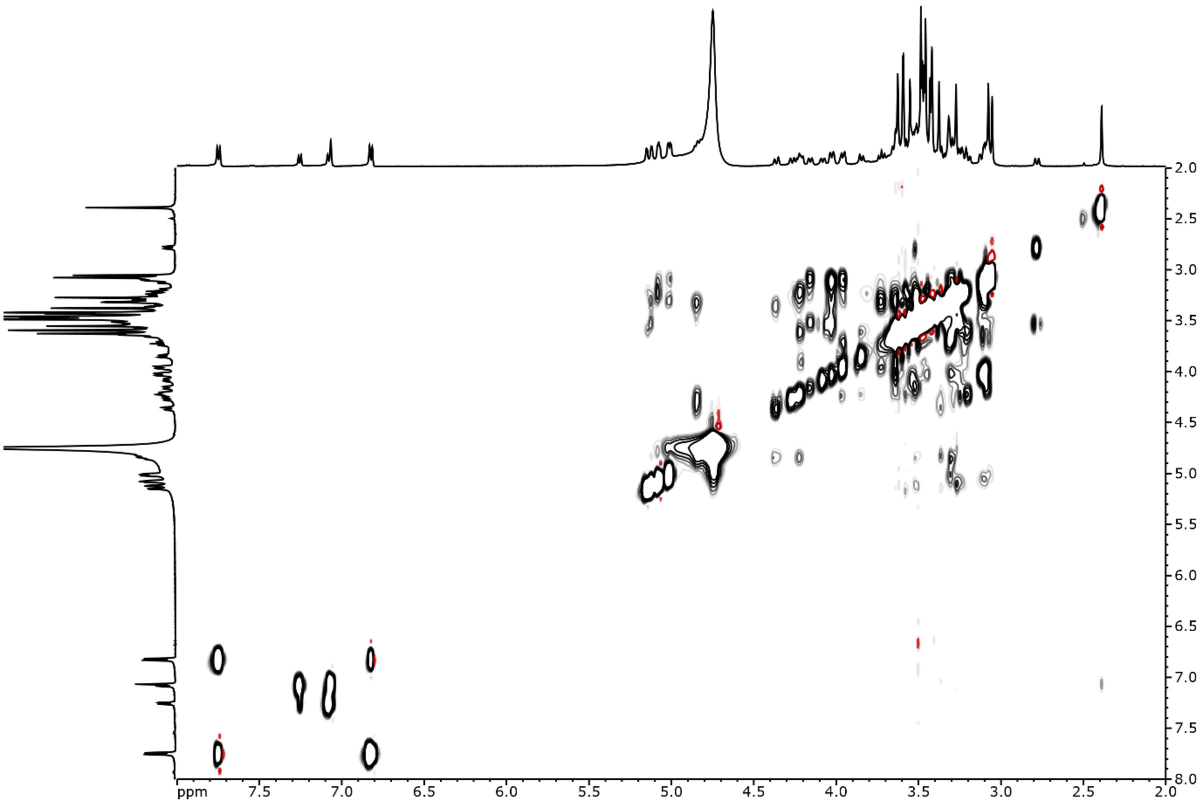

Figure S36. ^{1}H-^{1}H TOCSY spectrum of **inc** (500 MHz, D_2O/CD_3OD (1:1), 302 K).

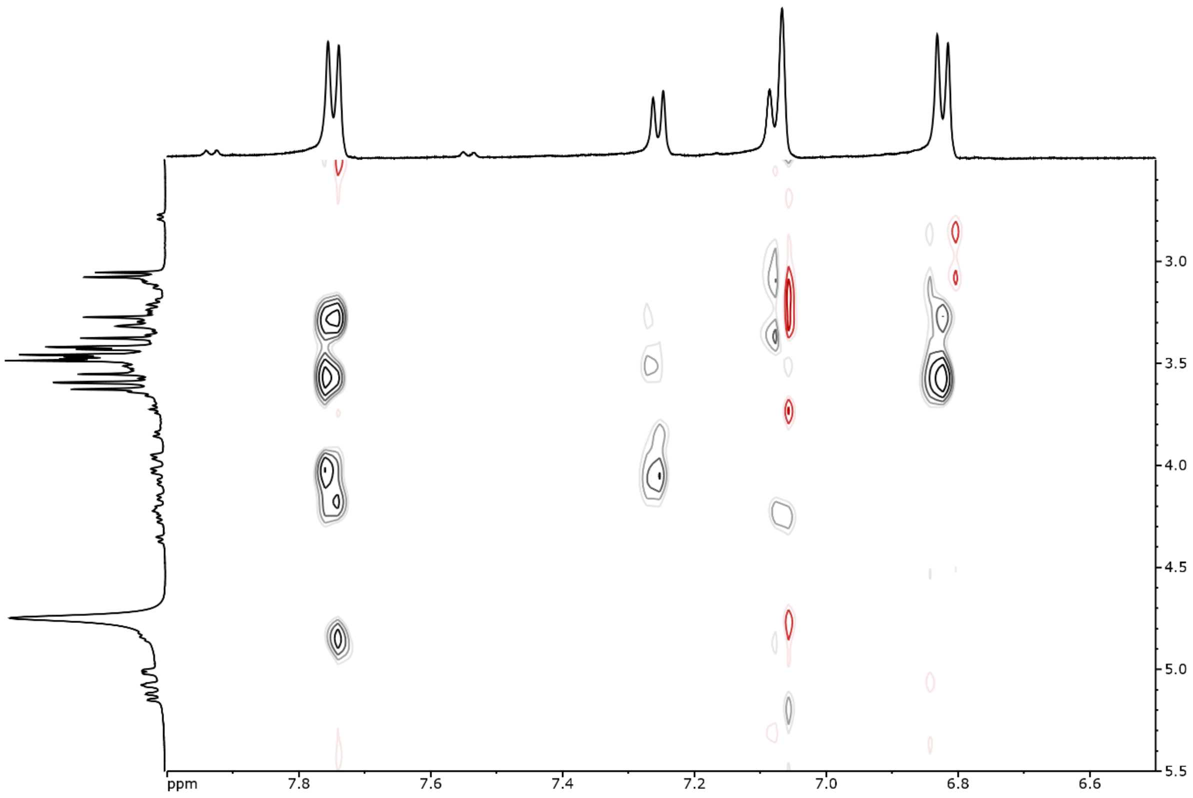

Figure S37. ^1H-^1H ROESY spectrum of **inc** (500 MHz, D_2O/CD_3OD (1:1), 302 K).

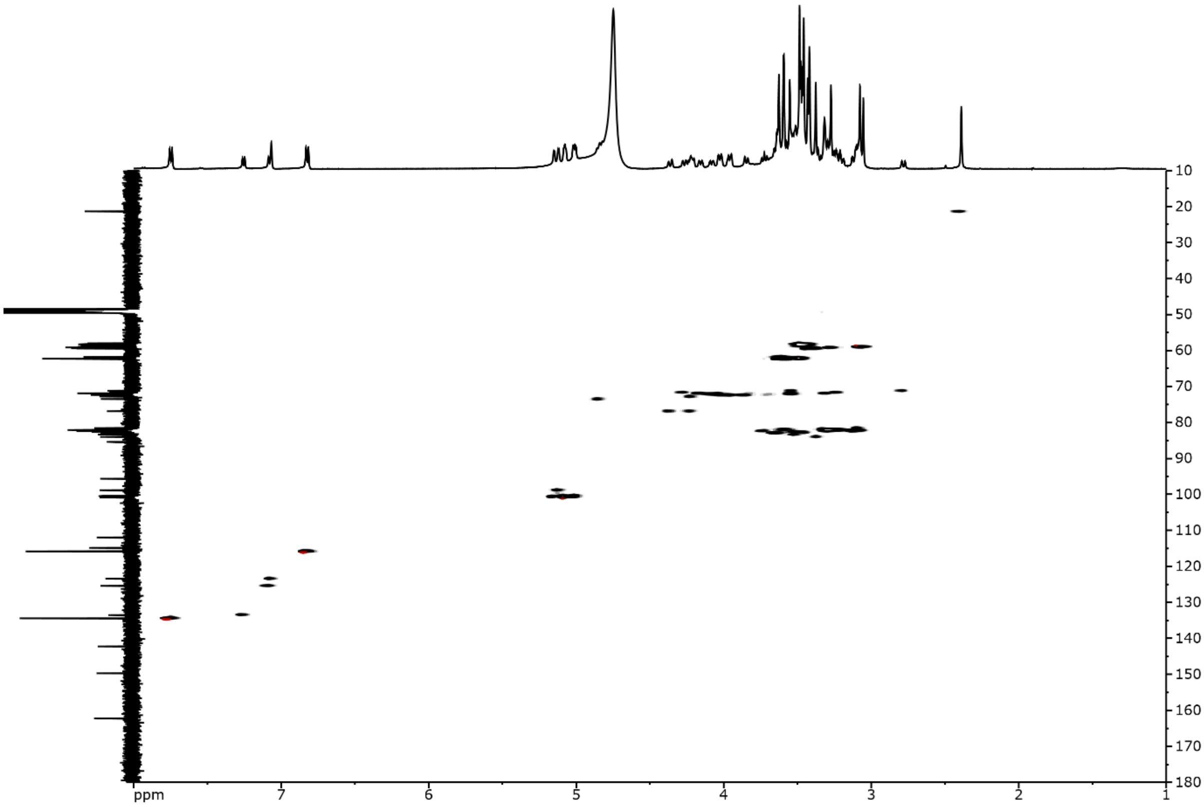

Figure S38. ^1H-^{13}C HSQC spectrum of **inc** (500/126 MHz, D_2O/CD_3OD (1:1), 302 K).

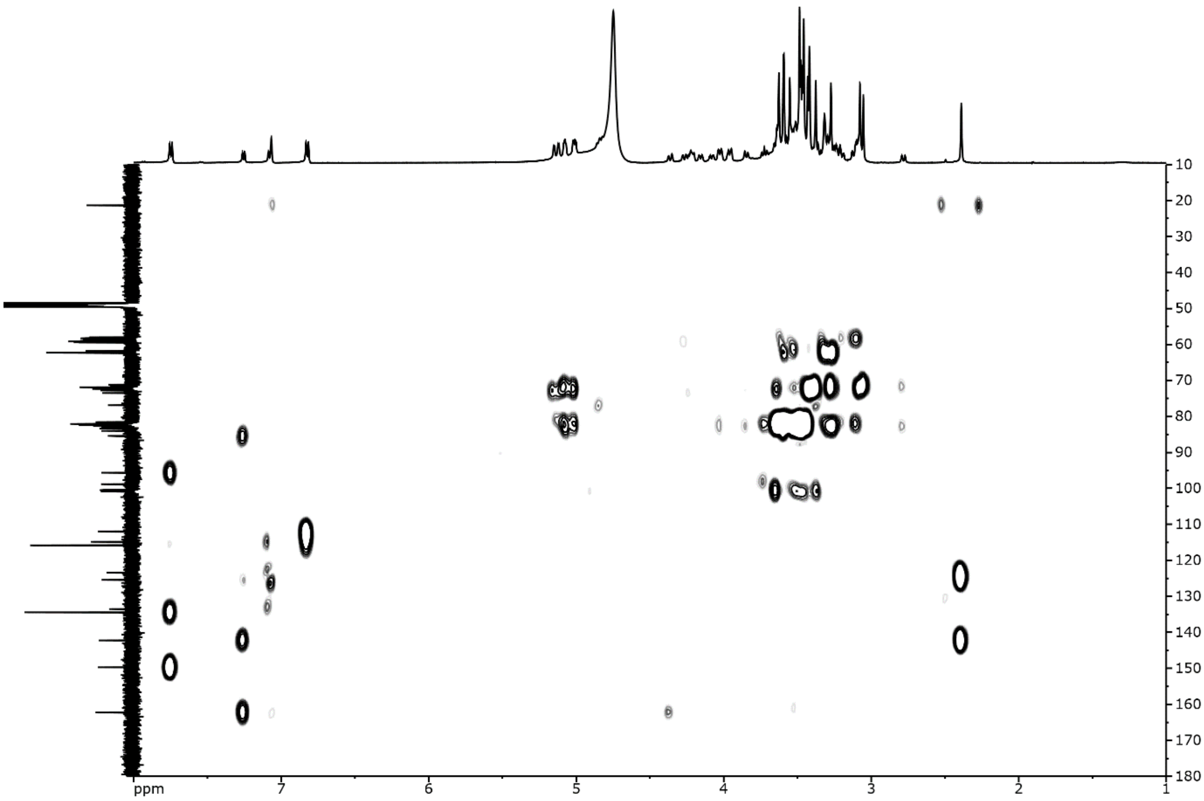

Figure S39. ^1H-^{13}C HMBC spectrum of **inc** (500/126 MHz, D_2O/CD_3OD (1:1), 302 K).

4.7 NMR spectra of S2

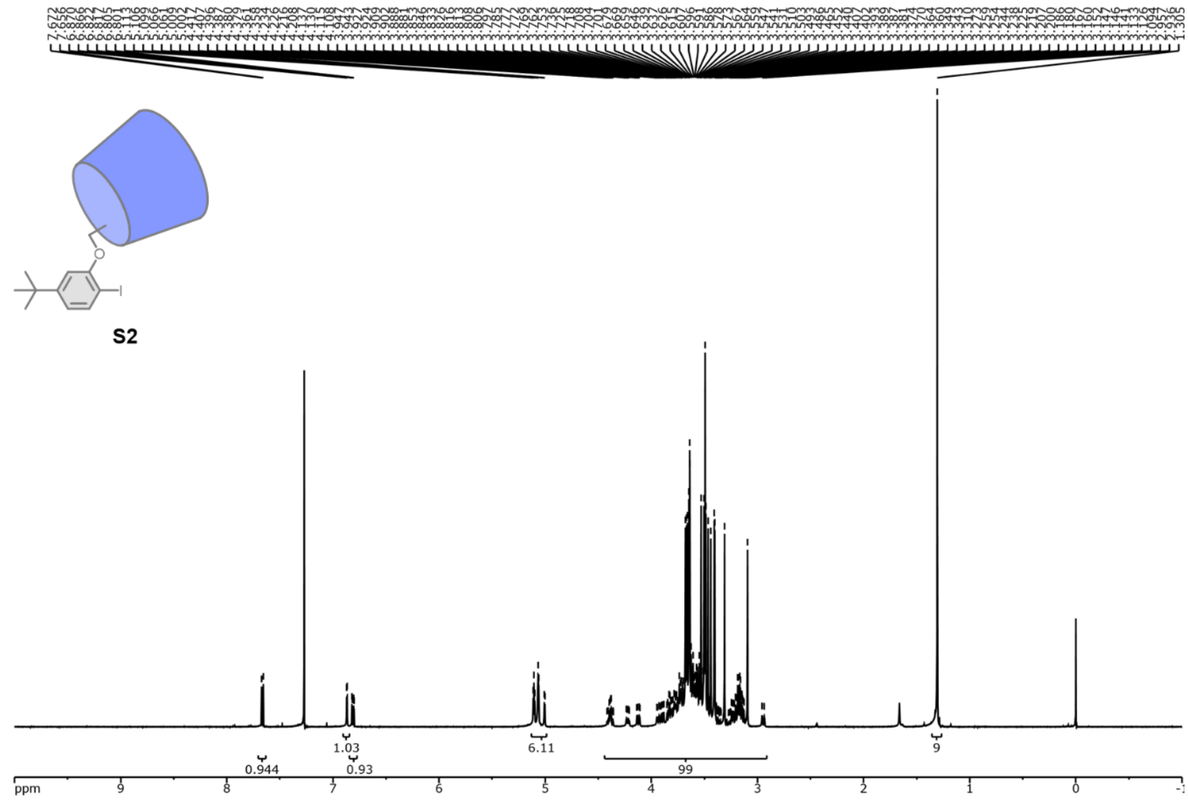

Figure S40. ^{1}H NMR spectrum of **S2** (500 MHz, $CDCl_3$, 298 K).

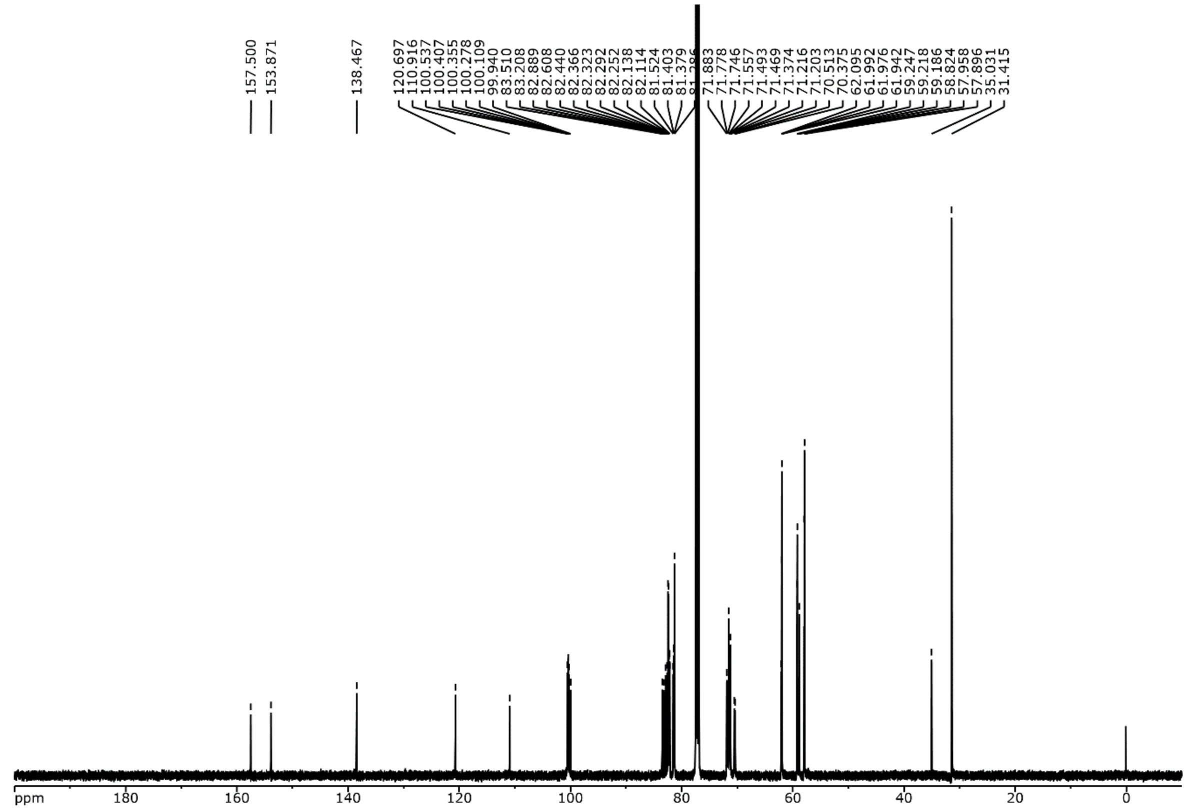

Figure S41. ^{13}C {^{1}H} NMR spectrum of **S2** (126 MHz, $CDCl_3$, 299 K).

4.8 NMR spectra of S3

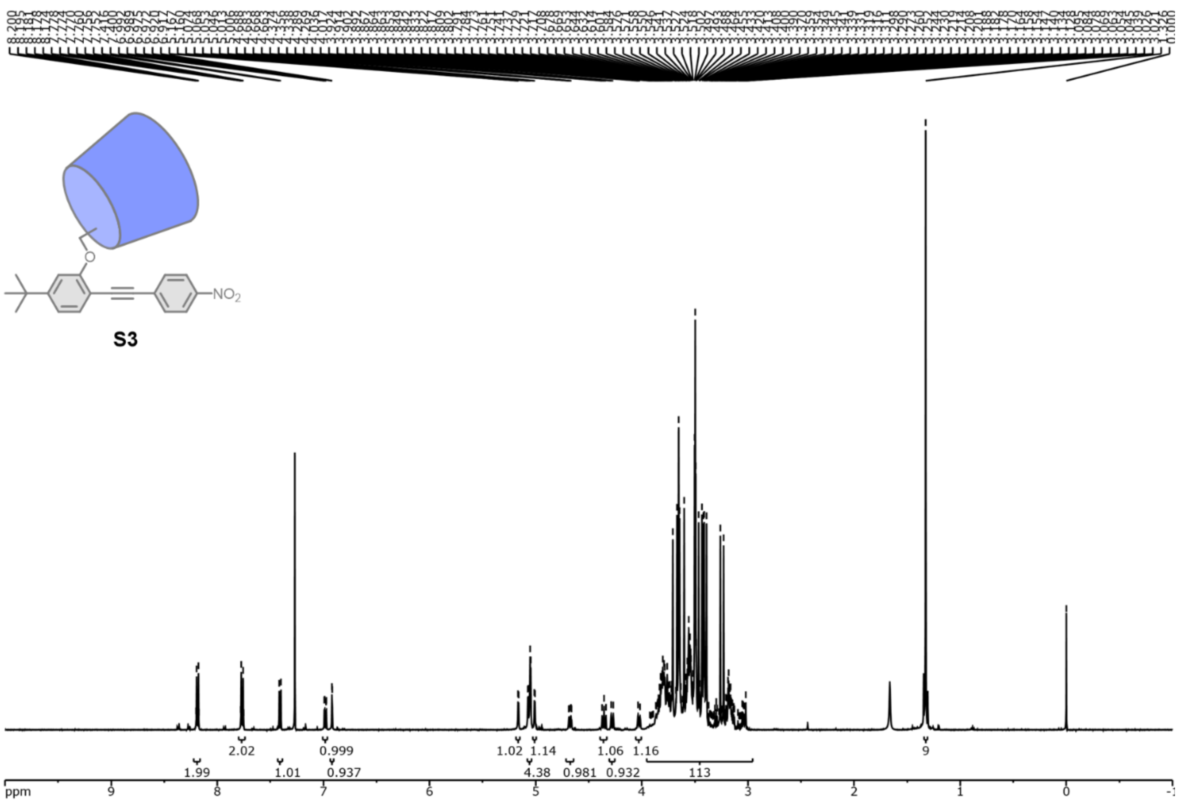

Figure S42. ^{1}H NMR spectrum of **S3** (500 MHz, $CDCl_3$, 298 K).

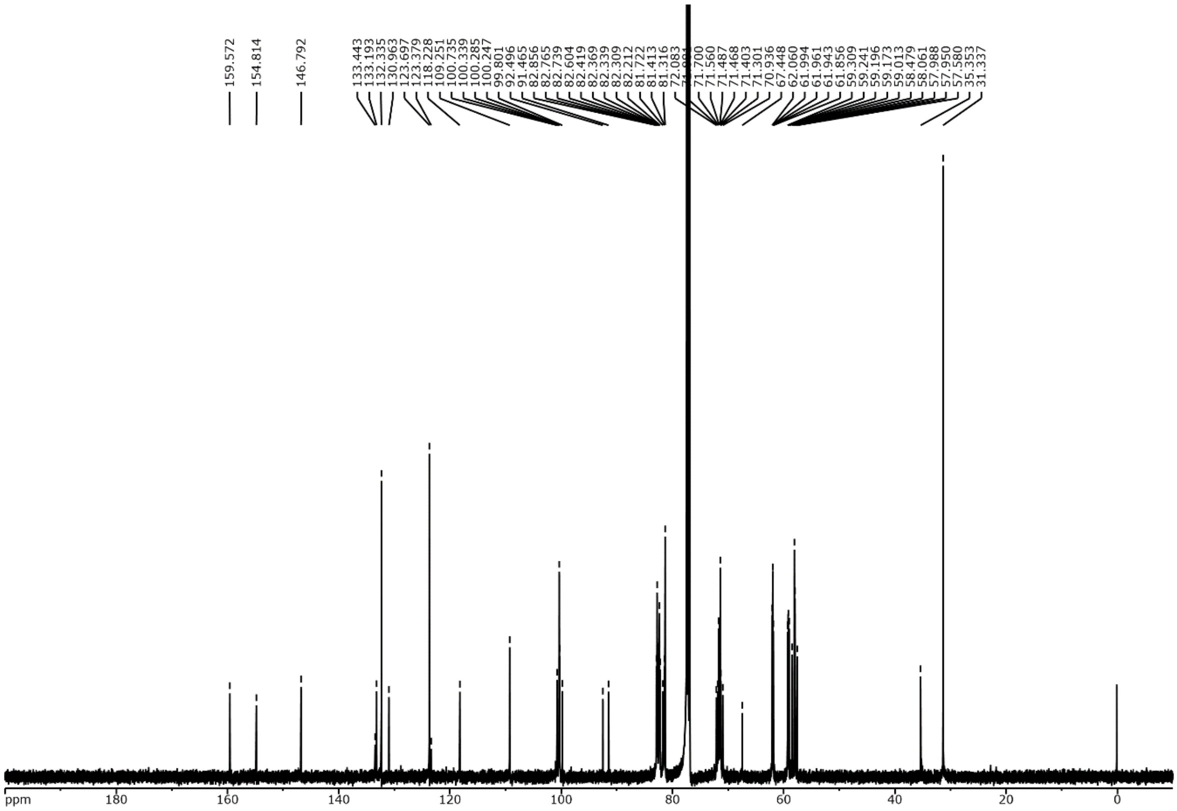

Figure S43. ^{13}C {^{1}H} NMR spectrum of **S3** (126 MHz, $CDCl_3$, 298 K).

4.9 NMR spectra of uninc-^{t}Bu

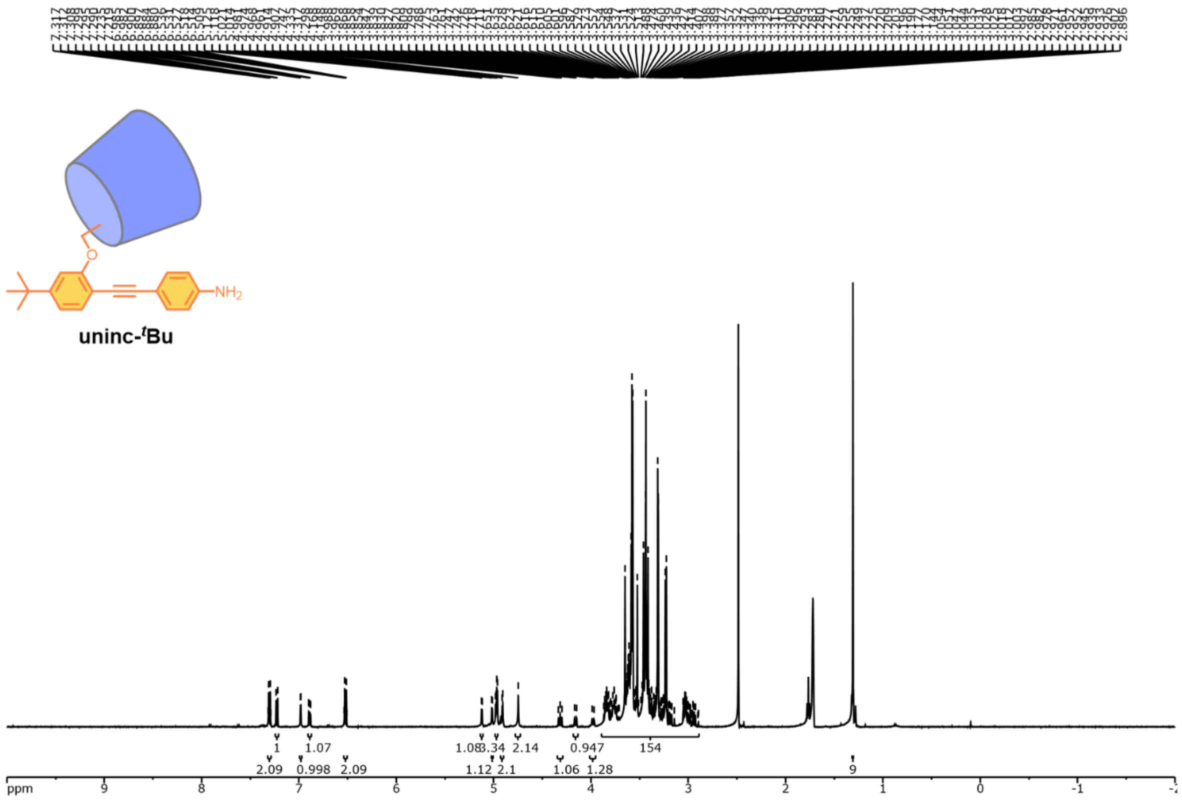

Figure S44. ^{1}H NMR spectrum of **uninc-^{t}Bu** (500 MHz, THF-d_8, 298 K).

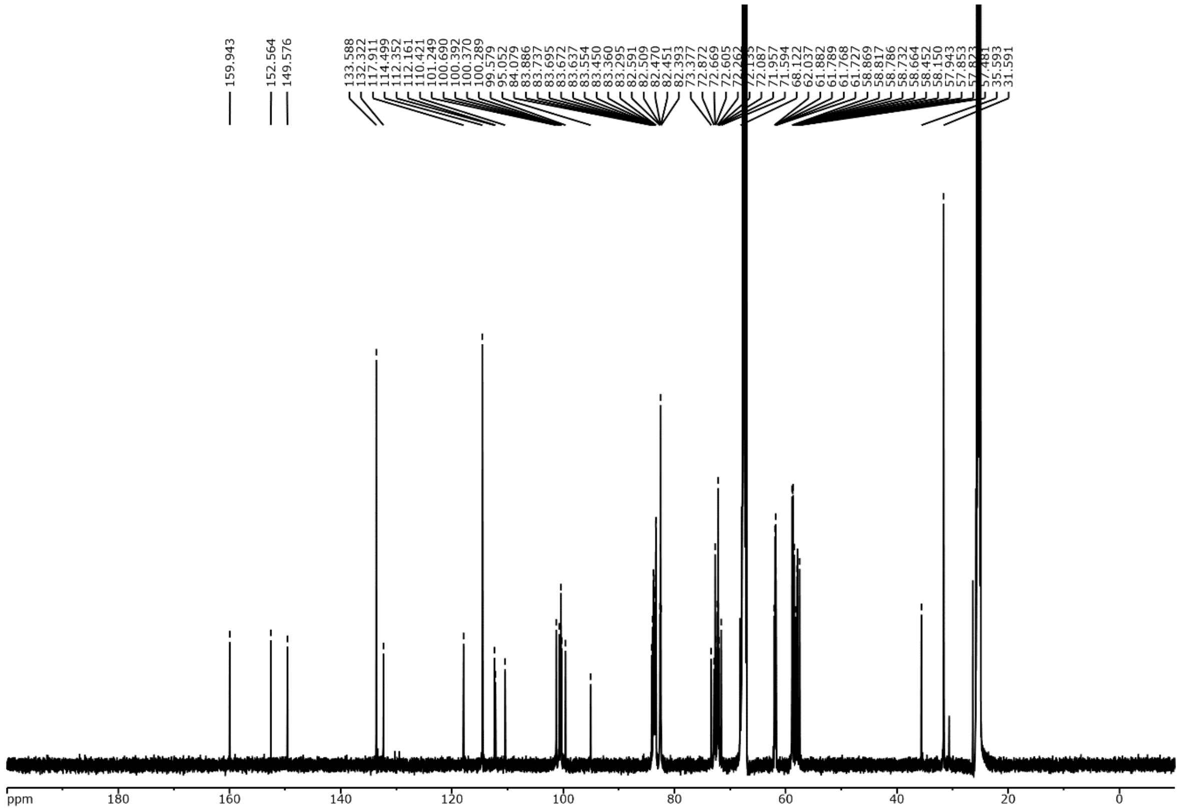

Figure S45. ^{13}C {^{1}H} NMR spectrum of **uninc-^{t}Bu** (126 MHz, THF-d_8, 299 K).

4.10 NMR spectra of uninc-^{t}Bu-Fmoc

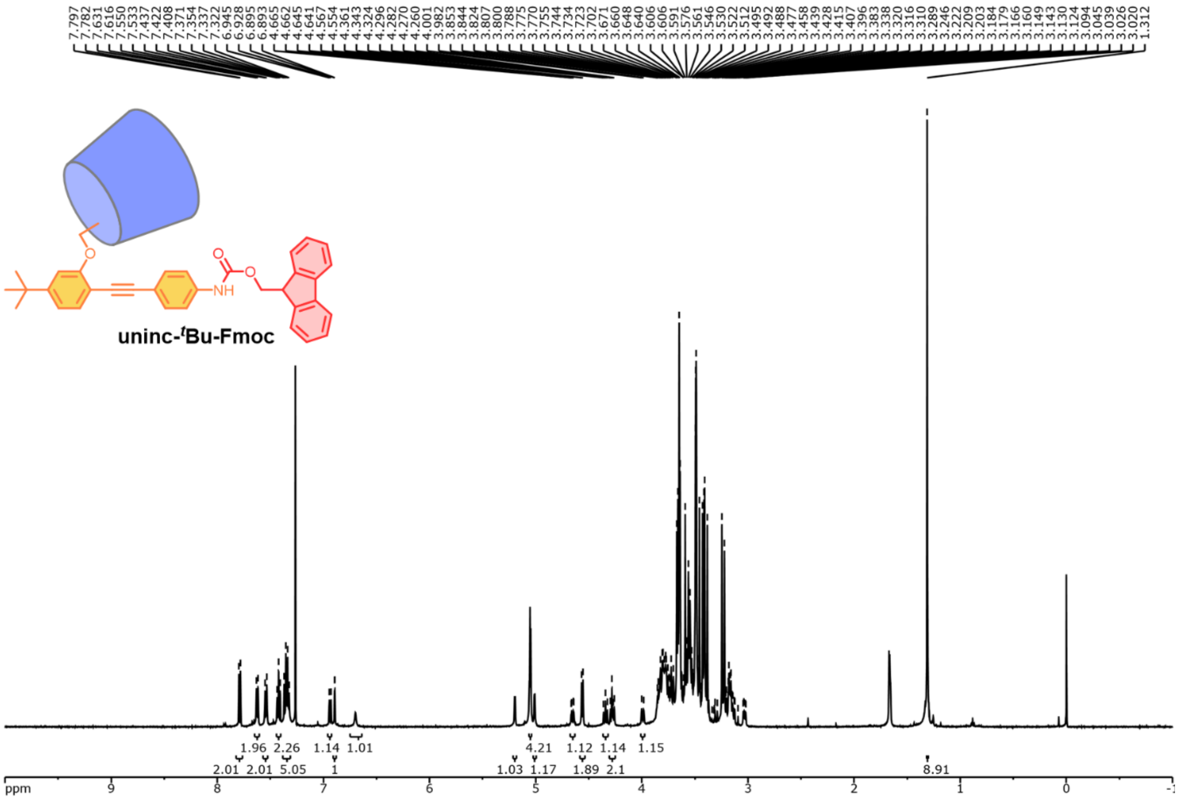

Figure S46. ^{1}H NMR spectrum of **uninc-^{t}Bu-Fmoc** (500 MHz, $CDCl_3$, 298 K).

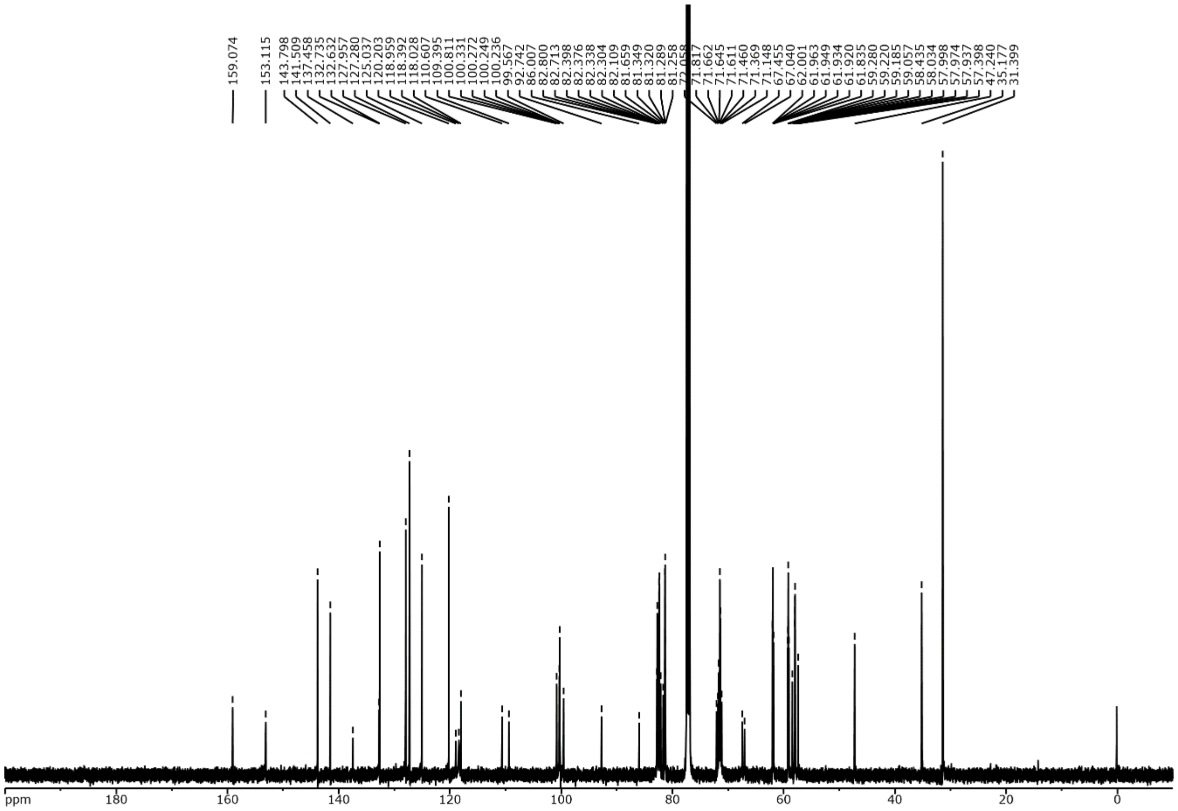

Figure S47. ^{13}C {^{1}H} NMR spectrum of **uninc-^{t}Bu-Fmoc** (126 MHz, $CDCl_3$, 300 K).

4.11 NMR spectra of inc-^{t}Bu-Fmoc

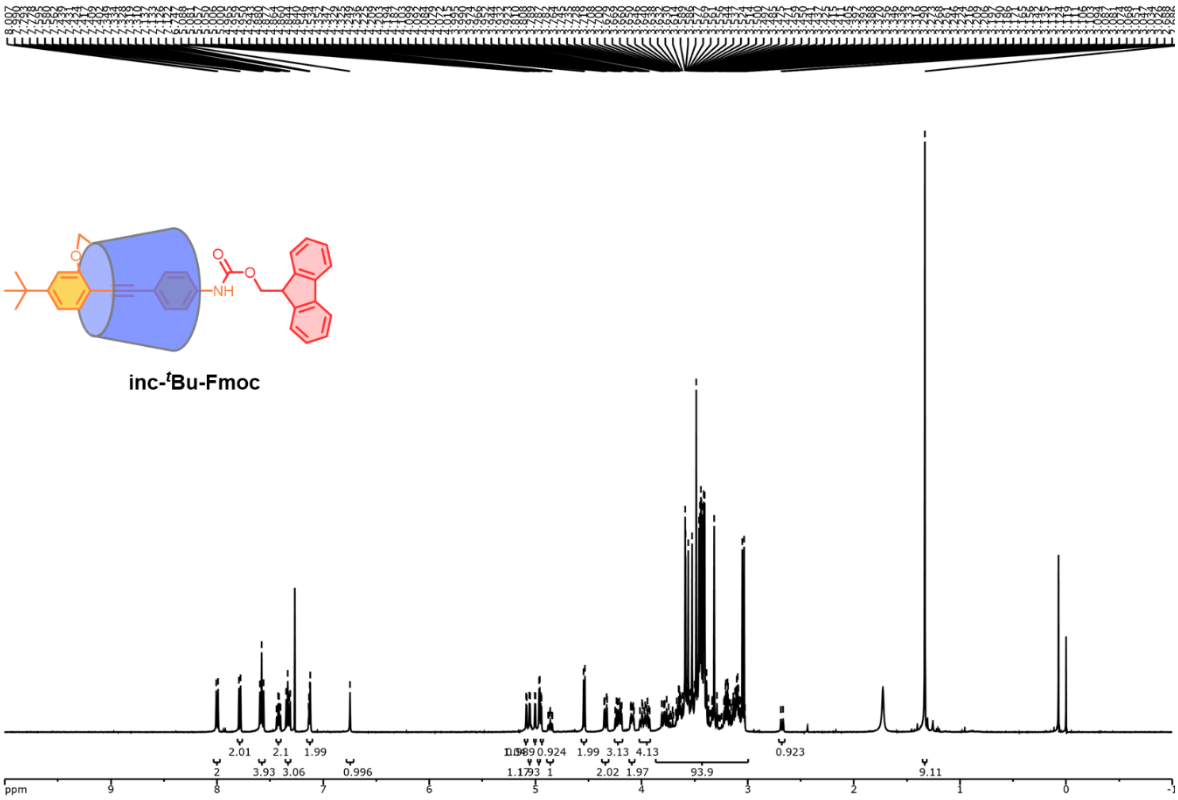

Figure S48. ^{1}H NMR spectrum of **inc-^{t}Bu-Fmoc** (500 MHz, $CDCl_3$, 298 K).

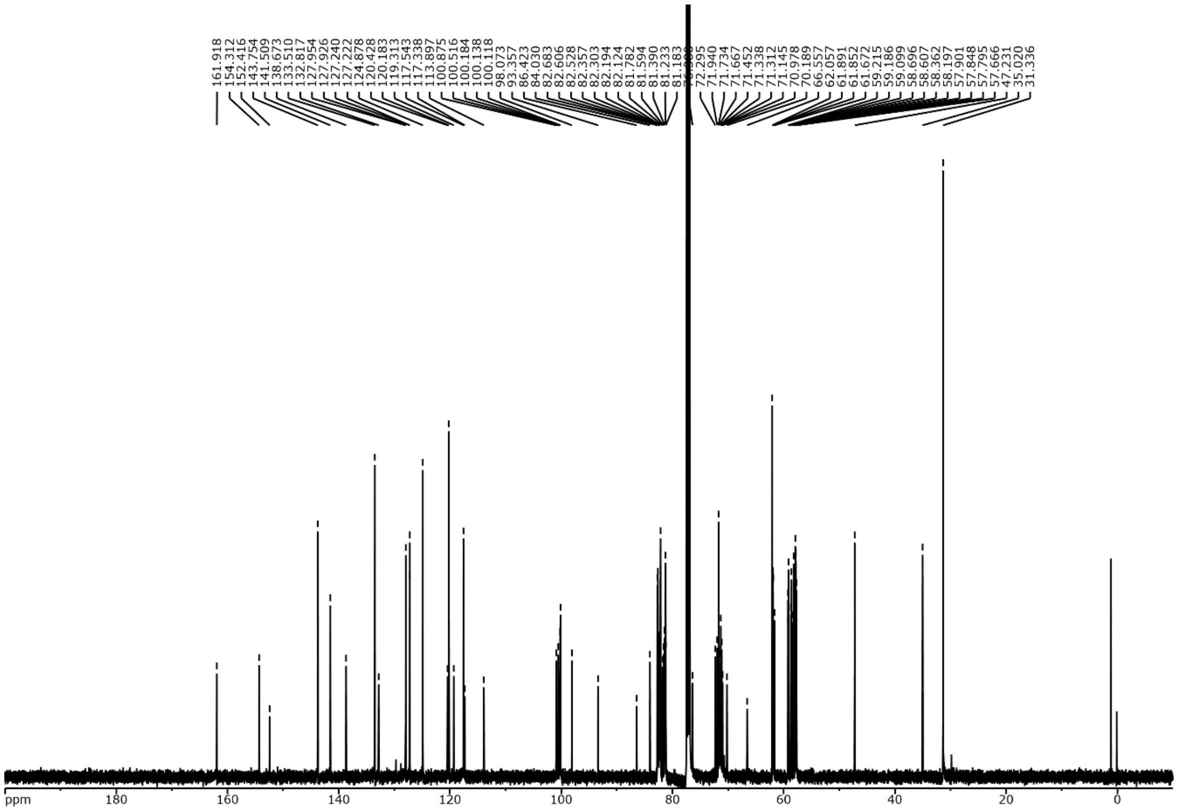

Figure S49. ^{13}C {^{1}H} NMR spectrum of **inc-^{t}Bu-Fmoc** (126 MHz, $CDCl_3$, 300 K).

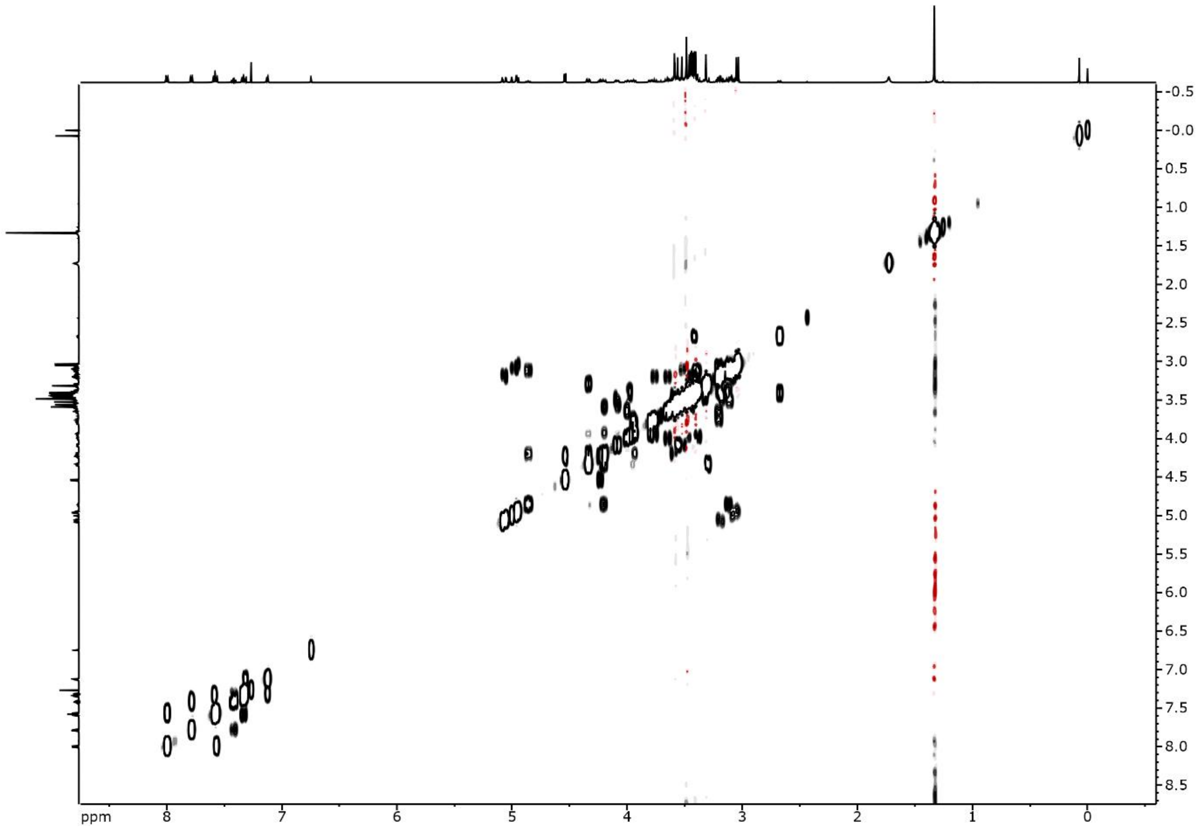

Figure S50. ^{1}H-^{1}H COSY spectrum of **inc-^{t}Bu-Fmoc** (500 MHz, $CDCl_3$, 298 K).

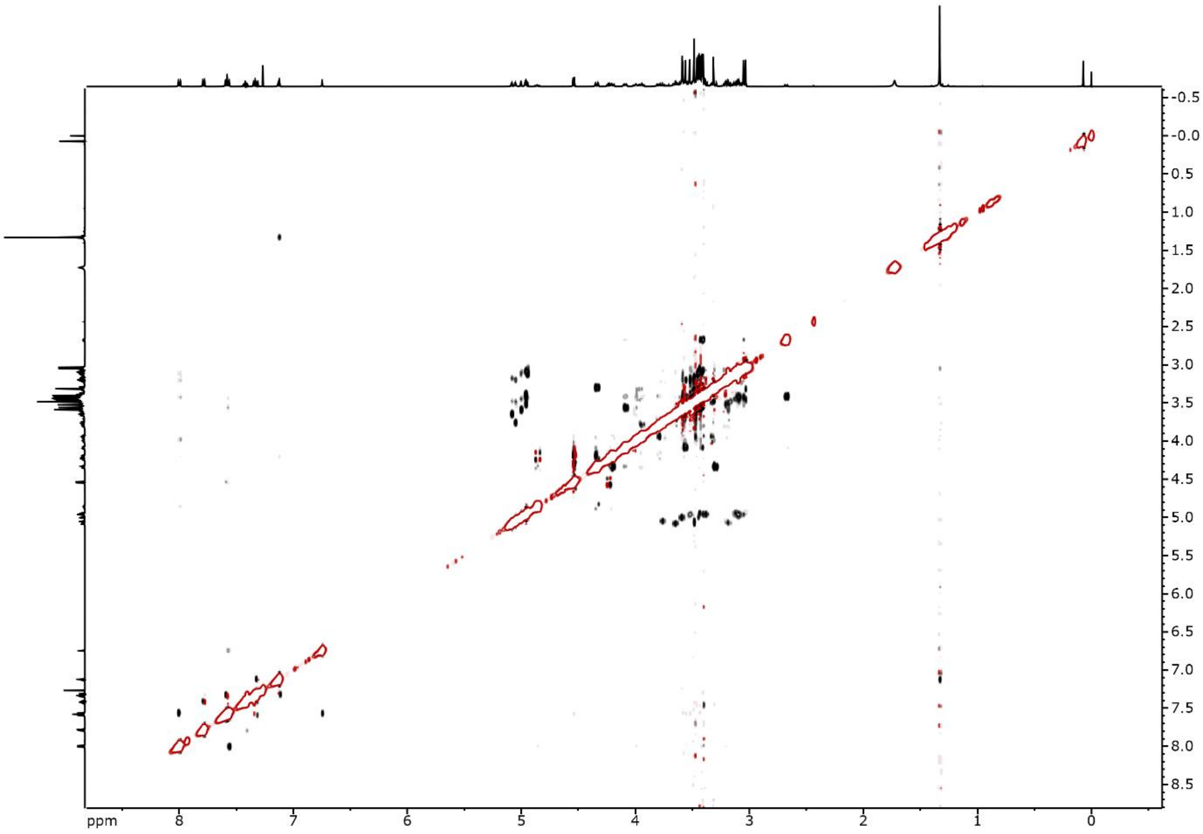

Figure S51. ^{1}H-^{1}H ROESY spectrum of **inc-^{t}Bu-Fmoc** (500 MHz, $CDCl_3$, 298 K).

4.12 HR-MS spectra

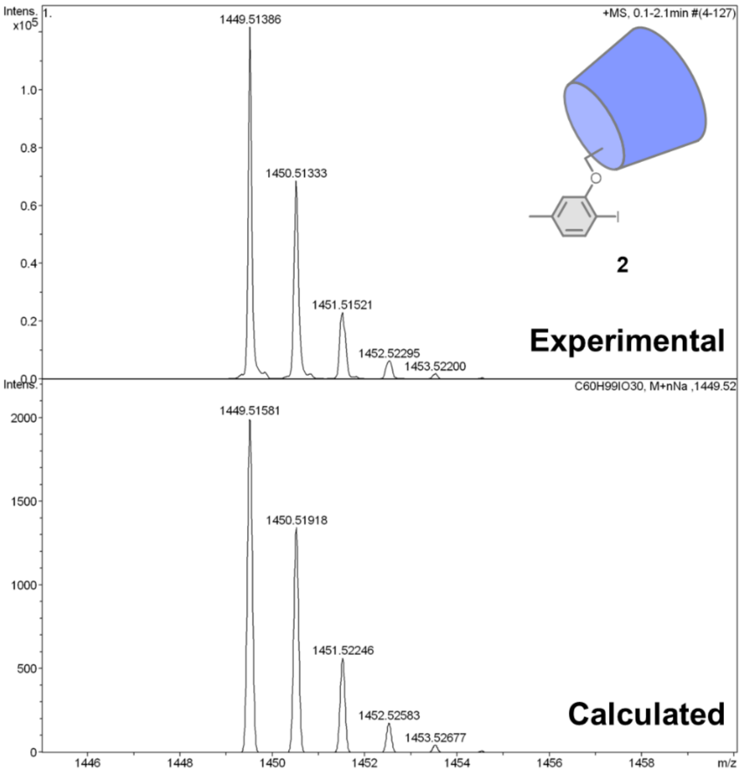

Figure S52. ESI-ToF-MS spectrum of **2**.

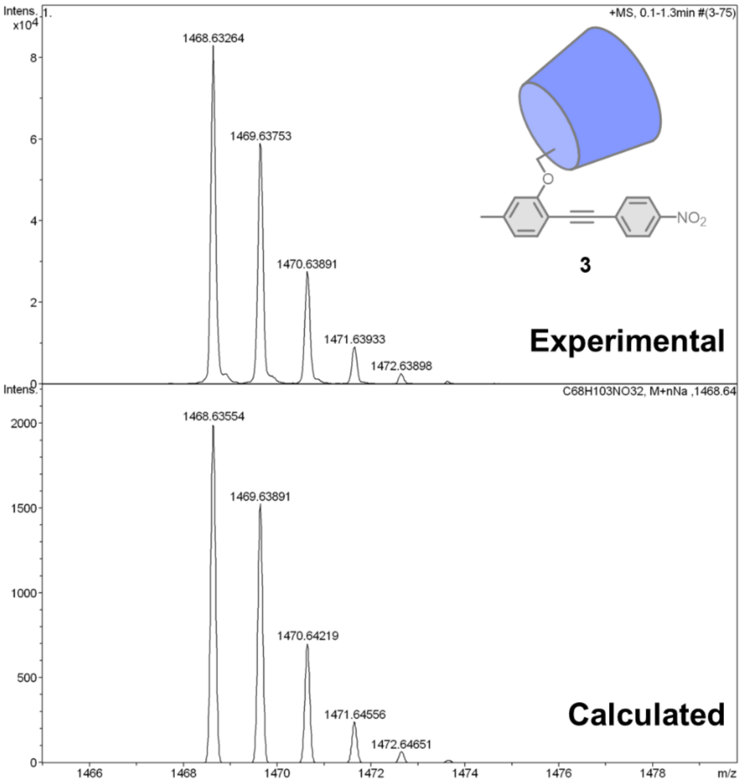

Figure S53. ESI-ToF-MS spectrum of **3**.

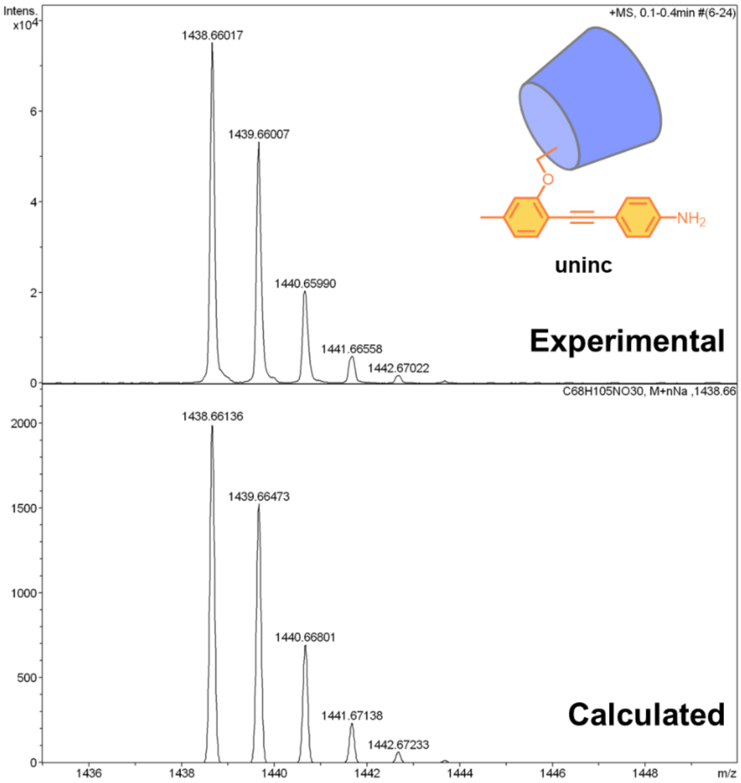

Figure S54. ESI-ToF-MS spectrum of **uninc**.

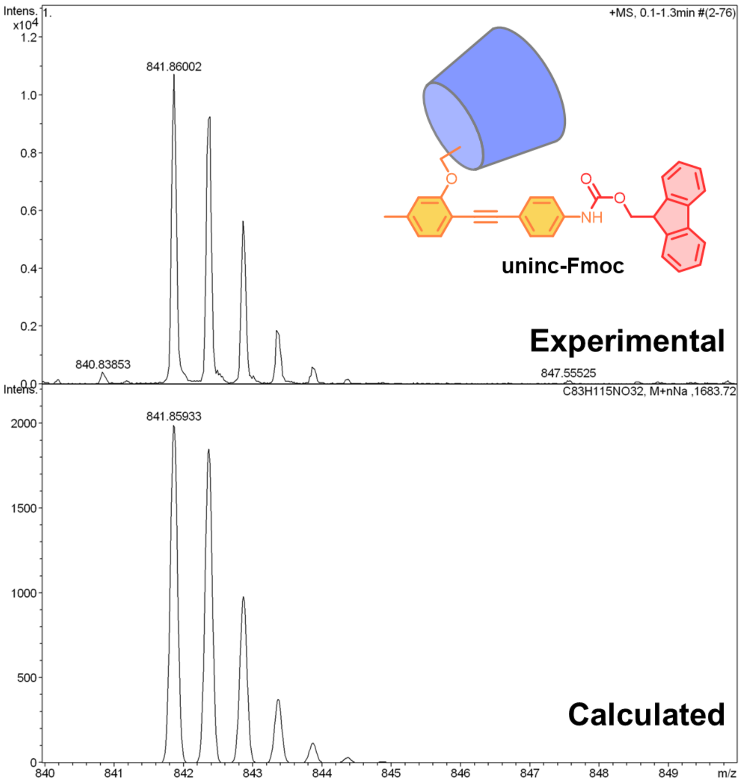

Figure S55. ESI-ToF-MS spectrum of **uninc-Fmoc**.

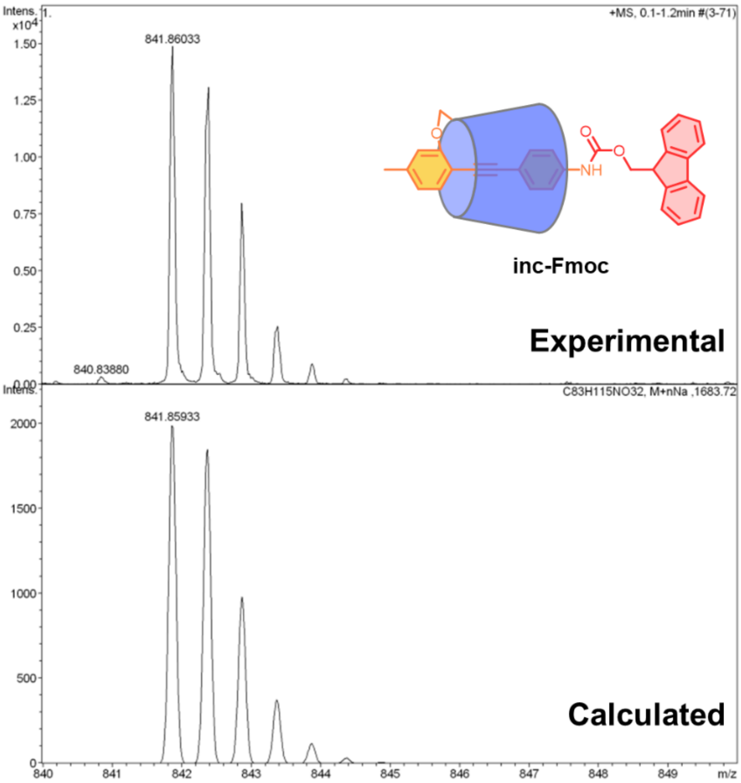

Figure S56. ESI-ToF-MS spectrum of **inc-Fmoc**.

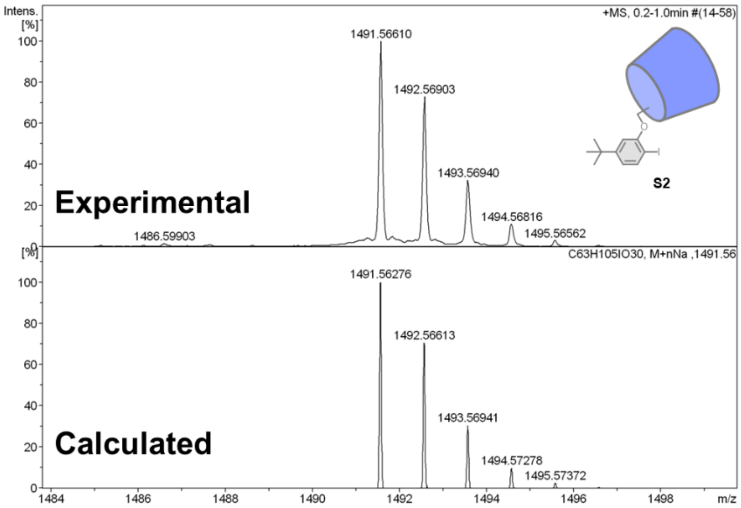

Figure S57. ESI-ToF-MS spectrum of **S2**.

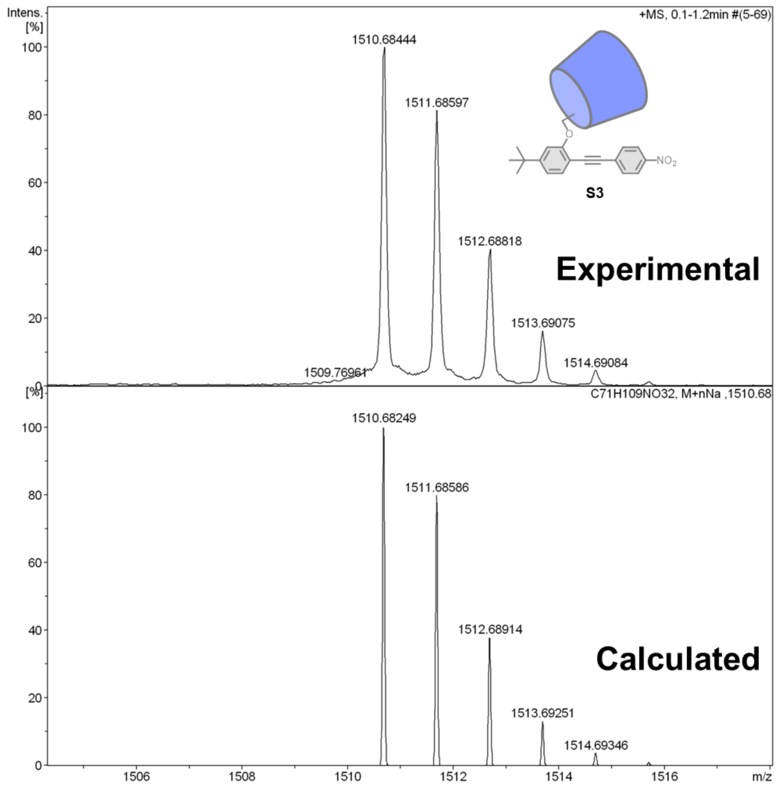

Figure S58. ESI-ToF-MS spectrum of **S3**.

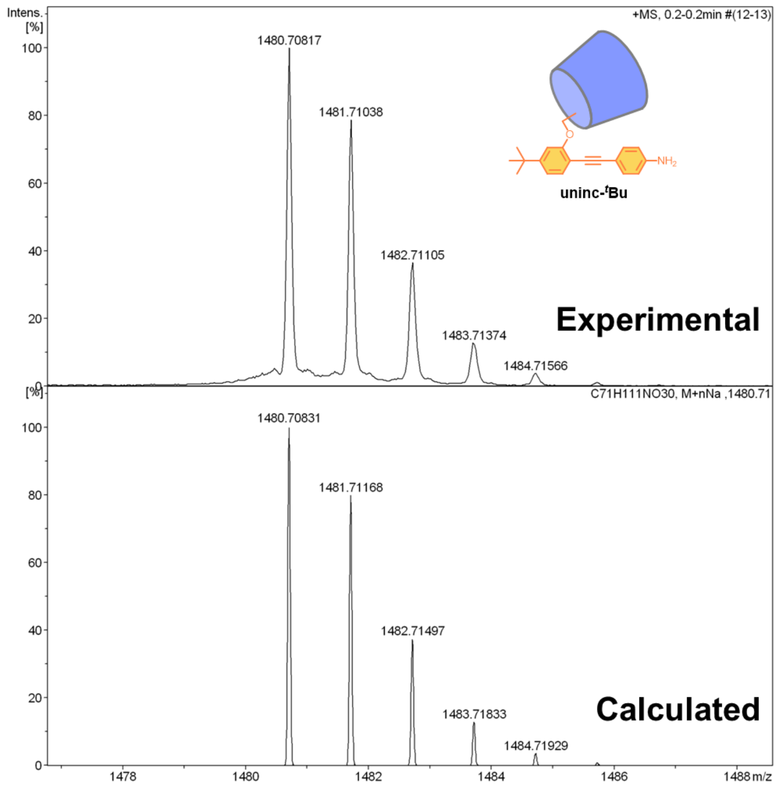

Figure S59. ESI-ToF-MS spectrum of **uninc-^{t}Bu**.

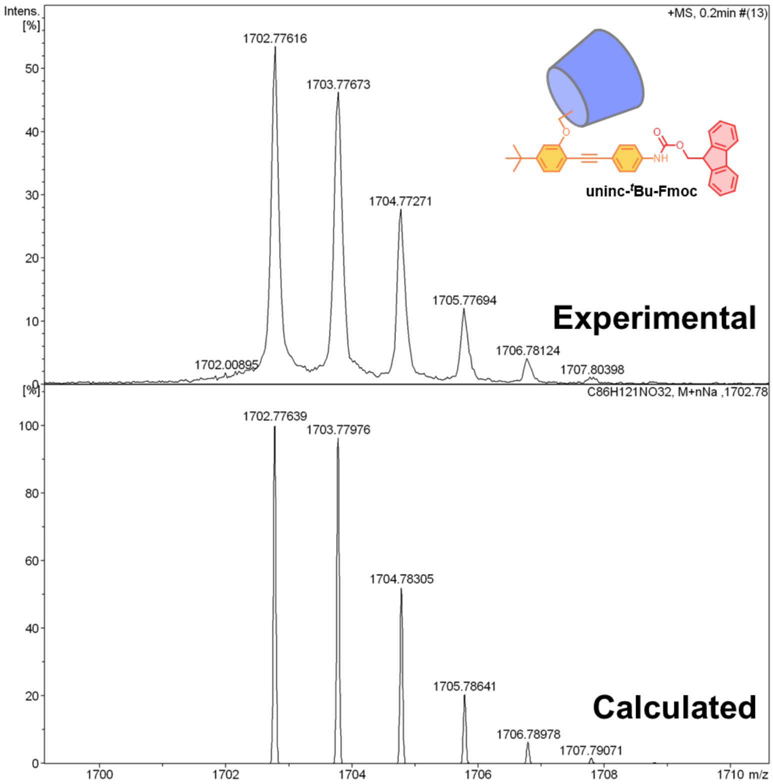

Figure S60. ESI-ToF-MS spectrum of **uninc-*t*Bu-Fmoc**.

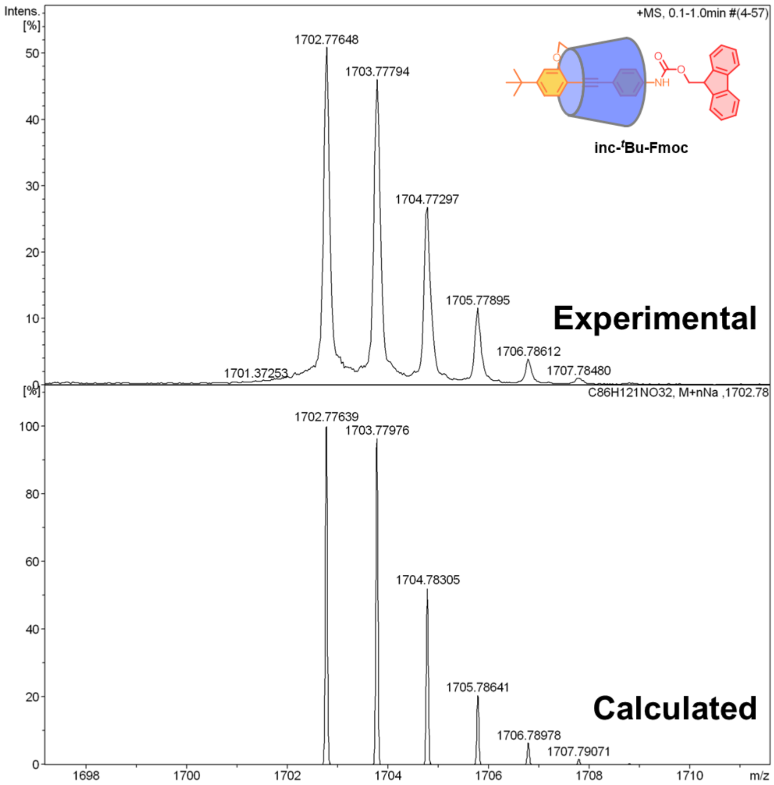

Figure S61. ESI-ToF-MS spectrum of **inc-*t*Bu-Fmoc**.

5. References

(1) Pangborn, A. B.; Giardello, M. A.; Grubbs, R. H.; Rosen, R. K.; Timmers, F. J. Safe and Convenient Procedure for Solvent Purification. *Organometallics* **1996**, *15* (5), 1518–1520.

(2) Taoda, Y.; Akiyama, T.; Tomita, K.; Fujiwara-Kitamura, M.; Tamura, Y.; Kawasuji, T.; Matsuoka, E.; Akihisa, E.; Seki, T.; Yoshinaga, T. Discovery of Tricyclic HIV-1 Integrase-LEDGF/P75 Allosteric Inhibitors by Intramolecular Direct Arylation Reaction. *Bioorg. Med. Chem. Lett.* **2022**, *64*, 128664.

(3) Sharber, S. A.; Baral, R. N.; Frausto, F.; Haas, T. E.; Müller, P.; Thomas, S. W., Iii. Substituent Effects That Control Conjugated Oligomer Conformation through Non-Covalent Interactions. *J. Am. Chem. Soc.* **2017**, *139* (14), 5164–5174.

(4) Kaneda, T.; Fujimoto, T.; Goto, J.; Asano, K.; Yasufuku, Y.; Jung, J. H.; Hosono, C.; Sakata, Y. New Large-Scale Preparations of Versatile 6-O-Monotosyl and 6-Monohydroxy Permethylated α-, β-, and γ-Cyclodextrins. *Chem. Lett.* **2002**, *31* (5), 514–515.

(5) Doad, G. J. S.; Barltrop, J. A.; Petty, C. M.; Owen, T. C. A Versatile and Convenient Synthesis of Benzofurans. *Tetrahedron Lett.* **1989**, *30* (13), 1597–1598.

(6) Newville, M.; Stensitzki, T.; Allen, D. B.; Ingargiola, A. *LMFIT: Non-Linear Least-Square Minimization and Curve-Fitting for Python*; 2014. https://doi.org/10.5281/zenodo.11813.
